# 4R-cembranoid confers neuroprotection against LPS-induced hippocampal inflammation in mice

**DOI:** 10.1186/s12974-021-02136-9

**Published:** 2021-04-19

**Authors:** Luis A. Rojas-Colón, Pramod K. Dash, Fabiola A. Morales-Vías, Madeline Lebrón-Dávila, Pedro A. Ferchmin, John B. Redell, Geronimo Maldonado-Martínez, Wanda I. Vélez-Torres

**Affiliations:** 1 Department of Biochemistry, Universidad Central del Caribe School of Medicine, Av. Sta. Juanita, Bayamón, 00960 Puerto Rico; 2 Department of Neurobiology and Anatomy, McGovern Medical School, University of Texas Health Science Center at Houston, Houston, TX 77030 USA; 3 University of Puerto Rico Molecular Science Research Center, Av. Juan Ponce de León, San Juan, 00926 Puerto Rico

**Keywords:** Neuroinflammation, Mice, 4R, TNF-α, IL-1β, Akt1, CREB, α7 receptor

## Abstract

**Background:**

Chronic brain inflammation has been implicated in the pathogenesis of various neurodegenerative diseases and disorders. For example, overexpression of pro-inflammatory cytokines has been associated with impairments in hippocampal-dependent memory. Lipopolysaccharide (LPS) injection is a widely used model to explore the pathobiology of inflammation. LPS injection into mice causes systemic inflammation, neuronal damage, and poor memory outcomes if the inflammation is not controlled. Activation of the alpha-7 nicotinic receptor (α7) plays an anti-inflammatory role in the brain through vagal efferent nerve signaling. 4R-cembranoid (4R) is a natural compound that crosses the blood-brain barrier, induces neuronal survival, and has been shown to modulate the activity of nicotinic receptors. The purpose of this study is to determine whether 4R reduces the deleterious effects of LPS-induced neuroinflammation and whether the α7 receptor plays a role in mediating these beneficial effects.

**Methods:**

Ex vivo population spike recordings were performed in C57BL/6J wild-type (WT) and alpha-7-knockout (α7KO) mouse hippocampal slices in the presence of 4R and nicotinic receptor inhibitors. For in vivo studies, WT and α7KO mice were injected with LPS for 2 h, followed by 4R or vehicle for 22 h. Analyses of IL-1β, TNF-α, STAT3, CREB, Akt1, and the long-term novel object recognition test (NORT) were performed for both genotypes. In addition, RNA sequencing and RT-qPCR analyses were carried out for 12 mRNAs related to neuroinflammation and their modification by 4R.

**Results:**

4R confers neuroprotection after NMDA-induced neurotoxicity in both WT and α7KO mice. Moreover, hippocampal TNF-α and IL-1β levels were decreased with 4R treatment following LPS exposure in both strains of mice. 4R restored LPS-induced cognitive decline in NORT. There was a significant increase in the phosphorylation of STAT3, CREB, and Akt1 with 4R treatment in the WT mouse hippocampus following LPS exposure. In α7KO mice, only pAkt levels were significantly elevated in the cortex. 4R significantly upregulated mRNA levels of ORM2, GDNF, and C3 following LPS exposure. These proteins are known to play a role in modulating microglial activation, neuronal survival, and memory.

**Conclusion:**

Our results indicate that 4R decreases the levels of pro-inflammatory cytokines; improves memory function; activates STAT3, Akt1, and CREB phosphorylation; and upregulates the mRNA levels of ORM2, GDNF, and C3. These effects are independent of the α7 nicotinic receptor.

**Supplementary Information:**

The online version contains supplementary material available at 10.1186/s12974-021-02136-9.

## Background

Chronic neuroinflammation is a key feature of a number of neurological diseases and disorders, such as Alzheimer’s disease [1], Parkinson’s disease [2], ischemic stroke, depression [3], and traumatic brain injury [4]. Lipopolysaccharide (LPS), a bacterial endotoxin, has been widely used as an in vivo animal model for investigation into peripherally and centrally induced inflammation. LPS activates the TLR-4 intracellular signaling pathway, which regulates the nuclear translocation of NF-κB and the gene expression of pro-inflammatory cytokines [5–7]. Transfer of peripheral inflammation to the brain by intraperitoneal injection of LPS occurs through activation of vagal afferent nerves [8]. The brain’s rapid anti-inflammatory action involves the vagal efferent acetylcholine release and activation of the α7 nicotinic acetylcholine receptors on periphery inflammatory cells. Peripheral pro-inflammatory cytokines can also bind to their respective receptors in the brain endothelium and induce NF-κβ activation or can enter the brain through the blood-brain barrier-free circumventricular organs [9].

LPS increases the levels of plasma, hippocampal, and cortical pro-inflammatory cytokines [10–12] and impairs hippocampus-dependent learning and memory in mice [13, 14]. The hippocampus contains glial cells, such as microglia and astrocytes, that maintain normal physiological functions [15]. Abnormal activation of astrocytes and microglia leads to the secretion of pro-inflammatory proteins that increase the neuroinflammatory response [16]. Modulating microglial phenotypes can lower neuroinflammation and increase tissue repair activity [17]. Astrocytes can secrete neurotrophic factors involved in decreasing microglial activation and promoting neuronal survival [18].

4R-cembranoid (4R) is a natural diterpenoid compound found in soft corals and tobacco plants and known for its antimicrobial and neuroprotective activities [19]. Ex vivo and in vivo studies have shown that 4R significantly protects neurons against NMDA-induced neurotoxicity [20] and organophosphate poisoning [21, 22]. In a rat model of ischemic stroke, 4R administered 1 h after reperfusion resulted in a 60% reduction in the infarct size compared with vehicle [23]. Furthermore, 4R treatment significantly reverses the motor disabilities associated with Parkinson’s disease in rats [24]. The 4R concentration in the brain is higher than in plasma when administered parenterally to rats [25].

Nicotine administration decreases pro-inflammatory cytokine release from specific brain regions, such as the striatum, hippocampus, and cerebral cortex, in rats receiving an intracerebroventricular injection of LPS. This effect was reversed by antagonists of the α7 receptor but not by antagonists of the α4β2 receptor [26]. In a traumatic brain injury model, α7-knockout (α7KO) mice had significantly higher plasma levels of TNF-α and IL-1β than WT mice [27]. Furthermore, previous studies have shown that 4R is an antagonist of neuronal acetylcholine receptors. Incubation with 4R inhibited carbamoylcholine-induced currents in cells expressing human α4β2 and α3β4 in a noncompetitive manner [28]. 4R also inhibited acetylcholine-evoked currents in cells transfected with human α7 nicotinic acetylcholine receptors [29].

The present study examines the neuroprotective effect of 4R during LPS-induced brain inflammation. It also considers the role of the α7 receptors in this effect and explores possible mechanisms of action of 4R.

## Methods

### Materials

The cembranoid 4R was prepared by Dr. K. El Sayed (School of Pharmacy, University of Louisiana, Monroe, LA) as previously described [30]. The purity of the batch used for these experiments was more than 98%. The 4R solvent consisted of 99.6% polyethylene glycol (PEG) and 0.4% dimethyl sulfoxide (DMSO). LPS from *Escherichia coli* serotype 026:B6 was purchased from Sigma-Aldrich (St. Louis, MO) and freshly dissolved in sterile saline (0.9% NaCl). N-Methyl-d-aspartic acid (NMDA) and dihydro-β-erythroidine (DHβE) were purchased from Sigma-Aldrich. Methyllycaconitine (MLA) was purchased from Calbiochem (La Jolla, CA).

### Animals

Three C57BL/6J α7-knockout and three heterozygous breeding pairs were obtained from Jackson Laboratories (Stock # 003232, Bar Harbor, ME) and bred in the Universidad Central del Caribe animal facility in a temperature-controlled room with free access to food and water and under a 12-hour light/dark cycle. The α7KO mice were genotyped by PCR using the primers recommended by Jackson Laboratories in order to select the null (−/−) and WT mice for experiments. All mice used were males and females 8–10 weeks of age. The Institutional Animal Care and Use Committee at UCC approved all mouse protocols.

### Hippocampal slice recordings

Mouse brains were removed, and the hippocampus dissected on ice and perfused with cold artificial cerebrospinal fluid (ACSF) saturated with 95% O_2_ and 5% CO_2_ [20]. The composition of the ACSF (in mM) was 125 NaCl, 3.3 KCL, 1.25 NaH_2_PO_4_, 2 MgSO_4_, 2 CaCl_2_, 25 NaHCO_3_, and 10 glucose. Transverse slices (400 μm thick) were obtained using a manual slicer. The hippocampal slices were transferred in equal amounts to a three-lane recording chamber, each with a perfusion line exposed to ACSF, 95% O_2_, and 5% CO_2_ at 34.5^o^C. A maximum of seven hippocampal slices was analyzed per lane per experimental condition. An electrode was placed in the stratum radiatum of the CA1 area to stimulate the Shaffer collaterals, and another electrode was placed in the stratum pyramidale to record the stimulus-evoked population spike (PS). The strength of the stimulus used was twice the strength required to elicit a threshold response. The PS was analyzed using the LABMAN program (gift from Dr. T.J. Teyler to Dr. PA Ferchmin, WWAMI Medical Education Program, University of Idaho, Moscow, ID). The percent PS area (msec x mV) was obtained by dividing the PS during treatment with the PS during perfusion with ACSF only. The loss or recovery of the PS area was used as an indicator of neurotoxicity or neuroprotection, respectively.

The first experimental condition was the control treatment followed by the effect of 4R before and after NMDA toxicity. The NMDA concentration and time were set to allow a 20% PS recovery in those slices treated with NMDA only. NMDA was removed by washing the slices with ACSF for 30 min. These three experimental conditions were carried out in hippocampal slices from WT and α7KO mice. Two more experimental conditions included the presence of two inhibitors, DHβE and MLA, at concentrations selective for the α4 and α7 subunit-containing nicotinic acetylcholine receptors, respectively. At the end of the experimental treatments and after 1 h recovery with ACSF, the final PS was recorded using the same stimulus strength and position as the initial PS.

### Sample collection

Mice were randomly divided into five groups: 1) saline, 2) 4R, 3) LPS/saline, 4) LPS/vehicle, and 5) LPS/4R. Mice were injected intraperitoneally (i.p.) with saline (0.9% NaCl) or LPS (5 mg/kg). 4R (6 mg/kg) or vehicle (99.6% PEG/0.4% DMSO) was injected subcutaneously (s.c.). In groups 3, 4 and 5, mice received LPS for 2 hours and at the end of 2 hours were injected with either saline, vehicle or 4R. All mice were sacrificed 24 h post first injection. Blood was collected by retro-orbital bleeding using capillary tubes containing heparin as an anti-coagulant (VWR; cat. # 15401-560). Samples were centrifuged at 10,000×*g* at 4 °C for 10 min and plasma collected and stored at − 80 °C until further analysis. Brains were removed and placed in cold phosphate-buffered saline (PBS, 10 mM, pH 7.4). A portion of the cortex and both hippocampus were removed, immediately frozen on dry ice, and stored at – 80 °C.

The tissue samples (1 mL/100 mg) were lysed in cold homogenization buffer containing 0.5% sodium deoxycholate, 100 mM Tris hydrochloride (pH = 8.3), 150 mM NaCl, 10 mM EDTA, 0.1% SDS, 10% glycerol, 1% Triton X-100, and 2% of a cocktail of protease inhibitors (Sigma-Aldrich; cat. # P8340) and phosphatase inhibitors (Sigma-Aldrich; cat. # P0044). Samples were homogenized on ice using disposable tissue-homogenizing tubes (Kimble; cat. # 749625-0010) and a plastic pestle to disrupt the tissue. Disrupted tissue samples were left on ice and vortexed every 2 min for 20 min. The supernatant was aliquoted in two 1.5-mL tubes and stored at − 80°C for future analysis. Total protein concentration was determined using the Precision Red Advanced protein assay (Cytoskeleton; cat. # ADV02-A).

### Cytokine measurements

Plasma and tissue homogenates were assayed for the cytokines IL-1β and TNF-α using the DuoSet ELISA kit from R&D Systems following the manufacturer’s instructions (mouse IL-1β/IL-1F2, cat. # DY401-05; mouse TNF-α, cat. # DY410-05). Briefly, 50 μg of protein was diluted and added to the antibody-containing wells in duplicate for 2 h at room temperature. Subsequently, the detection antibody–streptavidin–HRP complex was added following the substrate solution containing the color reagents. The absorbance was determined at 450 and 570 nm using a plate reader (SpectraMax ID3; Molecular Devices). The final absorbance was calculated by subtracting the 570-nm values from the 450-nm values. Standard curves (IL-1β, 15.6–1000 pg/ml; TNF-α, 31.2–2000 pg/ml) were used to quantify the concentrations of cytokines in pg/mL. The log10 of the standard curve values were plotted, and a four-parameter logistic curve was generated to determine the cytokine concentration (pg/mL) from the measured absorbance.

### Novel object recognition test

The Novel Object Recognition test (NORT) was performed in a black wooden box (30 cm × 30 cm × 30 cm) with a light (15 lux) in the center of the box following the protocol of Leger et al., 2013 [31]. The test consisted of three sessions. Before each session, mice were transported into the behavior-testing facility and were left inside for 30 min. First, there was a 10-min habituation session once a day for 3 days, in which the mice were placed in the empty box to expose them to the testing room and experimental conditions. This was done in order to reduce procedure-related stress, which can affect exploratory behavior. Twenty-four hours later, a familiarization session was carried out. Two identical objects were placed inside the box, and the mice explored both objects freely for 10 min. Exploratory behavior is defined as directing the nose towards the object at a distance of ≤ 2 cm. After the familiarization session, the mice received one of the five treatments described under Sample Collection. The only difference here was the LPS concentration, which was 1 mg/kg instead of 5 mg/kg. We reduced the concentration of LPS, because mice injected with 5 mg/kg showed low exploration activity due to sickness behavior. The LPS concentration of 1 mg/kg was enough to induce memory deficits in the NORT without altering mouse locomotor activity [14].

The testing session was performed 24 h post LPS treatment, a time at which the hippocampus has been shown to play a role [32]. Mice were placed in the box with one of the objects used in the familiarization step and with a new object. The time spent exploring was recorded in the familiarization and testing sessions. The box and objects were cleaned with 70% alcohol before and after each behavioral evaluation to avoid any olfactory cues. Videos were coded and scored by the investigator blinded to the treatments. The percent recognition index was used as a memory parameter using the following formula: [(*T*_novel_ − *T*_familiar_)/(*T*_novel_ + *T*_familiar_)] × 100, where *T* represents the length of time exploring the novel object vs. the familiar one. Locomotor activity was also measured during the NORT by recording the total distance traveled (in cm) by each mouse. The data collected was analyzed using EthoVision XT V.13 video-tracking software (Leesburg, VA).

### Signaling protein measurements

Phosphorylated Akt1 (Ser 473), CREB (Ser 133), and STAT3 (Tyr 705) were analyzed in mouse cortex and hippocampus using a Millipore 96-well magnetic bead multiplex immunoassay (Akt1, cat. # 48-618MAG; CREB, cat. # 48-628MAG; STAT3, cat. # 48-623MAG). Briefly, magnetic beads with fluorescent antibodies against each phosphorylated protein were added to each well. Samples (25 μg of protein) and positive and negative controls were added in duplicate and left incubating overnight. The detection antibody was added into each well followed by streptavidin–phycoerythrin. The samples were analyzed using the Luminex MAGPIX instrument at 50 events (Luminex Corp). The median fluorescence intensity (MFI) of the phosphorylated form of the protein was divided by its respective amount of total protein for statistical analyses.

### Western blot assay

Wild-type mice were randomly divided into five groups of three. Supernatants (25 μg of total protein) of hippocampus and cortex from WT mice were mixed with sample buffer containing β-mercaptoethanol, heated to 95 °C for 10 min, and resolved in a 10% SDS-PAGE. The proteins were transferred onto Amersham Hybond ECL nitrocellulose membranes (GE Healthcare Bio-Sciences Corp., Pittsburgh, PA), blocked with 5% BSA in TBS, and then probed with respective primary antibodies from Cell Signaling Technology (Danvers, MA): Phospho-STAT3 (Tyr 705, 1:1000, Cat. #: 9145S), Total-STAT3 (1:2000, Cat. #: 4904S), Phospho-Akt1 (Ser 473, 1:1000, Cat. #: 9018S), Total-Akt1 (1:1000 Cat. #:75692S), Phospho-CREB (Ser 133, 1:1000, Cat. #: 9198S), Total-CREB (1:1000, Cat. #: 9197S), and GAPDH (1:1000, Cat. #: 97166S). The immunoreactive signals were visualized by Odyssey CLx Quantitative Fluorescent Imaging System with fluorescent secondary antibodies: Goat anti-Mouse IRDye® 680RD (1:25,000, Cat. #: 925-68070) or Goat anti-Rabbit IRDye® 800CW (1:25,000, Cat. #: 925-32211). Results were analyzed with Image Studio Lite Software (LI-COR Biotechnology, Lincoln, NE). GAPDH immunoreactive signal was used as the loading control.

### RNA sequencing analysis

Both hippocampi from each individual animal were combined and total RNA was extracted from saline-, LPS-, and LPS/4R-treated WT mice and LPS/4R-treated α7KO groups following the protocol of Chomczynski and Sacchi [33]. RNA quantification was measured using a Thermofisher NanoDrop spectrophotometer, (Waltham, MA), and RNA integrity was monitored by loading 2.5 μl of each sample into an Agilent 2100 bioanalyzer. One μg of total RNA from each sample was pooled with other samples from the same treatment group into a single tube. The pooled RNA samples were sequenced by the MACROGEN sequencing service using the Illumina next-generation sequencing platform with a HiSeq sequencer and the TruSeq library prep kit. Analysis of the data was conducted using Ingenuity Pathway Analysis (IPA, Qiagen, Germantown, MD) software.

### Real-time quantitative PCR analysis

Hippocampal RNA was isolated using the RNeasy Mini Kit (Qiagen, cat# 74104) from three mice per treatment. From the total RNA, 0.5 μg was used for cDNA synthesis following the RT^2^ First Strand Kit (Qiagen, cat # 330401) protocol. RT-qPCR was carried out to determine the mRNA expression using a custom RT^2^ Profiler™ PCR Array (Qiagen, cat # CLAM38358). The numbers in parenthesis represent the internal Qiagen gene detection codes for the following genes: orosomucoid 2 (ORM2; PPM04524A), Fos proto-oncogene (FOS; PPM02940C), epithelial cadherin (CDH1; PPM03652F), insulin-like growth factor 2 (IGF2; PPM03655A), coagulation factor XII (F12; PPM28974A), forkhead box A1 (FOXA1; PPM04764H), chemokine ligand 12 (CCL12; PPM02977E), complement component 3 (C3; PPM04471E), thrombospondin 1 (THBS1; PPM03098F), glial cell line–derived neurotrophic factor (GDNF; PPM04315F), calponin 1 (CNN1; PPM05033A), calcium-sensing receptor (CASR; PPM04823A), and glyceraldehyde 3-phosphate dehydrogenase (GAPDH; PPM02946E) as the reference gene.

Quantitative polymerase chain reaction (qPCR) was carried out using the CFX Connect real-time PCR detection system (Bio-Rad, cat. # 1855200). Each 96-well plate contained two samples run in triplicate. The threshold cycle (CT) value was obtained using CFX Maestro software (Bio-Rad; Hercules, CA), with the same baseline carried over between runs as recommended by the protocol. CT values were analyzed using the ΔΔCT method according to the manufacturer’s manual. GAPDH was used as the housekeeping gene for data normalization, and the saline group was used as the control for each 2^−ΔΔCt^ calculation. Quality controls used in the array were the reverse transcription control (RTC) and the positive PCR control (PPC).

### Statistical analysis

The data were analyzed by one-way or two-way analysis of variance (ANOVA), followed by Tukey’s multiple comparisons test, unless otherwise specified. The assumption of homogeneity of variances was verified using the Brown–Forsythe test for equality of group variances. When the variance between groups was not homogeneous, a nonparametric ANOVA followed by Dunn’s post hoc test was used. When comparing two experimental groups, a two-sample *t*-test was performed. Results are presented as mean ± S.E.M. and were analyzed using GraphPad Prism v8.4 software (Bellevue, WA). *P* values < 0.05 were considered statistically significant.

## Results

### 4R-cembranoid neuroprotection against NMDA in mouse hippocampal slices

**Fig. 1:**
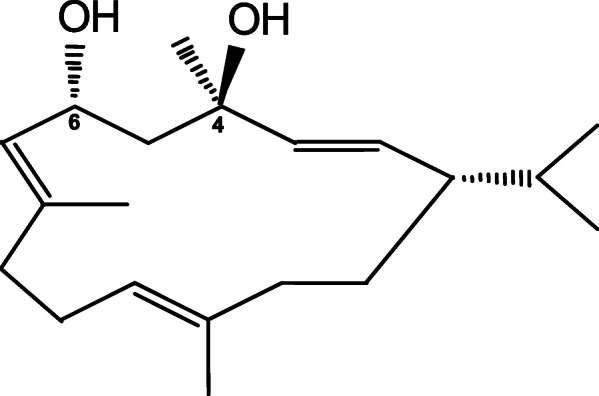
Structural formula of 4R

**Fig. 2:**
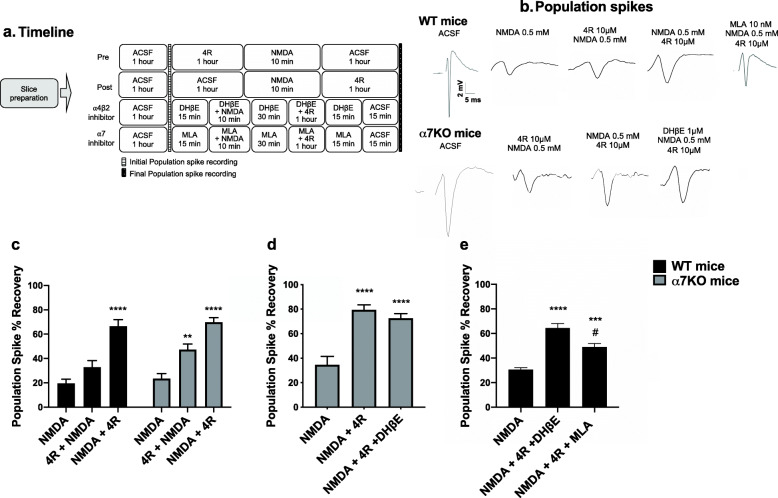
Hippocampal slices treated with 4R after NMDA insult showed recovery in both WT and α7KO mice. **a** Experimental timeline. **b** Representative final population spike recordings. **c** Population spike % recovery of 4R pre- and post-NMDA treatment in WT and α7KO mice. **d** % recovery in ⍺7KO mice with and without DHβE (1 μM). **e** % recovery in WT mice in the presence of DHβE (1 μM) or MLA (10 nM). Ordinary two-way ANOVA (**c**), ordinary one-way ANOVA (**d**), followed by Tukey’s post hoc test or nonparametric ANOVA test, followed by Dunn’s post hoc test (**e**). **p < 0.01, ***p < 0.001, **** *p* < 0.0001, versus NMDA; ^#^*p* < 0.05, versus NMDA+4R+DHβE. Data are presented as mean ± SEM. *n* = 14–21 slices/treatment from 2–3 independent experiments

Figure 1 shows the structure of 4R, which consists of a 14-carbon cembrane ring substituted with two hydroxy groups, attached to carbons 4 and 6, and three methyl groups. The hydrophobic ring and an electronegative atom at carbon 4 with R-chirality are known to be important for their neuroprotective activity [34]. To determine whether the neuroprotection previously reported in rats can be seen in mice, we prepared mouse hippocampal slices using a similar protocol [20] (Fig. 2a). As an example, a graphical representation of the percent population spike recovery for one experiment is shown in Fig. 2b. Figure 2c shows that 10 μM 4R applied after NMDA significantly increases the percent population spike (PS) recovery in both WT and α7KO mice. Wild-type mice recovered 62%, whereas α7KO mice recovered 70% of the NMDA-induced PS loss. The α7 and α4β2 receptors are the most abundant nicotinic acetylcholine receptors in the brain hippocampus [35]. Hippocampal slices from α7KO mice were treated with 1 μM DHβE, and at this concentration, DHβE selectively inhibits α4β2 neuronal receptors [36]. After incubation with 4R in the absence or presence of DHβE, the PS following NMDA toxicity significantly recovered (by 80% and 73%, respectively, Fig. 2d). No significant differences were observed between the slices treated with 4R and those treated with 4R and DHβE. To test whether 4R neuroprotection in hippocampal slices is mediated by the α7 receptor, 10 nM MLA was added to WT slices treated with NMDA + 4R (Fig. 2e). MLA at 10 nM selectively inhibits α7 acetylcholine receptors [36]. In the presence of MLA, the percent PS recovery was less than, but not significantly different from, the percent PS recovery without MLA (49% versus 62%, *p* = 0.09, *t*-test not shown). The percent PS recovery in WT slices treated with DHβE was also similar (69%, Fig. 2e) to the percent PS recovery without DHβE (62%, Fig. 2c, *p* = 0.62, *t*-test not shown). These results corroborate the neuroprotective role of 4R against NMDA neurotoxicity in mice, and point to a mechanism that is independent of the α7 and α4β2 nicotinic acetylcholine receptor pathways.

### Plasma and brain TNF-α and IL-1β levels

**Fig. 3:**
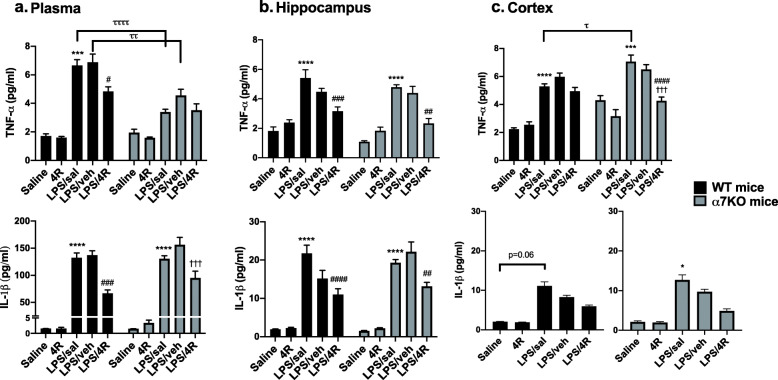
LPS-induced peripheral and central inflammation was reduced by 4R treatment. Levels of TNF-α and IL-1β cytokines were measured in WT and α7KO mice at 24 h. Cytokine levels in **a** plasma, **b** hippocampus, and **c** cortex. Two-way ANOVA followed by Tukey’s post hoc test. *p < 0.05, **p < 0.01, ***p < 0.001, ****p < 0.0001, versus saline; ^#^*p* < 0.05, ^##^*p* < 0.01, ^###^*p* < 0.001, ^####^*p* < 0.0001, versus LPS/sal; ^†††^*p* < 0.001, versus LPS/veh; ^τ^*p* < 0.05, ^ττ^*p* < 0.01, ^τττ^*p* < 0.001, comparison between mouse genotypes. Cortex IL-1β was analyzed using a nonparametric ANOVA test followed by Dunn’s post hoc test. Data are presented as mean ± SEM. *n* = 5 mice per group in duplicate

We examined TNF-α and IL-1β concentrations in plasma, hippocampus, and cerebral cortex in both WT and α7KO mice 24 h after LPS stimulation. As expected, TNF-α and IL-1β levels were significantly elevated in plasma, hippocampus, and cortex in the LPS/saline (sal)-treated mice compared with saline control in both WT and α7KO mice (Fig. 3). The LPS/veh-treated mice WT hippocampus IL-1β group mean tended to be lower than the LPS/sal group mean, but did not reach statistical significance. 4R alone (without LPS) had no effect on TNF-α and IL-1β levels in any of the areas examined and behaved like the saline control. To investigate the anti-inflammatory effect of 4R, 2 h after LPS administration (5 mg/kg) the mice were treated with 4R (6 mg/kg) or vehicle for 22 h. Plasma and hippocampal cytokine levels were significantly reduced in the WT LPS/4R groups compared with the LPS/sal group. A similar 4R effect was demonstrated in the α7KO mouse hippocampus. Moreover, in α7KO mice, 4R treatment lowered IL-1β levels in plasma and TNF-α levels in the cortex compared with the LPS/sal group. No 4R effect was noted in the WT mouse cortex. There was a treatment–genotype interaction in the plasma of LPS/sal and LPS/vehicle (veh) groups, as TNF-α levels in α7KO mice were lower than in WT. This was a surprise, since the anti-inflammatory vagus nerve activity in α7KO mice was not present. A genotype–treatment interaction was also observed in the cortex, as significantly higher TNF-α levels were noted in LPS/sal α7KO versus WT mice. These data indicate that 4R treatment reduces the levels of TNF-α and IL-1β in plasma and hippocampus of WT and α7KO mice. In the cortex, a lowering effect is observed in the TNF-α levels of α7KO mice.

### The 4R-cembranoid effect on long-term memory deficits is associated with LPS-induced neuroinflammation

**Fig. 4:**
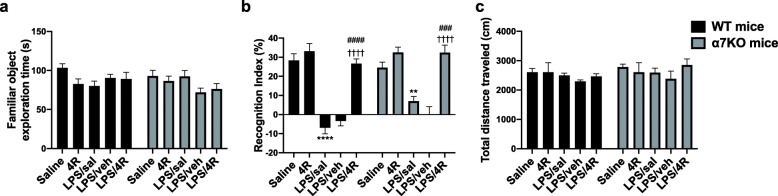
LPS-induced memory deficits were mitigated by 4R. **a** Exploration time during the familiarization step. **b** Recognition index % for WT and α7KO mice. **c** Locomotor activity was measured by the total distance traveled during NORT. Two-way ANOVA followed by Tukey’s post hoc test. **p < 0.01, ****p < 0.0001, versus saline; ^###^*p* < 0.001, ^####^*p* < 0.0001, versus LPS/sal; ^††††^*p* < 0.0001, versus LPS/veh. Data are presented as mean ± SEM. *n* = 5–9 mice per group

Next, we investigated the role of 4R on long-term memory function after an LPS challenge of 1 mg/kg in both WT and α7KO mice (Fig. 4). 4R alone had no effect on the novel object recognition index (RI) and performed similarly to the saline control group in both WT and α7KO mice. Analysis of the time mice spent exploring the objects during the familiarization phase showed no significant difference between treatment groups and genotypes. LPS-treated WT and α7KO mice had no exploratory preference for the novel object. LPS/sal-treated WT and α7KO groups had significantly lower RI than the saline controls. Interestingly, there was a tendency in the LPS/sal-treated α7KO mice to perform better than the LPS/sal-treated WT mice (*p* = 0.05). This suggests an adaptational change that affects the behavioral function in the α7KO mice when exposed to an insult. The loss of the α7 gene could potentially be compensated by increased transcription of other genes important for memory, such as the α4 and β2 genes. Both the α7 and the α4β2 nicotinic acetylcholine receptors have been shown to be important for hippocampal-dependent memory function [37]. On the other hand, Freund and colleagues demonstrated the importance of genetic background on hippocampal synaptic plasticity [38]. The authors showed that the hippocampal long-term potentiation in the α7KO C57/Bl6 mice was normal, but it was impaired in α7KO mice maintained in the C3H background. No genotype effect in NORT performance was observed in the saline- or 4R-only-treated groups. 4R administration significantly increased the percent RI in both WT (RI = 27%) and α7KO (RI = 32%) mice to baseline levels (WT, RI = 28%; α7KO, RI = 22%). 4R rescued the memory impairments induced by LPS through a mechanism independent of the α7 acetylcholine receptor. Neither treatment nor genotype altered the locomotor activity measured by the distance traveled in an open field during NORT. These data show that the detrimental effects of LPS on memory function were corrected by 4R treatment.

### STAT3, Akt1, and CREB phosphorylation in cortex and hippocampus

**Fig. 5:**
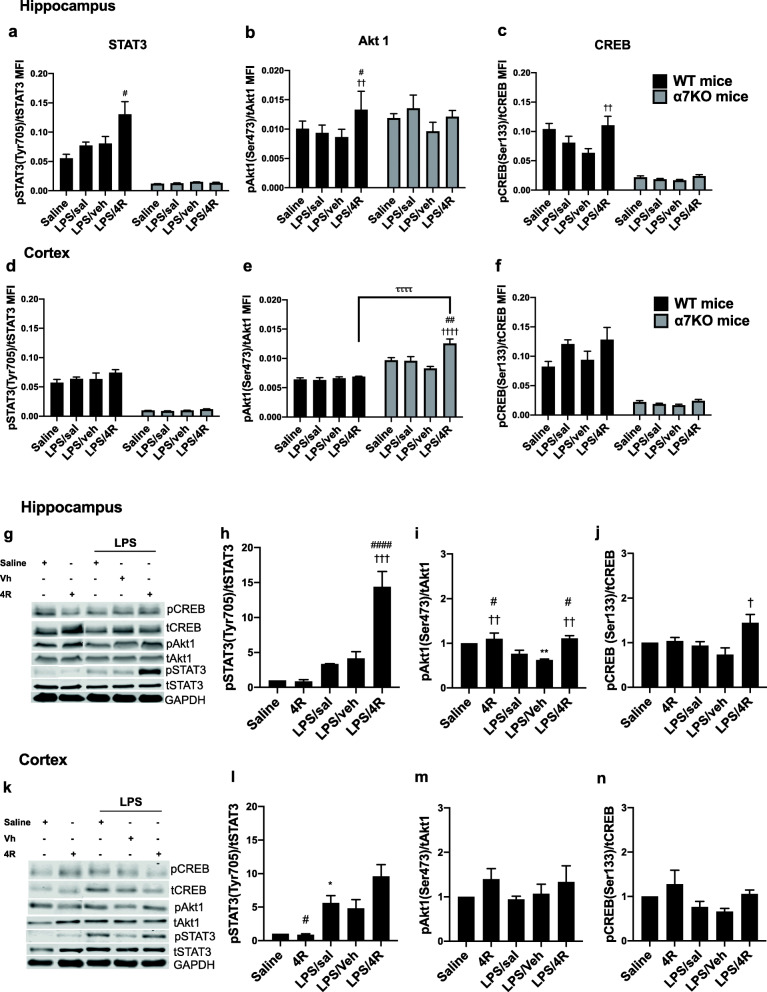
Effect of 4R on STAT3, Akt1, and CREB phosphorylation in WT and α7KO mice. Each graph represents the phosphorylated form normalized by total protein. **a**–**c** Hippocampus median fluorescence intensity (MFI) in both WT and α7KO mice. **d–f** Cortex MFI in both mouse strains. **a**, **d** pSTAT3 at Tyr 705. **b**, **e** pAkt1 at Ser 473. **c**, **f** pCREB at Ser 133. **g**–**n** Protein expression of pSTAT3, pAkt1, and pCREB in WT mice analyzed by Western blot in the hippocampus (**g**–**j**), and cortex (**k**–**n**) at the same phosphorylation sites. **a**–**f** Two-way ANOVA followed by Tukey’s post hoc test. **g**–**n** One-way ANOVA followed by Tukey’s post hoc test. *p < 0.05, **p < 0.01 versus saline; ^#^*p* < 0.05, ^##^*p* < 0.01, ^####^*p* < 0.0001 versus LPS/sal; ^†^*p* < 0.05, ^††^*p* < 0.01, ^†††^*p* < 0.001, ^††††^*p* < 0.0001, versus LPS/veh; ^**ττττ**^*p* < 0.0001, LPS/4R WT versus LPS/4R α7KO mice. Data presented as mean ± SEM. *n* = 5 mice per group in duplicate, for Western blot *n* = 3 per group

Total and phosphorylated amounts were measured in the hippocampus and cortex of WT and α7KO mice at 24 h under our experimental conditions (Fig. 5). We did not observe a significant LPS effect on the phosphorylation of these proteins, only a trend to increase pCREB when compared with saline control in WT mice cortex. Others have reported a significant reduction in pCREB 24 h after an LPS dose of 3 mg/kg i.p. in mouse hippocampus [39] and an increase in pSTAT3 24 h after an intracerebroventricular LPS injection [40]. In our hands, 4R treatment significantly increased Akt1, STAT3, and CREB phosphorylation in WT mouse hippocampus compared with LPS/sal- or LPS/veh-treated groups (Fig. 5a–c). 4R treatment did not affect WT cortex STAT3, Akt1, or CREB phosphorylation (Fig. 5d–f). The only significant effect of 4R in α7KO cortex was an increase in Akt1 phosphorylation when compared with LPS/sal- or LPS/veh-treated groups. A genotype–treatment interaction was noted between the WT and α7KO LPS/4R-treated groups (Fig. 5e). A chronic absence of the α7 receptor eliminated the phosphorylation of STAT3 and CREB in both hippocampus and cortex, regardless of treatment. α7 receptor-mediated anti-inflammatory activity involves activation of JAK2–STAT3 signaling [41]. It is possible that the shutdown of the vagal efferent nerve activity in α7KO mice suppressed STAT3 signaling. Impaired IL-6–STAT3 signaling in α7KO mouse liver was also reported by Kimura and colleagues [42]. Activation of α7 receptors induces CREB phosphorylation by increasing intracellular Ca^2+^ and cAMP levels [43–45]. However, these are not the only intracellular signals that can increase CREB phosphorylation, and this may, in part, explain the lack of pCREB in α7KO mice.

Western blot analysis of STAT3, Akt, and CREB phosphorylation in WT mice showed similar results to those obtained with the multiplex immunoassay. We included a 4R only-treated group to see if 4R alone could have an effect on protein phosphorylation. LPS/4R-treated mice hippocampus had a significantly higher STAT-3 and CREB phosphorylation when compared with LPS/sal and/or LPS/veh-treated groups (Fig. 5h, j). Akt1 phosphorylation was lower in the LPS/veh group as compared with saline control. Both 4R alone and LPS/4R treatments increased Akt1 phosphorylation when compared with LPS/sal and LPS/veh groups (Fig. 5i). This implies a 4R effect on Akt1 phosphorylation under both normal and pro-inflammatory conditions. As observed with the immunoassay, the phosphorylation of Akt1 and CREB in the WT cortex was not statistically significant (Fig. 5m–n). The STAT3 phosphorylation was significantly higher in the LPS/sal group as compared with saline and 4R alone. There was an increase of phosphorylation of STAT3 as compared with LPS/veh effect in LPS/4R-treated mice cortex, but this increase did not reach statistical significance (*p* = 0.06) (Fig.5l). These findings suggest that 4R treatment following an LPS insult activates the CREB and STAT3 signaling pathways, and that 4R activates Akt1 with or without an LPS insult in the WT mice hippocampus.

### RNA sequencing analysis

**Fig. 6:**
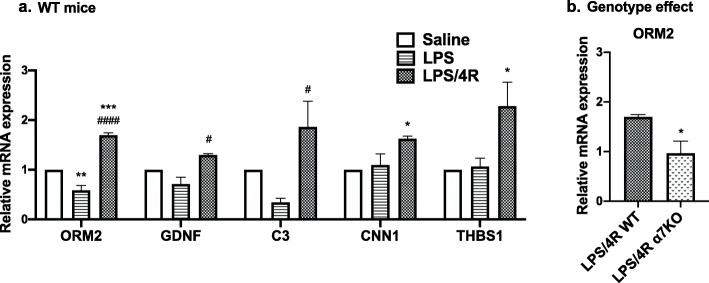
Hippocampal relative mRNA expression in WT mice 24 h after LPS exposure. **a** Relative mRNA expression of C3, THBS1, GDNF, CNN1, and ORM2 in WT mice. **b** ORM2 relative mRNA expression comparing WT and α7KO mice. Ordinary one-way ANOVA followed by Tukey post hoc test. *p < 0.05, **p < 0.01, ***p < 0.001, versus saline; ^#^*p* < 0.05, ^##^*p* < 0.01, ^####^*p* < 0.05, versus LPS. **b** *t*-test between WT and α7KO mice. *p < 0.05, versus LPS/4R WT. Data presented as mean ± SEM. *n* = 3 mice per group in triplicate

We performed RNA sequencing analysis for saline-, LPS-, and LPS/4R-treatment groups in the WT mouse hippocampus and in the LPS/4R-treatment group for α7KO mouse hippocampus. The mRNA signals obtained were analyzed and segmented into clusters using IPA software. Twelve mRNAs (see list in the “Methods” section) involved in neuroinflammation, neuronal death, and microglia activation with the highest fold change (FC = 2-fold upregulation or 0.5 downregulation) were selected and validated by RT-qPCR (Supplemental Table 1). Of the 12 mRNAs analyzed, 5 were significantly upregulated by 4R treatment (Fig. 6). The other 7 genes are presented in Supplemental Fig. 1aS. ORM2 mRNA expression was the only mRNA significantly downregulated by LPS (FC = 0.59) compared with saline. For each of the 5 genes, we calculated the 4R-induced fold change by dividing the mean mRNA expression in the LPS/4R-treatment group by the mean mRNA expression in the LPS-treatment group. 4R treatment significantly increased the mRNA levels of ORM2 (FC = 2.9), GDNF (FC = 1.9), and C3 (FC = 5.9) compared with LPS treatment (Fig. 6a). The mRNA levels in α7KO mice were not statistically different from WT (data shown in supplemental Fig. 1bS), except for the expression of ORM2. The α7KO mice had significantly lower ORM2 mRNA levels than WT (Fig. 6b). There was a weak 4R effect on the mRNA levels of THBS1 (FC = 1.9; *p* = 0.06) and CNN1 (FC = 1.5; *p* = 0.07) versus LPS. The RT-PCR data indicate that 4R increased the mRNA levels of ORM2, GDNF, and C3, and modestly increased the mRNA levels of THBS1 and CNN1 24 h after LPS exposure.

## Discussion

The ability of 4R to elicit neuronal survival was demonstrated in rat hippocampal slices using different neurotoxic agents, which showed that the neuroprotective effect of 4R was significant at concentrations of 0.2–40 μM and that 4R alone does not affect the NMDA receptor–mediated population spike (PS). 4R did not protect by blocking NMDA receptors but by activating the PI3K–Akt1 anti-apoptotic signaling pathway [20–22].

Our first aim was to confirm the neuroprotective effect of 4R in mouse hippocampal slices treated with NMDA. We demonstrated that 4R application after NMDA exposure significantly recovers the loss of neuronal population spikes (PS) in hippocampal slices of both WT and α7KO mice (Fig. 2). Although neuroprotection was also observed when 4R was applied before NMDA, it was more robust when applied after NMDA exposure. Co-incubation of 4R with DHβE after NMDA did not interfere with the neuroprotective action of 4R in WT or α7KO mice, which implies that the α4β2 acetylcholine receptors are not involved in the neuroprotective mechanism of 4R. Co-incubation of 4R with MLA after NMDA in WT hippocampal slices recovered the neuronal PS. Taken together our results show that the α4β2 and α7 nicotinic receptors are not involved in the 4R-mediated PS recovery when administered after NMDA in mice.

The main goal of our study was to investigate the neuroprotective role of 4R during CNS inflammation and the mechanisms for this effect. 4R treatment decreased TNF-α and IL-1β levels in WT and α7KO mouse hippocampus. Mice treated with 4R completely recovered from the memory deficits associated with LPS stimulation (Fig. 4). This was observed in both genotypes, demonstrating an α7-independent mechanism. Some of the detrimental effects of LPS on memory function have been attributed to IL-1β and TNF-α production. Li et al have reported that inhibition of IL-1β production in the dentate gyrus of the hippocampus improves cognitive function in mice [46]. IL-1β-silenced mice treated with LPS had a NORT recognition index similar to mice treated with saline. Using a different hippocampal-dependent learning test, Belardi and colleagues demonstrated that reduction of TNF-α mRNA levels in the hippocampus resulted in a significant improvement in the Morris water maze task in rats [47].

Next, we determined the effect of 4R on the phosphorylation status of STAT3, Akt1, and CREB. 4R treatment significantly increased the phosphorylation of Akt1, CREB, and STAT3 in the hippocampus of WT mice and Akt1 phosphorylation in the cortex of α7KO mice (Fig. 5). The PI3K–Akt1 signaling pathway is well recognized as one of the most important pathways modulating cell survival [48, 49]. We confirmed previous in vitro reports of Akt1 activation by 4R [23, 24]. Using an in vitro model of Parkinson’s disease, Hu and colleagues showed an increase in pAkt with 4R in a dose-dependent manner [24]. Martins and coworkers also reported an increase in pAkt in neuronal cells under normal conditions and during oxygen-glucose deprivation conditions [23]. In our study, we showed an increase in pAkt1 with 4R alone and with LPS/4R. Thus, 4R could protect hippocampal neurons against pro-apoptotic signals by increasing Akt phosphorylation in the absence or presence of an insult. Although full Akt1 activation requires phosphorylation of the S473 and T308 residues, phosphorylation of S473 stabilizes T308 phosphorylation and the active state of Akt1 [49].CREB activation stimulates neuronal survival and hippocampal long-term memory [44]. Activation of the α7 receptor increases CREB phosphorylation through Ca^2+^ entry and PKA phosphorylation [45], but our results show that the loss of this receptor abolishes CREB signaling.

STAT3 phosphorylation was significantly enhanced by 4R in the hippocampus of WT mice, although the role of STAT3 activation in inflammation and neuronal survival is less clear. It can induce pro- or anti-inflammatory responses, depending on the activation pathway (IL-6 or IL-10, respectively). Increased pSTAT3 in human neural progenitor cells treated with IL-1β and TNF-α to mimic brain inflammation showed inhibition of neuronal differentiation and stimulation of astrocyte differentiation, but the opposite effect of inhibition of astrocyte differentiation and stimulation of neuronal differentiation was observed when STAT3 signaling was silenced [50].

Gene expression analysis showed a significant upregulation of Orosomucoid 2 (ORM2), GDNF, and C3 mRNA levels with 4R versus LPS treatment. ORM2 protein is found in astrocytes, and it plays a role in controlling microglia activation during inflammation and neuronal survival [51]. Myungjin and collaborators showed that intracerebroventricular injection of ORM2 reduces neuroinflammation and microglial activation in the hippocampus and cortex 24 h after LPS stimulation [51]. ORM2 treatment partially reversed the hippocampal-dependent spatial memory deficits induced by LPS. The α7 acetylcholine receptor is involved in ORM2 expression, since our results showed lowered expression in α7KO versus WT mice. GDNF is secreted by astrocytes and has been shown to inhibit microglial activation in vitro [18]. Recent work demonstrated that GDNF and its receptor, GFRα1, are important for the maturation of adult-born dentate gyrus hippocampal neurons and spatial memory formation [52]. They also reported an increase in CREB phosphorylation in dentate gyrus–derived neuronal cells treated with GDNF but not in GFRα1 mutant cells, indicating a role for GDNF and GFRα1 in activating CREB. On the other hand, Mitroshina and coworkers reported a neuroprotective role for GDNF through Akt activation in mouse primary hippocampal neurons exposed to hypoxic conditions [53]. C3 factor is part of the complement system found in microglia and astrocytes. C3a is a peptide generated by factor C3 cleavage during complement activation. Co-stimulation of rat primary culture astrocytes with C3a and IL-1β increased nerve growth factor (NGF) protein expression [54]. C3a also protected mouse primary cortical astrocytes from chemical-induced ischemia by inhibiting caspase 3 cleavage and ERK phosphorylation [55].

Thrombospondin1 (THBS1) (also known as TSP-1) and CNN1 mRNA levels were modestly increased by 4R versus LPS treatment. CNN1 is a protein found in the mouse hippocampus that is important for vascular smooth muscle regulation [56]. THBS1 is an extracellular protein secreted by astrocytes that is important for vascular remodeling. THBS1-knockout mice displayed higher BBB disruption, neuronal death, and hippocampal-dependent learning impairments after traumatic brain injury [57]. Although our results need protein validation, they suggest that 4R may modulate astrocyte function to protect the hippocampus during inflammation.

Previous studies supported a direct neuroprotective effect of 4R in the brain, and 4R has been shown to decrease the infarct size in a rat transient ischemic stroke model [23] and to improve motor function in a rat model of Parkinson’s disease [24]. We have previously shown that intravenous injection of 4R reaches the brain within 10 min and that the 4R concentration in the brain was six times that in plasma [25]. When 4R was administered subcutaneously, its concentration in the brain was twice that in plasma due to a lower bioavailability. Our results demonstrate a 4R effect that is not mediated by α7 receptors as beneficial effects of 4R were unaffected in the α7KO mice. In summary, 4R-cembranoid has a potential neuroprotective value during pathological conditions where adverse immune responses and cognitive impairments are present such as in neurodegenerative diseases, brain injury, chronic stress, and aging [1, 10, 58].

## Conclusion

4R protects the hippocampus against inflammation and memory impairments triggered by LPS by lowering TNF-α and IL-1β levels and activation of the Akt1 and CREB signaling pathways. Astrocyte proteins involved in neuronal survival also seem to be modulated by 4R. The effects of 4R in the hippocampus are independent of α7 nicotinic receptors. The diagram below represents a model of 4R action in the hippocampus during inflammation. 
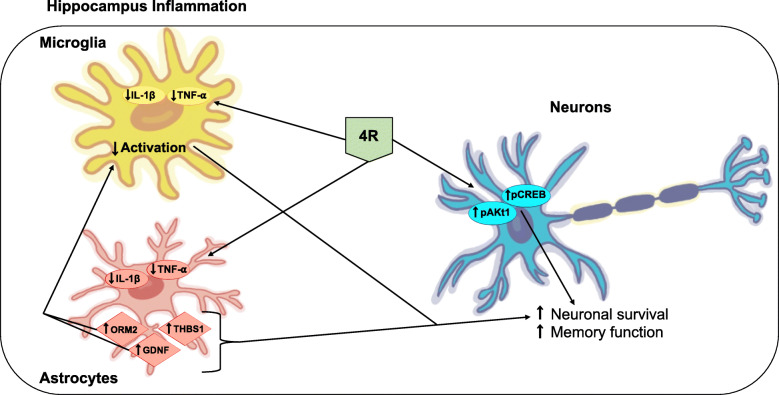


## Supplementary Information

**Additional file 1.**

**Additional file 2.**

## Data Availability

The data sets generated and/or analyzed during the current study are available from the corresponding author on reasonable request.

## Abbreviations

LPS: Lipopolysaccharide
IL-1β: Interleukin-1β
TNF-α: Tumor necrosis factor alpha
MCP-1: Monocyte chemoattractant protein 1
IL-6: Interleukin 6
NF-κb: Nuclear factor kappa-light-chain-enhancer of activated B cells
PEG: Polyethylane glycol
DMSO: Dimethyl sulfoxide
NMDA: N-Methyl-d-aspartate
DHβE: Dihydro-b-erythroidine
MLA: Methyllycaconitine
PS: Population spike
Akt1: Serine-threonine protein kinase 1
CREB: cAMP response element–binding protein
STAT3: Signal transducer and activator of transcription 3
ORM2: Orosomucoid 2
C3: Complement component 3
THBS1: Thrombospondin 1
GDNF: Glial cell line–derived neurotrophic factor
CNN1: Calponin 1
