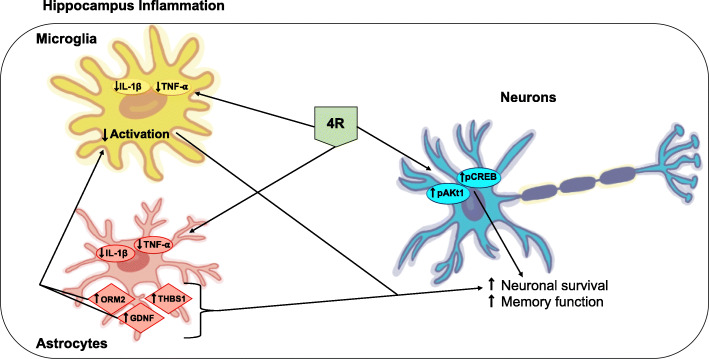# Correction to: 4R-cembranoid confers neuroprotection against LPS-induced hippocampal inflammation in mice

**DOI:** 10.1186/s12974-021-02159-2

**Published:** 2021-05-26

**Authors:** Luis A. Rojas-Colón, Pramod K. Dash, Fabiola A. Morales-Vías, Madeline Lebrón-Dávila, Pedro A. Ferchmin, John B. Redell, Geronimo Maldonado-Martínez, Wanda I. Vélez-Torres

**Affiliations:** 1 Department of Biochemistry, Universidad Central del Caribe School of Medicine, Av. Sta. Juanita, Bayamón, 00960 Puerto Rico; 2 Department of Neurobiology and Anatomy, McGovern Medical School, University of Texas Health Science Center at Houston, Houston, TX 77030 USA; 3 University of Puerto Rico Molecular Science Research Center, Av. Juan Ponce de León, San Juan, 00926 Puerto Rico

**Correction to: J Neuroinflammation 18, 95 (2021)**

https://doi.org/10.1186/s12974-021-02136-9

Following publication of the original article [1], the authors noticed that the image of the diagram model in the conclusion section was missing. Presented here is the correct image. The original article has been corrected.