# Supplementary figures and images for: 4R-cembranoid confers neuroprotection against LPS-induced hippocampal inflammation in mice
Source: J Neuroinflammation. 2021 Apr 19;18:95. doi: 10.1186/s12974-021-02136-9 (PMC8054431; doi:10.1186/s12974-021-02136-9)

## a. WT mice

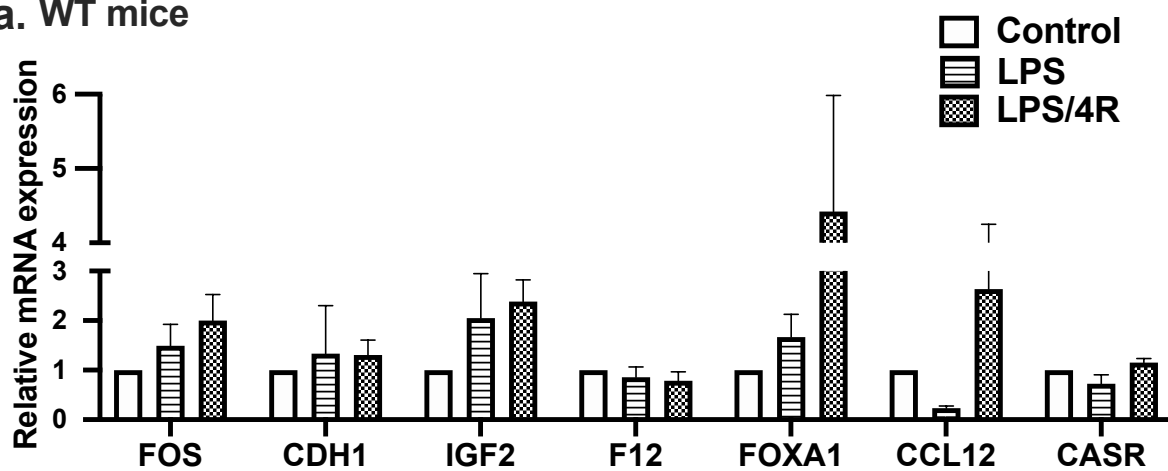

## b. Genotype effect

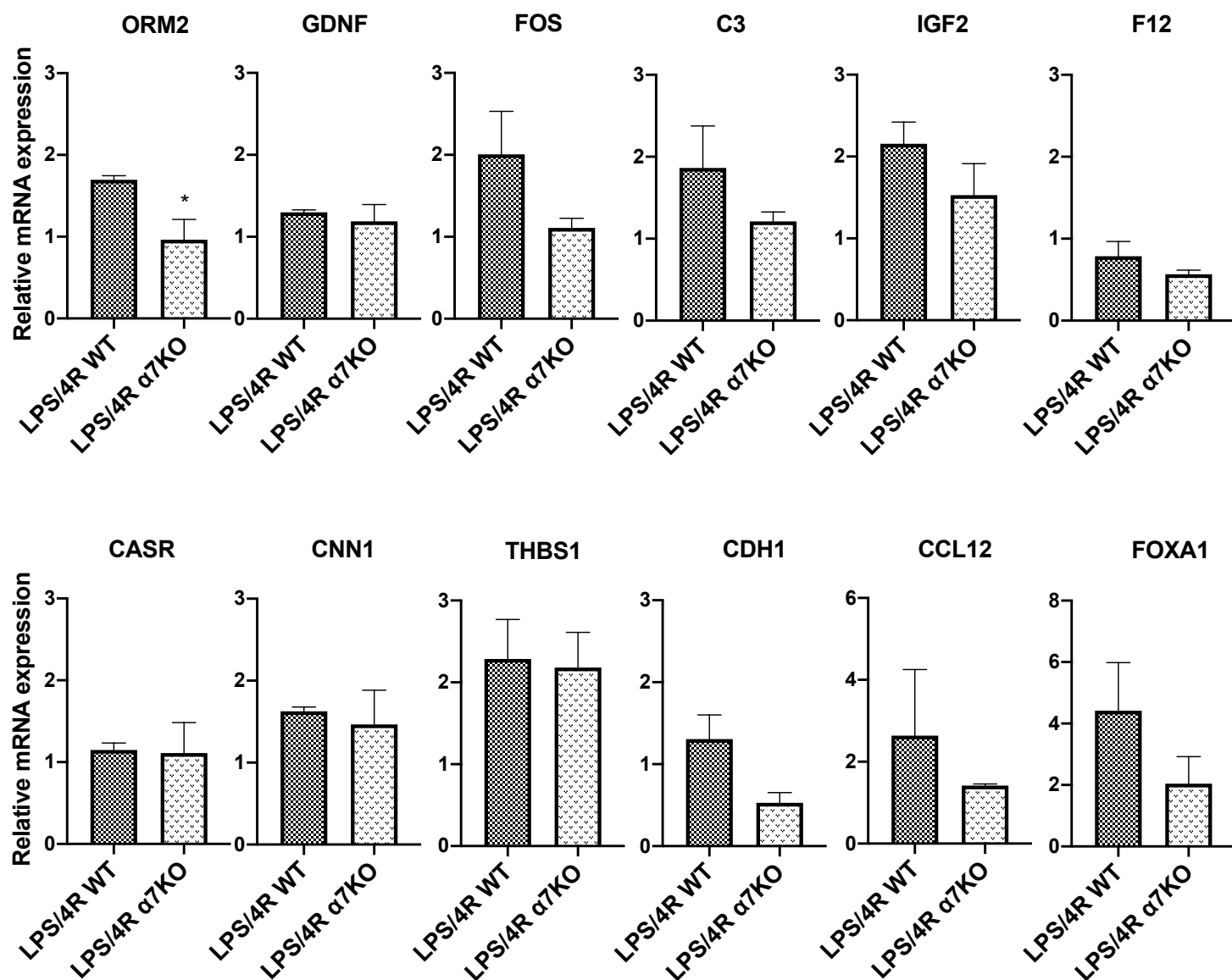

Supplement: Supplementary file 1 — Additional file 1. [file 12974_2021_2136_MOESM1_ESM.pdf]
