# Supplementary material for: 4R-cembranoid confers neuroprotection against LPS-induced hippocampal inflammation in mice
Source: J Neuroinflammation. 2021 Apr 19;18:95. doi: 10.1186/s12974-021-02136-9 (PMC8054431; doi:10.1186/s12974-021-02136-9)
Supplement: Supplementary file 2 — Additional file 2. [file 12974_2021_2136_MOESM2_ESM.docx]

**Supplementary Table 1.** Hippocampus mRNA displaying 2.0 higher, or 0.5 lower fold changes in differential expression between LPS over saline and LPS + 4R over LPS treatment.

|  | | **Counts per million (cpm)** | | | | **Fold Changes** | |
| --- | --- | --- | --- | --- | --- | --- | --- |
| **Gene** | **Description** | **WT saline** | **WT**  **LPS+sal** | **WT**  **LPS+4R** | **⍺7KO**  **LPS+4R** | **WT**  **LPS+sal/**  **saline** | **WT**  **LPS+4R/ LPS+sal** |
| Crtam | Crtam cytotoxic and regulatory T cell molecule | 0.03 | 0.08 | 2.12 | 1.29 | 2.82 | 27.10 |
| Tigit | Tigit T cell immunoreceptor with Ig and ITIM domains | 0.08 | 0.10 | 2.12 | 0.92 | 1.26 | 20.32 |
| Slc28a1 | Slc28a1 solute carrier family 28 (sodium-coupled nucleoside transporter), member 1 | 0.06 | 0.03 | 0.41 | 0.89 | 0.47 | 15.67 |
| Fga | Fga fibrinogen alpha chain | 0.11 | 0.05 | 0.82 | 0.63 | 0.47 | 15.67 |
| Tal2 | Tal2 T cell acute lymphocytic leukemia 2 | 0.11 | 0.03 | 0.38 | 0.20 | 0.24 | 14.69 |
| Tlcd2 | Tlcd2 TLC domain containing 2 | 0.39 | 0.03 | 0.38 | 0.29 | 0.07 | 14.69 |
| Apof | Apof apolipoprotein F | 0.06 | 0.03 | 0.38 | 0.37 | 0.47 | 14.69 |
| Cyp2j8 | Cyp2j8 cytochrome P450, family 2, subfamily j, polypeptide 8 | 0.06 | 0.03 | 0.36 | 0.17 | 0.47 | 13.71 |
| Tesmin | Tesmin testis expressed metallothionein like | 0.08 | 0.03 | 0.33 | 0.20 | 0.31 | 12.73 |
| Lox | Lox lysyl oxidase | 0.47 | 0.60 | 7.28 | 6.47 | 1.27 | 12.14 |
| Lrit3 | Lrit3 leucine-rich repeat, immunoglobulin-like and transmembrane domains 3 | 0.03 | 0.10 | 1.23 | 0.35 | 3.77 | 11.75 |
| Snord16a | Snord16a small nucleolar RNA, C/D box 16A | 0.08 | 0.05 | 0.59 | 0.29 | 0.63 | 11.26 |
| Txndc2 | Txndc2 thioredoxin domain containing 2 (spermatozoa) | 0.06 | 0.03 | 0.28 | 0.12 | 0.47 | 10.77 |
| Adra2b | Adra2b adrenergic receptor, alpha 2b | 0.33 | 0.03 | 0.28 | 0.29 | 0.08 | 10.77 |
| C8b | C8b complement component 8, beta polypeptide | 0.08 | 0.05 | 0.56 | 0.49 | 0.63 | 10.77 |
| Rrh | Rrh retinal pigment epithelium derived rhodopsin homolog | 0.47 | 0.13 | 1.35 | 0.26 | 0.28 | 10.38 |
| Xlr4b | Xlr4b X-linked lymphocyte-regulated 4B | 0.25 | 0.03 | 0.26 | 0.03 | 0.10 | 9.79 |
| AA619741 | AA619741 expressed sequence AA619741 | 0.06 | 0.03 | 0.26 | 0.12 | 0.47 | 9.79 |
| Brip1 | Brip1 BRCA1 interacting protein C-terminal helicase 1 | 0.06 | 0.03 | 0.26 | 0.26 | 0.47 | 9.79 |
| Ccl11 | Ccl11 chemokine (C-C motif) ligand 11 | 0.03 | 0.05 | 0.51 | 0.60 | 1.88 | 9.79 |
| Prr15l | Prr15l proline rich 15-like | 0.08 | 0.05 | 0.51 | 0.66 | 0.63 | 9.79 |
| Tulp1 | Tulp1 tubby like protein 1 | 0.22 | 0.10 | 0.97 | 0.66 | 0.47 | 9.31 |
| Igfbp1 | Igfbp1 insulin-like growth factor binding protein 1 | 0.06 | 0.05 | 0.49 | 0.35 | 0.94 | 9.31 |
| 1700001J03Rik | 1700001J03Rik RIKEN cDNA 1700001J03 gene | 0.08 | 0.05 | 0.49 | 0.46 | 0.63 | 9.31 |
| Glb1l3 | Glb1l3 galactosidase, beta 1 like 3 | 0.08 | 0.13 | 1.18 | 1.18 | 1.57 | 9.01 |
| Olfr1033 | Olfr1033 olfactory receptor 1033 | 0.08 | 0.05 | 0.46 | 0.20 | 0.63 | 8.82 |
| Rasl2-9 | Rasl2-9 RAS-like, family 2, locus 9 | 0.22 | 0.05 | 0.46 | 0.29 | 0.24 | 8.82 |
| Gm10389 | Gm10389 predicted gene 10389 | 0.03 | 0.03 | 0.23 | 0.06 | 0.94 | 8.82 |
| Alpk3 | Alpk3 alpha-kinase 3 | 0.78 | 0.42 | 3.58 | 3.16 | 0.54 | 8.57 |
| Gm6525 | Gm6525 predicted pseudogene 6525 | 0.11 | 0.05 | 0.43 | 0.12 | 0.47 | 8.33 |
| Il4i1 | Il4i1 interleukin 4 induced 1 | 0.11 | 0.08 | 0.64 | 0.20 | 0.71 | 8.16 |
| Acot10 | Acot10 acyl-CoA thioesterase 10 | 0.06 | 0.03 | 0.20 | 0.09 | 0.47 | 7.84 |
| Dbhos | Dbhos dopamine beta hydroxylase, opposite strand | 0.17 | 0.03 | 0.20 | 0.09 | 0.16 | 7.84 |
| Gata6 | Gata6 GATA binding protein 6 | 0.11 | 0.03 | 0.20 | 0.14 | 0.24 | 7.84 |
| Slco1b2 | Slco1b2 solute carrier organic anion transporter family, member 1b2 | 0.19 | 0.05 | 0.41 | 0.26 | 0.27 | 7.84 |
| Gpha2 | Gpha2 glycoprotein hormone alpha 2 | 0.06 | 0.03 | 0.20 | 0.29 | 0.47 | 7.84 |
| Ddx43 | Ddx43 DEAD (Asp-Glu-Ala-Asp) box polypeptide 43 | 0.11 | 0.08 | 0.61 | 0.26 | 0.71 | 7.84 |
| Slc17a9 | Slc17a9 solute carrier family 17, member 9 | 0.36 | 0.73 | 5.55 | 3.65 | 2.03 | 7.59 |
| Btbd18 | Btbd18 BTB (POZ) domain containing 18 | 0.14 | 0.08 | 0.59 | 0.55 | 0.56 | 7.51 |
| Tns4 | Tns4 tensin 4 | 1.03 | 0.10 | 0.77 | 0.49 | 0.10 | 7.35 |
| Asic3 | Asic3 acid-sensing (proton-gated) ion channel 3 | 0.72 | 0.63 | 4.60 | 4.03 | 0.87 | 7.35 |
| Hspb7 | Hspb7 heat shock protein family, member 7 (cardiovascular) | 0.19 | 0.05 | 0.38 | 0.35 | 0.27 | 7.35 |
| 4930505A04Rik | 4930505A04Rik RIKEN cDNA 4930505A04 gene | 0.19 | 0.05 | 0.38 | 0.49 | 0.27 | 7.35 |
| Ddx3y | Ddx3y DEAD (Asp-Glu-Ala-Asp) box polypeptide 3, Y-linked | 11.56 | 8.71 | 62.28 | 18.64 | 0.75 | 7.15 |
| Akap12 | Akap12 A kinase (PRKA) anchor protein (gravin) 12 | 16.63 | 18.94 | 135.24 | 77.19 | 1.14 | 7.14 |
| Asb14 | Asb14 ankyrin repeat and SOCS box-containing 14 | 0.11 | 0.29 | 1.99 | 1.55 | 2.59 | 6.95 |
| Tmem89 | Tmem89 transmembrane protein 89 | 0.08 | 0.05 | 0.36 | 0.12 | 0.63 | 6.86 |
| Ncapg | Ncapg non-SMC condensin I complex, subunit G | 0.22 | 0.05 | 0.36 | 0.20 | 0.24 | 6.86 |
| Treh | Treh trehalase (brush-border membrane glycoprotein) | 0.08 | 0.18 | 1.25 | 0.46 | 2.20 | 6.86 |
| Steap4 | Steap4 STEAP family member 4 | 0.50 | 1.57 | 10.73 | 7.08 | 3.14 | 6.86 |
| Prm1 | Prm1 protamine 1 | 0.06 | 0.03 | 0.18 | 0.06 | 0.47 | 6.86 |
| Ccl8 | Ccl8 chemokine (C-C motif) ligand 8 | 0.03 | 0.03 | 0.18 | 0.09 | 0.94 | 6.86 |
| Erich4 | Erich4 glutamate rich 4 | 0.03 | 0.03 | 0.18 | 0.12 | 0.94 | 6.86 |
| Hrasls5 | Hrasls5 HRAS-like suppressor family, member 5 | 0.03 | 0.03 | 0.18 | 0.12 | 0.94 | 6.86 |
| 1810007C17Rik | 1810007C17Rik RIKEN cDNA 1810007C17 gene | 0.08 | 0.03 | 0.18 | 0.12 | 0.31 | 6.86 |
| Tpsg1 | Tpsg1 tryptase gamma 1 | 0.19 | 0.03 | 0.18 | 0.12 | 0.13 | 6.86 |
| Gimap3 | Gimap3 GTPase, IMAP family member 3 | 0.22 | 0.03 | 0.18 | 0.12 | 0.12 | 6.86 |
| Rufy4 | Rufy4 RUN and FYVE domain containing 4 | 0.17 | 0.03 | 0.18 | 0.14 | 0.16 | 6.86 |
| Kif23 | Kif23 kinesin family member 23 | 0.44 | 0.44 | 2.91 | 1.01 | 1.00 | 6.57 |
| Spon2 | Spon2 spondin 2, extracellular matrix protein | 0.06 | 0.26 | 1.71 | 1.01 | 4.71 | 6.56 |
| Rsad2 | Rsad2 radical S-adenosyl methionine domain containing 2 | 0.83 | 3.26 | 21.06 | 13.20 | 3.92 | 6.46 |
| Wdr72 | Wdr72 WD repeat domain 72 | 0.91 | 0.10 | 0.66 | 0.32 | 0.11 | 6.37 |
| Il13ra2 | Il13ra2 interleukin 13 receptor, alpha 2 | 0.42 | 0.05 | 0.33 | 0.29 | 0.13 | 6.37 |
| Insrr | Insrr insulin receptor-related receptor | 0.42 | 0.05 | 0.33 | 0.32 | 0.13 | 6.37 |
| Fut1 | Fut1 fucosyltransferase 1 | 0.03 | 0.05 | 0.33 | 0.43 | 1.88 | 6.37 |
| Cfap53 | Cfap53 cilia and flagella associated protein 53 | 0.44 | 0.23 | 1.48 | 0.66 | 0.53 | 6.31 |
| Gm14207 | Gm14207 predicted gene 14207 | 0.11 | 0.18 | 1.15 | 0.63 | 1.65 | 6.30 |
| Jaml | Jaml junction adhesion molecule like | 0.22 | 0.16 | 0.97 | 1.18 | 0.71 | 6.20 |
| Skor1 | Skor1 SKI family transcriptional corepressor 1 | 0.28 | 0.21 | 1.25 | 1.09 | 0.75 | 6.00 |
| Cxcl9 | Cxcl9 chemokine (C-X-C motif) ligand 9 | 0.14 | 0.31 | 1.87 | 3.42 | 2.26 | 5.96 |
| Cytip | Cytip cytohesin 1 interacting protein | 0.03 | 0.13 | 0.77 | 0.46 | 4.71 | 5.88 |
| Acp4 | Acp4 acid phosphatase 4 | 0.06 | 0.08 | 0.46 | 0.14 | 1.41 | 5.88 |
| **Foxa1** | **Foxa1 forkhead box A1** | **0.03** | **0.03** | **0.15** | **0.03** | **0.94** | **5.88** |
| C9 | C9 complement component 9 | 0.03 | 0.03 | 0.15 | 0.03 | 0.94 | 5.88 |
| Mir674 | Mir674 microRNA 674 | 0.06 | 0.03 | 0.15 | 0.03 | 0.47 | 5.88 |
| Pde6a | Pde6a phosphodiesterase 6A, cGMP-specific, rod, alpha | 0.03 | 0.05 | 0.31 | 0.06 | 1.88 | 5.88 |
| Mir93 | Mir93 microRNA 93 | 0.03 | 0.03 | 0.15 | 0.09 | 0.94 | 5.88 |
| Chmp4c | Chmp4c charged multivesicular body protein 4C | 0.03 | 0.03 | 0.15 | 0.14 | 0.94 | 5.88 |
| Rgs1 | Rgs1 regulator of G-protein signaling 1 | 0.03 | 0.05 | 0.31 | 0.14 | 1.88 | 5.88 |
| Gm11413 | Gm11413 predicted gene 11413 | 0.14 | 0.03 | 0.15 | 0.17 | 0.19 | 5.88 |
| Mir6928 | Mir6928 microRNA 6928 | 0.17 | 0.05 | 0.31 | 0.17 | 0.31 | 5.88 |
| C030034L19Rik | C030034L19Rik RIKEN cDNA C030034L19 gene | 0.14 | 0.05 | 0.31 | 0.20 | 0.38 | 5.88 |
| Melk | Melk maternal embryonic leucine zipper kinase | 0.06 | 0.05 | 0.31 | 0.35 | 0.94 | 5.88 |
| Tpsb2 | Tpsb2 tryptase beta 2 | 0.58 | 0.03 | 0.15 | 0.43 | 0.04 | 5.88 |
| 1700109H08Rik | 1700109H08Rik RIKEN cDNA 1700109H08 gene | 1.41 | 1.30 | 7.36 | 4.43 | 0.92 | 5.64 |
| Cldn20 | Cldn20 claudin 20 | 0.30 | 0.10 | 0.59 | 0.40 | 0.34 | 5.63 |
| Rnf125 | Rnf125 ring finger protein 125 | 0.80 | 1.25 | 7.03 | 4.29 | 1.56 | 5.61 |
| Nmrk2 | Nmrk2 nicotinamide riboside kinase 2 | 0.11 | 0.18 | 1.02 | 0.20 | 1.65 | 5.60 |
| Accsl | Accsl 1-aminocyclopropane-1-carboxylate synthase (non-functional)-like | 0.22 | 0.26 | 1.46 | 1.18 | 1.18 | 5.58 |
| Atp1a4 | Atp1a4 ATPase, Na+/K+ transporting, alpha 4 polypeptide | 0.03 | 0.16 | 0.87 | 0.26 | 5.65 | 5.55 |
| Npc1l1 | Npc1l1 NPC1 like intracellular cholesterol transporter 1 | 0.94 | 0.76 | 4.19 | 4.69 | 0.80 | 5.54 |
| Mfsd2b | Mfsd2b major facilitator superfamily domain containing 2B | 1.44 | 1.44 | 7.87 | 6.44 | 1.00 | 5.49 |
| Gpr156 | Gpr156 G protein-coupled receptor 156 | 1.77 | 1.59 | 8.71 | 5.15 | 0.90 | 5.48 |
| Kdm5d | Kdm5d lysine (K)-specific demethylase 5D | 6.76 | 3.76 | 20.32 | 6.59 | 0.56 | 5.41 |
| Phex | Phex phosphate regulating endopeptidase homolog, X-linked | 0.30 | 0.10 | 0.56 | 0.83 | 0.34 | 5.39 |
| Aqp12 | Aqp12 aquaporin 12 | 0.06 | 0.05 | 0.28 | 0.03 | 0.94 | 5.39 |
| 4930414N06Rik | 4930414N06Rik RIKEN cDNA 4930414N06 gene | 0.03 | 0.05 | 0.28 | 0.14 | 1.88 | 5.39 |
| Ly6k | Ly6k lymphocyte antigen 6 complex, locus K | 0.33 | 0.05 | 0.28 | 0.23 | 0.16 | 5.39 |
| Prg4 | Prg4 proteoglycan 4 (megakaryocyte stimulating factor, articular superficial zone protein) | 2.63 | 4.28 | 22.95 | 19.97 | 1.63 | 5.36 |
| Sytl3 | Sytl3 synaptotagmin-like 3 | 0.25 | 0.08 | 0.41 | 0.17 | 0.31 | 5.22 |
| Gm9199 | Gm9199 predicted gene 9199 | 0.06 | 0.08 | 0.41 | 0.20 | 1.41 | 5.22 |
| Trim63 | Trim63 tripartite motif-containing 63 | 0.11 | 0.08 | 0.41 | 0.37 | 0.71 | 5.22 |
| Fam187a | Fam187a family with sequence similarity 187, member A | 0.22 | 0.23 | 1.23 | 0.52 | 1.06 | 5.22 |
| Gbp6 | Gbp6 guanylate binding protein 6 | 1.19 | 2.61 | 13.62 | 15.77 | 2.19 | 5.22 |
| Zp2 | Zp2 zona pellucida glycoprotein 2 | 0.44 | 0.37 | 1.89 | 2.62 | 0.82 | 5.18 |
| Hsf5 | Hsf5 heat shock transcription factor family member 5 | 0.17 | 0.21 | 1.07 | 0.60 | 1.26 | 5.14 |
| Cytl1 | Cytl1 cytokine-like 1 | 1.44 | 0.10 | 0.54 | 0.52 | 0.07 | 5.14 |
| Meioc | Meioc meiosis specific with coiled-coil domain | 0.86 | 0.86 | 4.40 | 2.85 | 1.00 | 5.11 |
| Pabpc1l | Pabpc1l poly(A) binding protein, cytoplasmic 1-like | 0.47 | 0.60 | 3.02 | 2.47 | 1.27 | 5.03 |
| Tex45 | Tex45 testis expressed 45 | 0.08 | 0.23 | 1.18 | 0.63 | 2.82 | 5.01 |
| Asgr1 | Asgr1 asialoglycoprotein receptor 1 | 1.58 | 0.47 | 2.35 | 1.35 | 0.30 | 5.01 |
| Eif2s3y | Eif2s3y eukaryotic translation initiation factor 2, subunit 3, structural gene Y-linked | 9.64 | 7.44 | 37.08 | 12.40 | 0.77 | 4.99 |
| Angpt2 | Angpt2 angiopoietin 2 | 1.36 | 1.07 | 5.24 | 6.30 | 0.79 | 4.90 |
| Mir26a-1 | Mir26a-1 microRNA 26a-1 | 0.08 | 0.13 | 0.64 | 0.23 | 1.57 | 4.90 |
| 1700008K24Rik | 1700008K24Rik RIKEN cDNA 1700008K24 gene | 0.08 | 0.05 | 0.26 | 0.09 | 0.63 | 4.90 |
| Nxnl2 | Nxnl2 nucleoredoxin-like 2 | 0.03 | 0.05 | 0.26 | 0.12 | 1.88 | 4.90 |
| 5730422E09Rik | 5730422E09Rik RIKEN cDNA 5730422E09 gene | 0.17 | 0.05 | 0.26 | 0.14 | 0.31 | 4.90 |
| Nox4 | Nox4 NADPH oxidase 4 | 0.08 | 0.05 | 0.26 | 0.17 | 0.63 | 4.90 |
| Fbxw7as1 | Fbxw7as1 F-box and WD-40 domain protein 7 antisense transcript 1 | 0.08 | 0.08 | 0.38 | 0.17 | 0.94 | 4.90 |
| Actg2 | Actg2 actin, gamma 2, smooth muscle, enteric | 0.17 | 0.05 | 0.26 | 0.17 | 0.31 | 4.90 |
| Ccnb1 | Ccnb1 cyclin B1 | 0.22 | 0.05 | 0.26 | 0.32 | 0.24 | 4.90 |
| F830016B08Rik | F830016B08Rik RIKEN cDNA F830016B08 gene | 0.03 | 0.08 | 0.38 | 0.37 | 2.82 | 4.90 |
| Il17f | Il17f interleukin 17F | 0.03 | 0.03 | 0.13 | 0.03 | 0.94 | 4.90 |
| Il22ra1 | Il22ra1 interleukin 22 receptor, alpha 1 | 0.03 | 0.03 | 0.13 | 0.03 | 0.94 | 4.90 |
| Cd300e | Cd300e CD300E molecule | 0.03 | 0.03 | 0.13 | 0.03 | 0.94 | 4.90 |
| Gm12596 | Gm12596 predicted gene 12596 | 0.06 | 0.03 | 0.13 | 0.03 | 0.47 | 4.90 |
| Acsm4 | Acsm4 acyl-CoA synthetase medium-chain family member 4 | 0.06 | 0.03 | 0.13 | 0.03 | 0.47 | 4.90 |
| Macc1 | Macc1 metastasis associated in colon cancer 1 | 0.06 | 0.03 | 0.13 | 0.03 | 0.47 | 4.90 |
| Tlr8 | Tlr8 toll-like receptor 8 | 0.08 | 0.03 | 0.13 | 0.03 | 0.31 | 4.90 |
| Stap1 | Stap1 signal transducing adaptor family member 1 | 0.03 | 0.03 | 0.13 | 0.06 | 0.94 | 4.90 |
| Fasl | Fasl Fas ligand (TNF superfamily, member 6) | 0.06 | 0.03 | 0.13 | 0.06 | 0.47 | 4.90 |
| Hsd3b6 | Hsd3b6 hydroxy-delta-5-steroid dehydrogenase, 3 beta- and steroid delta-isomerase 6 | 0.06 | 0.03 | 0.13 | 0.06 | 0.47 | 4.90 |
| Gm15350 | Gm15350 predicted gene 15350 | 0.06 | 0.03 | 0.13 | 0.06 | 0.47 | 4.90 |
| Tdg | Tdg thymine DNA glycosylase | 0.08 | 0.03 | 0.13 | 0.06 | 0.31 | 4.90 |
| Tssk2 | Tssk2 testis-specific serine kinase 2 | 0.08 | 0.03 | 0.13 | 0.06 | 0.31 | 4.90 |
| Nlrp4f | Nlrp4f NLR family, pyrin domain containing 4F | 0.14 | 0.03 | 0.13 | 0.06 | 0.19 | 4.90 |
| Klhl30 | Klhl30 kelch-like 30 | 0.19 | 0.03 | 0.13 | 0.06 | 0.13 | 4.90 |
| Gm4432 | Gm4432 predicted gene 4432 | 0.06 | 0.03 | 0.13 | 0.09 | 0.47 | 4.90 |
| Prss41 | Prss41 protease, serine 41 | 0.14 | 0.03 | 0.13 | 0.09 | 0.19 | 4.90 |
| Mir8112 | Mir8112 microRNA 8112 | 0.25 | 0.03 | 0.13 | 0.09 | 0.10 | 4.90 |
| Tyrp1 | Tyrp1 tyrosinase-related protein 1 | 0.28 | 0.03 | 0.13 | 0.09 | 0.09 | 4.90 |
| Barhl1 | Barhl1 BarH like homeobox 1 | 0.03 | 0.03 | 0.13 | 0.12 | 0.94 | 4.90 |
| BC048644 | BC048644 cDNA sequence BC048644 | 0.14 | 0.03 | 0.13 | 0.12 | 0.19 | 4.90 |
| D030045P18Rik | D030045P18Rik RIKEN cDNA D030045P18 gene | 0.08 | 0.03 | 0.13 | 0.14 | 0.31 | 4.90 |
| Gckr | Gckr glucokinase regulatory protein | 0.14 | 0.03 | 0.13 | 0.14 | 0.19 | 4.90 |
| Cilp | Cilp cartilage intermediate layer protein, nucleotide pyrophosphohydrolase | 0.14 | 0.03 | 0.13 | 0.17 | 0.19 | 4.90 |
| Fcrl1 | Fcrl1 Fc receptor-like 1 | 0.36 | 0.03 | 0.13 | 0.17 | 0.07 | 4.90 |
| Slc6a12 | Slc6a12 solute carrier family 6 (neurotransmitter transporter, betaine/GABA), member 12 | 1.88 | 0.91 | 4.45 | 5.64 | 0.48 | 4.87 |
| Pmaip1 | Pmaip1 phorbol-12-myristate-13-acetate-induced protein 1 | 0.78 | 2.09 | 10.12 | 8.20 | 2.69 | 4.85 |
| Slc27a5 | Slc27a5 solute carrier family 27 (fatty acid transporter), member 5 | 0.30 | 0.31 | 1.51 | 1.06 | 1.03 | 4.82 |
| Sult1c2 | Sult1c2 sulfotransferase family, cytosolic, 1C, member 2 | 0.42 | 0.18 | 0.87 | 0.69 | 0.44 | 4.76 |
| Fgf17 | Fgf17 fibroblast growth factor 17 | 0.19 | 0.16 | 0.74 | 0.32 | 0.81 | 4.73 |
| Emp1 | Emp1 epithelial membrane protein 1 | 2.36 | 4.88 | 22.92 | 20.45 | 2.07 | 4.70 |
| Nkx6-1 | Nkx6-1 NK6 homeobox 1 | 0.19 | 0.10 | 0.49 | 0.26 | 0.54 | 4.65 |
| Dio2 | Dio2 deiodinase, iodothyronine, type II | 35.86 | 46.73 | 217.26 | 196.00 | 1.30 | 4.65 |
| Iigp1 | Iigp1 interferon inducible GTPase 1 | 2.08 | 4.10 | 18.96 | 21.12 | 1.97 | 4.63 |
| Sh2d4b | Sh2d4b SH2 domain containing 4B | 0.14 | 0.18 | 0.84 | 0.55 | 1.32 | 4.62 |
| Zfp819 | Zfp819 zinc finger protein 819 | 0.03 | 0.08 | 0.36 | 0.14 | 2.82 | 4.57 |
| Hist1h2ac | Hist1h2ac histone cluster 1, H2ac | 0.17 | 0.08 | 0.36 | 0.17 | 0.47 | 4.57 |
| Scarna6 | Scarna6 small Cajal body-specific RNA 6 | 0.22 | 0.08 | 0.36 | 0.20 | 0.35 | 4.57 |
| Dlgap5 | Dlgap5 DLG associated protein 5 | 0.08 | 0.08 | 0.36 | 0.26 | 0.94 | 4.57 |
| E130310I04Rik | E130310I04Rik RIKEN cDNA E130310I04 gene | 0.14 | 0.08 | 0.36 | 0.32 | 0.56 | 4.57 |
| Il6 | Il6 interleukin 6 | 0.08 | 0.08 | 0.36 | 0.35 | 0.94 | 4.57 |
| Npm2 | Npm2 nucleophosmin/nucleoplasmin 2 | 0.28 | 0.23 | 1.07 | 0.69 | 0.85 | 4.57 |
| Fam24b | Fam24b family with sequence similarity 24 member B | 0.22 | 0.29 | 1.30 | 0.72 | 1.29 | 4.54 |
| Nanp | Nanp N-acetylneuraminic acid phosphatase | 0.94 | 0.42 | 1.89 | 0.98 | 0.44 | 4.53 |
| Scara5 | Scara5 scavenger receptor class A, member 5 | 1.83 | 0.94 | 4.24 | 4.69 | 0.51 | 4.52 |
| Cabp4 | Cabp4 calcium binding protein 4 | 0.58 | 0.60 | 2.71 | 1.15 | 1.03 | 4.51 |
| C4bp-ps1 | C4bp-ps1 complement component 4 binding protein, pseudogene 1 | 0.06 | 0.13 | 0.59 | 0.32 | 2.35 | 4.51 |
| Knl1 | Knl1 kinetochore scaffold 1 | 0.19 | 0.13 | 0.59 | 0.35 | 0.67 | 4.51 |
| Stc1 | Stc1 stanniocalcin 1 | 3.88 | 4.44 | 19.86 | 8.86 | 1.14 | 4.48 |
| Cavin4 | Cavin4 caveolae associated 4 | 0.55 | 0.42 | 1.87 | 1.04 | 0.75 | 4.47 |
| Apold1 | Apold1 apolipoprotein L domain containing 1 | 3.57 | 6.76 | 29.98 | 17.46 | 1.89 | 4.44 |
| Tc2n | Tc2n tandem C2 domains, nuclear | 0.80 | 0.50 | 2.20 | 1.01 | 0.62 | 4.43 |
| Trim72 | Trim72 tripartite motif-containing 72 | 0.19 | 1.20 | 5.32 | 5.84 | 6.19 | 4.43 |
| Syce1l | Syce1l synaptonemal complex central element protein 1 like | 0.08 | 0.10 | 0.46 | 0.40 | 1.26 | 4.41 |
| Adam21 | Adam21 a disintegrin and metallopeptidase domain 21 | 0.61 | 0.31 | 1.38 | 0.72 | 0.51 | 4.41 |
| Tldc2 | Tldc2 TBC/LysM associated domain containing 2 | 0.06 | 0.05 | 0.23 | 0.06 | 0.94 | 4.41 |
| Mir3100 | Mir3100 microRNA 3100 | 0.06 | 0.05 | 0.23 | 0.06 | 0.94 | 4.41 |
| Lmod3 | Lmod3 leiomodin 3 (fetal) | 0.03 | 0.05 | 0.23 | 0.09 | 1.88 | 4.41 |
| Mir3471-1 | Mir3471-1 microRNA 3471-1 | 0.03 | 0.05 | 0.23 | 0.12 | 1.88 | 4.41 |
| Mbnl3 | Mbnl3 muscleblind like splicing factor 3 | 0.17 | 0.05 | 0.23 | 0.12 | 0.31 | 4.41 |
| Mat1a | Mat1a methionine adenosyltransferase I, alpha | 0.19 | 0.05 | 0.23 | 0.23 | 0.27 | 4.41 |
| Cblc | Cblc Casitas B-lineage lymphoma c | 0.11 | 0.05 | 0.23 | 0.32 | 0.47 | 4.41 |
| Spata31d1a | Spata31d1a spermatogenesis associated 31 subfamily D, member 1A | 0.06 | 0.05 | 0.23 | 0.49 | 0.94 | 4.41 |
| 4930552P12Rik | 4930552P12Rik RIKEN cDNA 4930552P12 gene | 0.14 | 0.16 | 0.69 | 0.55 | 1.13 | 4.41 |
| Ager | Ager advanced glycosylation end product-specific receptor | 0.94 | 1.10 | 4.80 | 2.76 | 1.16 | 4.38 |
| Osmr | Osmr oncostatin M receptor | 3.69 | 10.98 | 47.48 | 34.41 | 2.98 | 4.32 |
| Glod5 | Glod5 glyoxalase domain containing 5 | 0.19 | 0.13 | 0.56 | 0.49 | 0.67 | 4.31 |
| Tbx15 | Tbx15 T-box 15 | 0.80 | 0.34 | 1.46 | 0.89 | 0.42 | 4.29 |
| 4930590J08Rik | 4930590J08Rik RIKEN cDNA 4930590J08 gene | 0.58 | 0.34 | 1.46 | 1.12 | 0.58 | 4.29 |
| Jhy | Jhy junctional cadherin complex regulator | 0.42 | 0.21 | 0.89 | 0.52 | 0.50 | 4.29 |
| Ins2 | Ins2 insulin II | 0.55 | 0.08 | 0.33 | 0.14 | 0.14 | 4.24 |
| 4930455C13Rik | 4930455C13Rik RIKEN cDNA 4930455C13 gene | 0.33 | 0.16 | 0.66 | 0.26 | 0.47 | 4.24 |
| AI839979 | AI839979 expressed sequence AI839979 | 0.36 | 0.50 | 2.10 | 1.09 | 1.38 | 4.23 |
| Chrna2 | Chrna2 cholinergic receptor, nicotinic, alpha polypeptide 2 (neuronal) | 1.22 | 1.25 | 5.26 | 3.42 | 1.03 | 4.20 |
| Rmrp | Rmrp RNA component of mitochondrial RNAase P | 0.33 | 0.18 | 0.77 | 0.40 | 0.55 | 4.20 |
| Muc2 | Muc2 mucin 2 | 0.42 | 0.47 | 1.97 | 2.19 | 1.13 | 4.19 |
| Depdc1b | Depdc1b DEP domain containing 1B | 0.25 | 0.10 | 0.43 | 0.12 | 0.42 | 4.16 |
| Avpr1a | Avpr1a arginine vasopressin receptor 1A | 0.19 | 0.10 | 0.43 | 0.55 | 0.54 | 4.16 |
| Gm15708 | Gm15708 predicted gene 15708 | 0.25 | 0.10 | 0.43 | 0.66 | 0.42 | 4.16 |
| Sct | Sct secretin | 0.08 | 0.10 | 0.43 | 1.06 | 1.26 | 4.16 |
| Muc1 | Muc1 mucin 1, transmembrane | 0.80 | 1.25 | 5.21 | 4.66 | 1.56 | 4.16 |
| Gfy | Gfy golgi-associated olfactory signaling regulator | 0.28 | 0.23 | 0.97 | 0.60 | 0.85 | 4.14 |
| Trp53inp1 | Trp53inp1 transformation related protein 53 inducible nuclear protein 1 | 12.55 | 11.43 | 47.05 | 32.31 | 0.91 | 4.12 |
| Pde6b | Pde6b phosphodiesterase 6B, cGMP, rod receptor, beta polypeptide | 0.19 | 0.29 | 1.18 | 1.09 | 1.48 | 4.10 |
| Fpr2 | Fpr2 formyl peptide receptor 2 | 0.03 | 0.29 | 1.18 | 1.35 | 10.36 | 4.10 |
| Pcp2 | Pcp2 Purkinje cell protein 2 (L7) | 0.67 | 0.16 | 0.64 | 0.72 | 0.24 | 4.08 |
| Erdr1 | Erdr1 erythroid differentiation regulator 1 | 7.09 | 4.85 | 19.70 | 5.49 | 0.68 | 4.06 |
| Cdh3 | Cdh3 cadherin 3 | 0.86 | 0.39 | 1.58 | 0.37 | 0.46 | 4.05 |
| Cd274 | Cd274 CD274 antigen | 2.16 | 4.12 | 16.48 | 22.90 | 1.91 | 4.00 |
| Gm16853 | Gm16853 predicted gene, 16853 | 0.42 | 0.37 | 1.46 | 0.49 | 0.88 | 3.99 |
| **Thbs1** | **Thbs1 thrombospondin 1** | **3.46** | **2.32** | **9.20** | **8.57** | **0.67** | **3.96** |
| Cd33 | Cd33 CD33 antigen | 3.99 | 3.55 | 14.03 | 11.71 | 0.89 | 3.95 |
| Ptx3 | Ptx3 pentraxin related gene | 0.44 | 1.12 | 4.42 | 3.34 | 2.53 | 3.94 |
| Galnt15 | Galnt15 polypeptide N-acetylgalactosaminyltransferase 15 | 1.61 | 2.77 | 10.89 | 11.33 | 1.72 | 3.94 |
| Trcg1 | Trcg1 taste receptor cell gene 1 | 0.08 | 0.10 | 0.41 | 0.32 | 1.26 | 3.92 |
| 9230116N13Rik | 9230116N13Rik RIKEN cDNA 9230116N13 gene | 0.36 | 0.10 | 0.41 | 0.66 | 0.29 | 3.92 |
| 5830432E09Rik | 5830432E09Rik RIKEN cDNA 5830432E09 gene | 0.08 | 0.08 | 0.31 | 0.09 | 0.94 | 3.92 |
| Bub1 | Bub1 BUB1, mitotic checkpoint serine/threonine kinase | 0.11 | 0.08 | 0.31 | 0.17 | 0.71 | 3.92 |
| Eid3 | Eid3 EP300 interacting inhibitor of differentiation 3 | 0.11 | 0.08 | 0.31 | 0.17 | 0.71 | 3.92 |
| Prdm12 | Prdm12 PR domain containing 12 | 0.14 | 0.08 | 0.31 | 0.32 | 0.56 | 3.92 |
| Fam166a | Fam166a family with sequence similarity 166, member A | 0.19 | 0.08 | 0.31 | 0.46 | 0.40 | 3.92 |
| 5730403I07Rik | 5730403I07Rik RIKEN cDNA 5730403I07 gene | 0.03 | 0.03 | 0.10 | 0.03 | 0.94 | 3.92 |
| Pitx1 | Pitx1 paired-like homeodomain transcription factor 1 | 0.03 | 0.03 | 0.10 | 0.03 | 0.94 | 3.92 |
| Bicdl2 | Bicdl2 BICD family like cargo adaptor 2 | 0.03 | 0.03 | 0.10 | 0.03 | 0.94 | 3.92 |
| Mir145a | Mir145a microRNA 145a | 0.03 | 0.03 | 0.10 | 0.03 | 0.94 | 3.92 |
| Bpifb5 | Bpifb5 BPI fold containing family B, member 5 | 0.06 | 0.03 | 0.10 | 0.03 | 0.47 | 3.92 |
| Glyatl3 | Glyatl3 glycine-N-acyltransferase-like 3 | 0.06 | 0.03 | 0.10 | 0.03 | 0.47 | 3.92 |
| Hamp2 | Hamp2 hepcidin antimicrobial peptide 2 | 0.11 | 0.03 | 0.10 | 0.03 | 0.24 | 3.92 |
| Vmo1 | Vmo1 vitelline membrane outer layer 1 homolog (chicken) | 0.14 | 0.03 | 0.10 | 0.03 | 0.19 | 3.92 |
| Gsdma | Gsdma gasdermin A | 0.03 | 0.05 | 0.20 | 0.06 | 1.88 | 3.92 |
| Gata3 | Gata3 GATA binding protein 3 | 0.06 | 0.03 | 0.10 | 0.06 | 0.47 | 3.92 |
| 1700018B24Rik | 1700018B24Rik RIKEN cDNA 1700018B24 gene | 0.06 | 0.03 | 0.10 | 0.06 | 0.47 | 3.92 |
| Ucn2 | Ucn2 urocortin 2 | 0.06 | 0.03 | 0.10 | 0.06 | 0.47 | 3.92 |
| Kng2 | Kng2 kininogen 2 | 0.06 | 0.03 | 0.10 | 0.06 | 0.47 | 3.92 |
| Mir7044 | Mir7044 microRNA 7044 | 0.08 | 0.05 | 0.20 | 0.06 | 0.63 | 3.92 |
| Dlx4 | Dlx4 distal-less homeobox 4 | 0.11 | 0.03 | 0.10 | 0.06 | 0.24 | 3.92 |
| Xlr4a | Xlr4a X-linked lymphocyte-regulated 4A | 0.25 | 0.03 | 0.10 | 0.06 | 0.10 | 3.92 |
| 9530091C08Rik | 9530091C08Rik RIKEN cDNA 9530091C08 gene | 0.06 | 0.03 | 0.10 | 0.09 | 0.47 | 3.92 |
| Igf2bp1 | Igf2bp1 insulin-like growth factor 2 mRNA binding protein 1 | 0.06 | 0.05 | 0.20 | 0.09 | 0.94 | 3.92 |
| Il20ra | Il20ra interleukin 20 receptor, alpha | 0.11 | 0.03 | 0.10 | 0.09 | 0.24 | 3.92 |
| D6Ertd527e | D6Ertd527e DNA segment, Chr 6, ERATO Doi 527, expressed | 0.14 | 0.05 | 0.20 | 0.09 | 0.38 | 3.92 |
| Gm6377 | Gm6377 predicted gene 6377 | 0.08 | 0.03 | 0.10 | 0.12 | 0.31 | 3.92 |
| BB031773 | BB031773 expressed sequence BB031773 | 0.17 | 0.03 | 0.10 | 0.12 | 0.16 | 3.92 |
| Art5 | Art5 ADP-ribosyltransferase 5 | 0.03 | 0.03 | 0.10 | 0.14 | 0.94 | 3.92 |
| Nxf7 | Nxf7 nuclear RNA export factor 7 | 0.03 | 0.03 | 0.10 | 0.14 | 0.94 | 3.92 |
| Mgarp | Mgarp mitochondria localized glutamic acid rich protein | 0.06 | 0.03 | 0.10 | 0.14 | 0.47 | 3.92 |
| Rbp2 | Rbp2 retinol binding protein 2, cellular | 0.06 | 0.05 | 0.20 | 0.14 | 0.94 | 3.92 |
| Gbp8 | Gbp8 guanylate-binding protein 8 | 0.19 | 0.05 | 0.20 | 0.14 | 0.27 | 3.92 |
| Fxyd4 | Fxyd4 FXYD domain-containing ion transport regulator 4 | 0.28 | 0.03 | 0.10 | 0.14 | 0.09 | 3.92 |
| Mir3470a | Mir3470a microRNA 3470a | 0.03 | 0.05 | 0.20 | 0.17 | 1.88 | 3.92 |
| Mfsd6l | Mfsd6l major facilitator superfamily domain containing 6-like | 0.06 | 0.05 | 0.20 | 0.17 | 0.94 | 3.92 |
| Otop1 | Otop1 otopetrin 1 | 0.03 | 0.05 | 0.20 | 0.20 | 1.88 | 3.92 |
| Fgf3 | Fgf3 fibroblast growth factor 3 | 0.06 | 0.03 | 0.10 | 0.20 | 0.47 | 3.92 |
| Padi6 | Padi6 peptidyl arginine deiminase, type VI | 0.08 | 0.03 | 0.10 | 0.20 | 0.31 | 3.92 |
| Snord83b | Snord83b small nucleolar RNA, C/D box 83B | 0.17 | 0.03 | 0.10 | 0.20 | 0.16 | 3.92 |
| Esco2 | Esco2 establishment of sister chromatid cohesion N-acetyltransferase 2 | 0.22 | 0.05 | 0.20 | 0.20 | 0.24 | 3.92 |
| Tbc1d21 | Tbc1d21 TBC1 domain family, member 21 | 0.06 | 0.03 | 0.10 | 0.26 | 0.47 | 3.92 |
| Exo1 | Exo1 exonuclease 1 | 0.39 | 0.26 | 1.02 | 1.12 | 0.67 | 3.92 |
| D630039A03Rik | D630039A03Rik RIKEN cDNA D630039A03 gene | 0.50 | 0.18 | 0.72 | 0.40 | 0.37 | 3.92 |
| Tmprss2 | Tmprss2 transmembrane protease, serine 2 | 0.08 | 0.16 | 0.61 | 0.72 | 1.88 | 3.92 |
| Ctla2a | Ctla2a cytotoxic T lymphocyte-associated protein 2 alpha | 2.99 | 29.74 | 116.36 | 88.44 | 9.94 | 3.91 |
| Gm20219 | Gm20219 predicted gene, 20219 | 1.83 | 1.59 | 6.21 | 2.99 | 0.87 | 3.90 |
| Cxcl10 | Cxcl10 chemokine (C-X-C motif) ligand 10 | 0.33 | 7.78 | 29.98 | 23.85 | 23.38 | 3.86 |
| H2-Aa | H2-Aa histocompatibility 2, class II antigen A, alpha | 2.88 | 0.39 | 1.51 | 1.47 | 0.14 | 3.85 |
| Tmem210 | Tmem210 transmembrane protein 210 | 0.75 | 0.86 | 3.30 | 2.82 | 1.15 | 3.83 |
| Cd300lf | Cd300lf CD300 molecule like family member F | 0.08 | 1.44 | 5.49 | 6.90 | 17.26 | 3.83 |
| Snord69 | Snord69 small nucleolar RNA, C/D box 69 | 0.47 | 0.70 | 2.68 | 1.47 | 1.50 | 3.81 |
| Ceacam16 | Ceacam16 carcinoembryonic antigen-related cell adhesion molecule 16 | 0.75 | 0.23 | 0.89 | 0.43 | 0.31 | 3.81 |
| Fmo2 | Fmo2 flavin containing monooxygenase 2 | 1.75 | 0.60 | 2.27 | 2.16 | 0.34 | 3.79 |
| 4930474N09Rik | 4930474N09Rik RIKEN cDNA 4930474N09 gene | 0.14 | 0.18 | 0.69 | 0.43 | 1.32 | 3.78 |
| Acod1 | Acod1 aconitate decarboxylase 1 | 0.03 | 0.78 | 2.94 | 3.68 | 28.25 | 3.75 |
| Ppp1r1c | Ppp1r1c protein phosphatase 1, regulatory inhibitor subunit 1C | 0.11 | 0.16 | 0.59 | 0.58 | 1.41 | 3.75 |
| Slc25a13 | Slc25a13 solute carrier family 25 (mitochondrial carrier, adenine nucleotide translocator), member 13 | 1.91 | 1.85 | 6.90 | 6.16 | 0.97 | 3.72 |
| Apobec2 | Apobec2 apolipoprotein B mRNA editing enzyme, catalytic polypeptide 2 | 0.25 | 0.13 | 0.49 | 0.17 | 0.52 | 3.72 |
| Olfr920 | Olfr920 olfactory receptor 920 | 0.22 | 0.26 | 0.97 | 0.83 | 1.18 | 3.72 |
| Mybph | Mybph myosin binding protein H | 0.25 | 0.34 | 1.25 | 0.83 | 1.36 | 3.69 |
| Il18rap | Il18rap interleukin 18 receptor accessory protein | 0.03 | 0.42 | 1.53 | 1.27 | 15.06 | 3.67 |
| Cfap161 | Cfap161 cilia and flagella associated protein 161 | 1.11 | 0.21 | 0.77 | 0.35 | 0.19 | 3.67 |
| L3mbtl4 | L3mbtl4 L3MBTL4 histone methyl-lysine binding protein | 0.11 | 0.08 | 0.28 | 0.09 | 0.71 | 3.59 |
| 4631405J19Rik | 4631405J19Rik RIKEN cDNA 4631405J19 gene | 0.14 | 0.08 | 0.28 | 0.12 | 0.56 | 3.59 |
| 4930447K03Rik | 4930447K03Rik RIKEN cDNA 4930447K03 gene | 0.17 | 0.08 | 0.28 | 0.12 | 0.47 | 3.59 |
| Uba1y | Uba1y ubiquitin-activating enzyme, Chr Y | 0.08 | 0.08 | 0.28 | 0.14 | 0.94 | 3.59 |
| 4930444M15Rik | 4930444M15Rik RIKEN cDNA 4930444M15 gene | 0.03 | 0.08 | 0.28 | 0.35 | 2.82 | 3.59 |
| Lmx1a | Lmx1a LIM homeobox transcription factor 1 alpha | 1.08 | 0.23 | 0.84 | 0.43 | 0.22 | 3.59 |
| Omd | Omd osteomodulin | 0.53 | 0.16 | 0.56 | 0.40 | 0.30 | 3.59 |
| Nt5e | Nt5e 5' nucleotidase, ecto | 3.49 | 3.81 | 13.62 | 8.40 | 1.09 | 3.58 |
| Kcne4 | Kcne4 potassium voltage-gated channel, Isk-related subfamily, gene 4 | 1.61 | 0.68 | 2.40 | 1.61 | 0.42 | 3.54 |
| Gbp10 | Gbp10 guanylate-binding protein 10 | 0.14 | 0.34 | 1.20 | 1.64 | 2.45 | 3.54 |
| Poln | Poln DNA polymerase N | 0.42 | 0.47 | 1.66 | 1.38 | 1.13 | 3.54 |
| Gnmt | Gnmt glycine N-methyltransferase | 1.39 | 1.80 | 6.36 | 5.70 | 1.30 | 3.53 |
| Dennd3 | Dennd3 DENN/MADD domain containing 3 | 8.29 | 14.04 | 49.55 | 32.05 | 1.69 | 3.53 |
| Hmgb2 | Hmgb2 high mobility group box 2 | 1.75 | 2.56 | 9.02 | 4.75 | 1.46 | 3.53 |
| 6330410L21Rik | 6330410L21Rik RIKEN cDNA 6330410L21 gene | 0.25 | 0.13 | 0.46 | 0.26 | 0.52 | 3.53 |
| Slc18a1 | Slc18a1 solute carrier family 18 (vesicular monoamine), member 1 | 0.28 | 0.13 | 0.46 | 0.26 | 0.47 | 3.53 |
| Ddias | Ddias DNA damage-induced apoptosis suppressor | 0.55 | 0.26 | 0.92 | 1.12 | 0.47 | 3.53 |
| Emilin3 | Emilin3 elastin microfibril interfacer 3 | 0.33 | 0.18 | 0.64 | 0.37 | 0.55 | 3.50 |
| H2-Bl | H2-Bl histocompatibility 2, blastocyst | 0.08 | 0.18 | 0.64 | 0.43 | 2.20 | 3.50 |
| H2-Q1 | H2-Q1 histocompatibility 2, Q region locus 1 | 0.50 | 0.18 | 0.64 | 0.58 | 0.37 | 3.50 |
| A3galt2 | A3galt2 alpha 1,3-galactosyltransferase 2 (isoglobotriaosylceramide synthase) | 0.36 | 0.18 | 0.64 | 0.72 | 0.51 | 3.50 |
| Steap1 | Steap1 six transmembrane epithelial antigen of the prostate 1 | 3.69 | 0.83 | 2.91 | 0.86 | 0.23 | 3.49 |
| Fzd4 | Fzd4 frizzled class receptor 4 | 8.92 | 7.31 | 25.45 | 14.76 | 0.82 | 3.48 |
| Fpr1 | Fpr1 formyl peptide receptor 1 | 0.14 | 0.44 | 1.53 | 1.32 | 3.20 | 3.46 |
| Gpr21 | Gpr21 G protein-coupled receptor 21 | 0.83 | 0.50 | 1.71 | 0.89 | 0.60 | 3.45 |
| Mst1 | Mst1 macrophage stimulating 1 (hepatocyte growth factor-like) | 0.44 | 0.31 | 1.07 | 1.12 | 0.71 | 3.43 |
| Cdkn3 | Cdkn3 cyclin-dependent kinase inhibitor 3 | 0.11 | 0.10 | 0.36 | 0.17 | 0.94 | 3.43 |
| Peg12 | Peg12 paternally expressed 12 | 0.14 | 0.10 | 0.36 | 0.29 | 0.75 | 3.43 |
| Gm15319 | Gm15319 predicted gene 15319 | 0.19 | 0.10 | 0.36 | 0.29 | 0.54 | 3.43 |
| 4930429F24Rik | 4930429F24Rik RIKEN cDNA 4930429F24 gene | 1.19 | 0.68 | 2.33 | 1.73 | 0.57 | 3.43 |
| Snora31 | Snora31 small nucleolar RNA, H/ACA box 31 | 0.33 | 0.16 | 0.54 | 0.35 | 0.47 | 3.43 |
| Hao2 | Hao2 hydroxyacid oxidase 2 | 0.06 | 0.05 | 0.18 | 0.06 | 0.94 | 3.43 |
| Gm5441 | Gm5441 predicted gene 5441 | 0.08 | 0.05 | 0.18 | 0.06 | 0.63 | 3.43 |
| Fgg | Fgg fibrinogen gamma chain | 0.19 | 0.05 | 0.18 | 0.12 | 0.27 | 3.43 |
| Lgals2 | Lgals2 lectin, galactose-binding, soluble 2 | 0.28 | 0.05 | 0.18 | 0.12 | 0.19 | 3.43 |
| Mir106b | Mir106b microRNA 106b | 0.06 | 0.05 | 0.18 | 0.17 | 0.94 | 3.43 |
| Acnat1 | Acnat1 acyl-coenzyme A amino acid N-acyltransferase 1 | 0.75 | 0.05 | 0.18 | 0.17 | 0.07 | 3.43 |
| Gm2093 | Gm2093 predicted gene 2093 | 0.03 | 0.05 | 0.18 | 0.20 | 1.88 | 3.43 |
| Ccdc150 | Ccdc150 coiled-coil domain containing 150 | 0.19 | 0.05 | 0.18 | 0.20 | 0.27 | 3.43 |
| Tmem174 | Tmem174 transmembrane protein 174 | 0.25 | 0.05 | 0.18 | 0.23 | 0.21 | 3.43 |
| Snord57 | Snord57 small nucleolar RNA, C/D box 57 | 0.11 | 0.05 | 0.18 | 0.26 | 0.47 | 3.43 |
| Tnfrsf4 | Tnfrsf4 tumor necrosis factor receptor superfamily, member 4 | 1.27 | 0.86 | 2.94 | 1.87 | 0.68 | 3.41 |
| Selp | Selp selectin, platelet | 0.03 | 0.65 | 2.22 | 4.83 | 23.54 | 3.41 |
| Lif | Lif leukemia inhibitory factor | 1.00 | 2.04 | 6.93 | 2.93 | 2.04 | 3.40 |
| Flnc | Flnc filamin C, gamma | 3.71 | 5.66 | 19.19 | 18.56 | 1.52 | 3.39 |
| Spdef | Spdef SAM pointed domain containing ets transcription factor | 0.22 | 1.64 | 5.57 | 5.61 | 7.41 | 3.39 |
| Adamts1 | Adamts1 a disintegrin-like and metallopeptidase (reprolysin type) with thrombospondin type 1 motif, 1 | 18.90 | 40.73 | 138.00 | 90.39 | 2.15 | 3.39 |
| Irgm1 | Irgm1 immunity-related GTPase family M member 1 | 6.65 | 17.59 | 59.37 | 55.96 | 2.64 | 3.38 |
| Grhl2 | Grhl2 grainyhead like transcription factor 2 | 0.28 | 0.23 | 0.79 | 0.37 | 0.85 | 3.37 |
| 1300017J02Rik | 1300017J02Rik RIKEN cDNA 1300017J02 gene | 0.14 | 0.23 | 0.79 | 1.09 | 1.69 | 3.37 |
| Duox1 | Duox1 dual oxidase 1 | 0.39 | 0.18 | 0.61 | 0.69 | 0.47 | 3.36 |
| Ticrr | Ticrr TOPBP1-interacting checkpoint and replication regulator | 0.19 | 0.13 | 0.43 | 0.23 | 0.67 | 3.33 |
| Fgfr4 | Fgfr4 fibroblast growth factor receptor 4 | 0.50 | 0.13 | 0.43 | 0.37 | 0.26 | 3.33 |
| Snora41 | Snora41 small nucleolar RNA, H/ACA box 41 | 0.08 | 0.13 | 0.43 | 0.49 | 1.57 | 3.33 |
| 4930562C15Rik | 4930562C15Rik RIKEN cDNA 4930562C15 gene | 0.14 | 0.26 | 0.87 | 0.43 | 1.88 | 3.33 |
| Gem | Gem GTP binding protein (gene overexpressed in skeletal muscle) | 1.25 | 1.38 | 4.60 | 2.50 | 1.11 | 3.33 |
| Atad5 | Atad5 ATPase family, AAA domain containing 5 | 4.21 | 4.15 | 13.67 | 10.01 | 0.98 | 3.30 |
| Efhc1 | Efhc1 EF-hand domain (C-terminal) containing 1 | 2.00 | 1.41 | 4.63 | 4.49 | 0.71 | 3.28 |
| Oacyl | Oacyl O-acyltransferase like | 1.22 | 0.97 | 3.17 | 2.47 | 0.79 | 3.28 |
| Fmod | Fmod fibromodulin | 21.09 | 3.84 | 12.57 | 12.14 | 0.18 | 3.28 |
| Snord2 | Snord2 small nucleolar RNA, C/D box 2 | 0.42 | 0.31 | 1.02 | 0.55 | 0.75 | 3.26 |
| Tmem150b | Tmem150b transmembrane protein 150B | 0.47 | 0.31 | 1.02 | 0.63 | 0.66 | 3.26 |
| Xlr3a | Xlr3a X-linked lymphocyte-regulated 3A | 0.61 | 0.08 | 0.26 | 0.06 | 0.13 | 3.26 |
| Gsc | Gsc goosecoid homeobox | 0.03 | 0.08 | 0.26 | 0.09 | 2.82 | 3.26 |
| Gm30505 | Gm30505 predicted gene, 30505 | 0.06 | 0.08 | 0.26 | 0.09 | 1.41 | 3.26 |
| Hist1h1e | Hist1h1e histone cluster 1, H1e | 0.39 | 0.08 | 0.26 | 0.09 | 0.20 | 3.26 |
| Il7r | Il7r interleukin 7 receptor | 0.30 | 0.08 | 0.26 | 0.23 | 0.26 | 3.26 |
| Dpt | Dpt dermatopontin | 0.14 | 0.08 | 0.26 | 0.29 | 0.56 | 3.26 |
| Chrne | Chrne cholinergic receptor, nicotinic, epsilon polypeptide | 0.14 | 0.08 | 0.26 | 0.29 | 0.56 | 3.26 |
| Rnf223 | Rnf223 ring finger 223 | 0.53 | 0.63 | 2.04 | 1.50 | 1.19 | 3.26 |
| Spem1 | Spem1 sperm maturation 1 | 0.17 | 0.16 | 0.51 | 0.23 | 0.94 | 3.26 |
| Nanos2 | Nanos2 nanos C2HC-type zinc finger 2 | 0.53 | 0.73 | 2.38 | 2.10 | 1.39 | 3.25 |
| Ccna2 | Ccna2 cyclin A2 | 1.41 | 1.02 | 3.30 | 2.59 | 0.72 | 3.24 |
| Muc6 | Muc6 mucin 6, gastric | 0.17 | 0.26 | 0.84 | 0.20 | 1.57 | 3.23 |
| E130112N10Rik | E130112N10Rik RIKEN cDNA E130112N10 gene | 0.67 | 0.55 | 1.76 | 1.12 | 0.82 | 3.22 |
| Lilr4b | Lilr4b leukocyte immunoglobulin-like receptor, subfamily B, member 4B | 0.08 | 0.18 | 0.59 | 0.75 | 2.20 | 3.22 |
| Svep1 | Svep1 sushi, von Willebrand factor type A, EGF and pentraxin domain containing 1 | 1.47 | 0.73 | 2.35 | 2.19 | 0.50 | 3.22 |
| Irgc1 | Irgc1 immunity-related GTPase family, cinema 1 | 0.03 | 0.10 | 0.33 | 0.14 | 3.77 | 3.18 |
| **Cdh1** | **Cdh1 cadherin 1** | **0.94** | **0.21** | **0.66** | **0.32** | **0.22** | **3.18** |
| Mir8091 | Mir8091 microRNA 8091 | 0.14 | 0.21 | 0.66 | 0.37 | 1.51 | 3.18 |
| Tcp10b | Tcp10b t-complex protein 10b | 0.03 | 0.10 | 0.33 | 0.46 | 3.77 | 3.18 |
| Mir3061 | Mir3061 microRNA 3061 | 0.78 | 1.04 | 3.32 | 2.45 | 1.35 | 3.18 |
| Wfikkn2 | Wfikkn2 WAP, follistatin/kazal, immunoglobulin, kunitz and netrin domain containing 2 | 4.68 | 4.62 | 14.69 | 9.03 | 0.99 | 3.18 |
| Gp1ba | Gp1ba glycoprotein 1b, alpha polypeptide | 0.80 | 0.55 | 1.74 | 1.29 | 0.68 | 3.17 |
| Irx3 | Irx3 Iroquois related homeobox 3 | 0.42 | 0.89 | 2.81 | 2.45 | 2.13 | 3.17 |
| Slfn4 | Slfn4 schlafen 4 | 0.03 | 0.91 | 2.89 | 3.05 | 32.95 | 3.16 |
| Fgf2 | Fgf2 fibroblast growth factor 2 | 0.50 | 0.57 | 1.81 | 1.24 | 1.15 | 3.16 |
| Prrg4 | Prrg4 proline rich Gla (G-carboxyglutamic acid) 4 (transmembrane) | 1.55 | 0.47 | 1.48 | 1.04 | 0.30 | 3.16 |
| Serpina3f | Serpina3f serine (or cysteine) peptidase inhibitor, clade A, member 3F | 0.03 | 1.20 | 3.78 | 6.73 | 43.31 | 3.15 |
| Mki67 | Mki67 antigen identified by monoclonal antibody Ki 67 | 2.19 | 1.28 | 4.01 | 2.50 | 0.58 | 3.14 |
| Arg1 | Arg1 arginase, liver | 0.14 | 0.13 | 0.41 | 0.14 | 0.94 | 3.13 |
| Gdf15 | Gdf15 growth differentiation factor 15 | 0.17 | 0.13 | 0.41 | 0.63 | 0.78 | 3.13 |
| 2700046A07Rik | 2700046A07Rik RIKEN cDNA 2700046A07 gene | 0.25 | 0.26 | 0.82 | 0.78 | 1.05 | 3.13 |
| Clcf1 | Clcf1 cardiotrophin-like cytokine factor 1 | 0.58 | 0.39 | 1.23 | 1.01 | 0.67 | 3.13 |
| Uty | Uty ubiquitously transcribed tetratricopeptide repeat gene, Y chromosome | 3.88 | 2.37 | 7.44 | 2.53 | 0.61 | 3.13 |
| Clcn1 | Clcn1 chloride channel, voltage-sensitive 1 | 2.24 | 1.85 | 5.80 | 4.57 | 0.83 | 3.13 |
| 4933433G19Rik | 4933433G19Rik RIKEN cDNA 4933433G19 gene | 1.14 | 0.86 | 2.68 | 1.67 | 0.76 | 3.12 |
| Mcoln2 | Mcoln2 mucolipin 2 | 0.06 | 0.16 | 0.49 | 1.04 | 2.82 | 3.10 |
| Onecut1 | Onecut1 one cut domain, family member 1 | 0.64 | 0.50 | 1.53 | 0.60 | 0.78 | 3.09 |
| BC018473 | BC018473 cDNA sequence BC018473 | 0.28 | 0.50 | 1.53 | 1.32 | 1.79 | 3.09 |
| Tgtp1 | Tgtp1 T cell specific GTPase 1 | 0.89 | 1.59 | 4.91 | 7.31 | 1.79 | 3.08 |
| Kcnrg | Kcnrg potassium channel regulator | 0.61 | 0.37 | 1.12 | 0.81 | 0.60 | 3.08 |
| Klhl10 | Klhl10 kelch-like 10 | 0.33 | 0.39 | 1.20 | 0.92 | 1.18 | 3.07 |
| Mpzl2 | Mpzl2 myelin protein zero-like 2 | 1.91 | 1.02 | 3.12 | 3.14 | 0.53 | 3.06 |
| St8sia3os | St8sia3os ST8 alpha-N-acetyl-neuraminide alpha-2,8-sialyltransferase 3, opposite strand | 0.83 | 0.63 | 1.92 | 1.73 | 0.75 | 3.06 |
| Klk7 | Klk7 kallikrein related-peptidase 7 (chymotryptic, stratum corneum) | 0.25 | 0.21 | 0.64 | 0.95 | 0.84 | 3.06 |
| Wdr63 | Wdr63 WD repeat domain 63 | 1.36 | 0.23 | 0.72 | 0.46 | 0.17 | 3.05 |
| A2m | A2m alpha-2-macroglobulin | 9.45 | 23.40 | 71.28 | 48.85 | 2.48 | 3.05 |
| Rell1 | Rell1 RELT-like 1 | 9.34 | 6.76 | 20.47 | 15.51 | 0.72 | 3.03 |
| Hrc | Hrc histidine rich calcium binding protein | 0.33 | 0.29 | 0.87 | 1.21 | 0.86 | 3.03 |
| Blm | Blm Bloom syndrome, RecQ like helicase | 2.94 | 2.56 | 7.67 | 6.88 | 0.87 | 3.00 |
| Ctgf | Ctgf connective tissue growth factor | 17.26 | 16.18 | 48.22 | 48.19 | 0.94 | 2.98 |
| H19 | H19 H19, imprinted maternally expressed transcript | 0.11 | 0.70 | 2.10 | 2.50 | 6.36 | 2.97 |
| Slc43a3 | Slc43a3 solute carrier family 43, member 3 | 1.25 | 1.57 | 4.65 | 3.77 | 1.26 | 2.97 |
| Slc4a11 | Slc4a11 solute carrier family 4, sodium bicarbonate transporter-like, member 11 | 0.94 | 0.94 | 2.79 | 2.30 | 1.00 | 2.97 |
| Cldn1 | Cldn1 claudin 1 | 4.99 | 2.97 | 8.79 | 5.67 | 0.60 | 2.96 |
| Lamc2 | Lamc2 laminin, gamma 2 | 4.35 | 1.98 | 5.85 | 6.10 | 0.46 | 2.95 |
| Rev1 | Rev1 REV1, DNA directed polymerase | 16.07 | 14.53 | 42.73 | 26.27 | 0.90 | 2.94 |
| Scpep1os | Scpep1os serine carboxypeptidase 1, opposite strand | 0.19 | 0.13 | 0.38 | 0.37 | 0.67 | 2.94 |
| 4933430I17Rik | 4933430I17Rik RIKEN cDNA 4933430I17 gene | 0.30 | 0.13 | 0.38 | 0.58 | 0.43 | 2.94 |
| Stx11 | Stx11 syntaxin 11 | 0.25 | 0.65 | 1.92 | 1.12 | 2.62 | 2.94 |
| Mir6998 | Mir6998 microRNA 6998 | 0.14 | 0.10 | 0.31 | 0.06 | 0.75 | 2.94 |
| Pdcd1lg2 | Pdcd1lg2 programmed cell death 1 ligand 2 | 0.06 | 0.10 | 0.31 | 0.12 | 1.88 | 2.94 |
| Calhm6 | Calhm6 calcium homeostasis modulator family member 6 | 0.14 | 0.10 | 0.31 | 0.23 | 0.75 | 2.94 |
| Galr2 | Galr2 galanin receptor 2 | 0.67 | 0.73 | 2.15 | 1.58 | 1.10 | 2.94 |
| Nexn | Nexn nexilin | 0.94 | 0.31 | 0.92 | 0.66 | 0.33 | 2.94 |
| Myzap | Myzap myocardial zonula adherens protein | 0.58 | 0.21 | 0.61 | 0.55 | 0.36 | 2.94 |
| Mir6915 | Mir6915 microRNA 6915 | 0.03 | 0.05 | 0.15 | 0.03 | 1.88 | 2.94 |
| Myl3 | Myl3 myosin, light polypeptide 3 | 0.08 | 0.05 | 0.15 | 0.03 | 0.63 | 2.94 |
| Runx3 | Runx3 runt related transcription factor 3 | 0.03 | 0.05 | 0.15 | 0.06 | 1.88 | 2.94 |
| Nxf2 | Nxf2 nuclear RNA export factor 2 | 0.03 | 0.05 | 0.15 | 0.12 | 1.88 | 2.94 |
| Mir8116 | Mir8116 microRNA 8116 | 0.06 | 0.05 | 0.15 | 0.12 | 0.94 | 2.94 |
| Prph2 | Prph2 peripherin 2 | 0.08 | 0.05 | 0.15 | 0.12 | 0.63 | 2.94 |
| Timd4 | Timd4 T cell immunoglobulin and mucin domain containing 4 | 0.03 | 0.05 | 0.15 | 0.14 | 1.88 | 2.94 |
| **Gdnf** | **Gdnf glial cell line derived neurotrophic factor** | **0.11** | **0.05** | **0.15** | **0.14** | **0.47** | **2.94** |
| Zfp820 | Zfp820 zinc finger protein 820 | 0.28 | 0.05 | 0.15 | 0.14 | 0.19 | 2.94 |
| Plg | Plg plasminogen | 0.03 | 0.05 | 0.15 | 0.17 | 1.88 | 2.94 |
| Col10a1 | Col10a1 collagen, type X, alpha 1 | 0.14 | 0.05 | 0.15 | 0.17 | 0.38 | 2.94 |
| Nuggc | Nuggc nuclear GTPase, germinal center associated | 0.14 | 0.05 | 0.15 | 0.17 | 0.38 | 2.94 |
| Snord15b | Snord15b small nucleolar RNA, C/D box 14B | 0.22 | 0.05 | 0.15 | 0.17 | 0.24 | 2.94 |
| 1700065J18Rik | 1700065J18Rik RIKEN cDNA 1700065J18 gene | 0.22 | 0.05 | 0.15 | 0.23 | 0.24 | 2.94 |
| Tat | Tat tyrosine aminotransferase | 0.19 | 0.05 | 0.15 | 0.35 | 0.27 | 2.94 |
| Defb11 | Defb11 defensin beta 11 | 0.64 | 0.18 | 0.54 | 0.29 | 0.29 | 2.94 |
| Myl1 | Myl1 myosin, light polypeptide 1 | 0.36 | 0.18 | 0.54 | 0.58 | 0.51 | 2.94 |
| Angptl7 | Angptl7 angiopoietin-like 7 | 0.17 | 0.18 | 0.54 | 0.66 | 1.10 | 2.94 |
| Myl7 | Myl7 myosin, light polypeptide 7, regulatory | 0.06 | 0.08 | 0.23 | 0.09 | 1.41 | 2.94 |
| Hsd17b14 | Hsd17b14 hydroxysteroid (17-beta) dehydrogenase 14 | 0.14 | 0.08 | 0.23 | 0.12 | 0.56 | 2.94 |
| 4930405A21Rik | 4930405A21Rik RIKEN cDNA 4930405A21 gene | 0.22 | 0.08 | 0.23 | 0.12 | 0.35 | 2.94 |
| Myh13 | Myh13 myosin, heavy polypeptide 13, skeletal muscle | 0.17 | 0.08 | 0.23 | 0.17 | 0.47 | 2.94 |
| Mir6907 | Mir6907 microRNA 6907 | 0.08 | 0.08 | 0.23 | 0.29 | 0.94 | 2.94 |
| Gm5936 | Gm5936 predicted gene 5936 | 0.22 | 0.08 | 0.23 | 0.32 | 0.35 | 2.94 |
| BC016579 | BC016579 cDNA sequence, BC016579 | 0.22 | 0.08 | 0.23 | 0.32 | 0.35 | 2.94 |
| Gm6260 | Gm6260 predicted gene 6260 | 0.03 | 0.03 | 0.08 | 0.03 | 0.94 | 2.94 |
| Dnajb8 | Dnajb8 DnaJ heat shock protein family (Hsp40) member B8 | 0.03 | 0.03 | 0.08 | 0.03 | 0.94 | 2.94 |
| 4930471M09Rik | 4930471M09Rik RIKEN cDNA 4930471M09 gene | 0.03 | 0.03 | 0.08 | 0.03 | 0.94 | 2.94 |
| Gpr141 | Gpr141 G protein-coupled receptor 141 | 0.03 | 0.03 | 0.08 | 0.03 | 0.94 | 2.94 |
| Npy4r | Npy4r neuropeptide Y receptor Y4 | 0.03 | 0.03 | 0.08 | 0.03 | 0.94 | 2.94 |
| 4930593A02Rik | 4930593A02Rik RIKEN cDNA 4930593A02 gene | 0.08 | 0.03 | 0.08 | 0.03 | 0.31 | 2.94 |
| Mybphl | Mybphl myosin binding protein H-like | 0.08 | 0.03 | 0.08 | 0.03 | 0.31 | 2.94 |
| 4833428L15Rik | 4833428L15Rik RIKEN cDNA 4833428L15 gene | 0.08 | 0.03 | 0.08 | 0.03 | 0.31 | 2.94 |
| Tcf21 | Tcf21 transcription factor 21 | 0.17 | 0.03 | 0.08 | 0.03 | 0.16 | 2.94 |
| Ccdc192 | Ccdc192 coiled-coil domain containing 192 | 0.17 | 0.03 | 0.08 | 0.03 | 0.16 | 2.94 |
| Sh2d1b1 | Sh2d1b1 SH2 domain containing 1B1 | 0.03 | 0.03 | 0.08 | 0.06 | 0.94 | 2.94 |
| Olfr70 | Olfr70 olfactory receptor 70 | 0.03 | 0.03 | 0.08 | 0.06 | 0.94 | 2.94 |
| Hoxa7 | Hoxa7 homeobox A7 | 0.03 | 0.03 | 0.08 | 0.06 | 0.94 | 2.94 |
| Gm38436 | Gm38436 predicted gene, 38436 | 0.03 | 0.03 | 0.08 | 0.06 | 0.94 | 2.94 |
| Myo7b | Myo7b myosin VIIB | 0.03 | 0.03 | 0.08 | 0.06 | 0.94 | 2.94 |
| Olfr544 | Olfr544 olfactory receptor 544 | 0.06 | 0.03 | 0.08 | 0.06 | 0.47 | 2.94 |
| Gm13986 | Gm13986 predicted gene 13986 | 0.08 | 0.03 | 0.08 | 0.06 | 0.31 | 2.94 |
| 2310001K24Rik | 2310001K24Rik RIKEN cDNA 2310001K24 gene | 0.08 | 0.03 | 0.08 | 0.06 | 0.31 | 2.94 |
| Alx1 | Alx1 ALX homeobox 1 | 0.08 | 0.03 | 0.08 | 0.06 | 0.31 | 2.94 |
| Slc5a8 | Slc5a8 solute carrier family 5 (iodide transporter), member 8 | 0.08 | 0.03 | 0.08 | 0.06 | 0.31 | 2.94 |
| NA | NA | 0.08 | 0.03 | 0.08 | 0.06 | 0.31 | 2.94 |
| Nccrp1 | Nccrp1 non-specific cytotoxic cell receptor protein 1 homolog (zebrafish) | 0.06 | 0.03 | 0.08 | 0.09 | 0.47 | 2.94 |
| Stpg2 | Stpg2 sperm tail PG rich repeat containing 2 | 0.08 | 0.03 | 0.08 | 0.09 | 0.31 | 2.94 |
| Fhl5 | Fhl5 four and a half LIM domains 5 | 0.08 | 0.03 | 0.08 | 0.09 | 0.31 | 2.94 |
| Foxl1 | Foxl1 forkhead box L1 | 0.08 | 0.03 | 0.08 | 0.09 | 0.31 | 2.94 |
| Snora15 | Snora15 small nucleolar RNA, H/ACA box 15 | 0.14 | 0.03 | 0.08 | 0.09 | 0.19 | 2.94 |
| I730030J21Rik | I730030J21Rik RIKEN cDNA I730030J21 gene | 0.25 | 0.03 | 0.08 | 0.09 | 0.10 | 2.94 |
| Ano9 | Ano9 anoctamin 9 | 0.06 | 0.03 | 0.08 | 0.12 | 0.47 | 2.94 |
| Best2 | Best2 bestrophin 2 | 0.06 | 0.03 | 0.08 | 0.12 | 0.47 | 2.94 |
| B020018J22Rik | B020018J22Rik Riken cDNA B020018J22 gene | 0.06 | 0.03 | 0.08 | 0.12 | 0.47 | 2.94 |
| Hist1h2bh | Hist1h2bh histone cluster 1, H2bh | 0.06 | 0.03 | 0.08 | 0.12 | 0.47 | 2.94 |
| Gm3500 | Gm3500 predicted gene 3500 | 0.06 | 0.03 | 0.08 | 0.12 | 0.47 | 2.94 |
| Gm6904 | Gm6904 predicted gene 6904 | 0.06 | 0.03 | 0.08 | 0.12 | 0.47 | 2.94 |
| Dscc1 | Dscc1 DNA replication and sister chromatid cohesion 1 | 0.06 | 0.03 | 0.08 | 0.12 | 0.47 | 2.94 |
| Gpr15 | Gpr15 G protein-coupled receptor 15 | 0.08 | 0.03 | 0.08 | 0.12 | 0.31 | 2.94 |
| Lexm | Lexm lymphocyte expansion molecule | 0.14 | 0.03 | 0.08 | 0.12 | 0.19 | 2.94 |
| Klkb1 | Klkb1 kallikrein B, plasma 1 | 0.03 | 0.03 | 0.08 | 0.17 | 0.94 | 2.94 |
| Pzp | Pzp PZP, alpha-2-macroglobulin like | 0.06 | 0.03 | 0.08 | 0.20 | 0.47 | 2.94 |
| Ms4a4d | Ms4a4d membrane-spanning 4-domains, subfamily A, member 4D | 0.08 | 0.03 | 0.08 | 0.26 | 0.31 | 2.94 |
| Cd300lg | Cd300lg CD300 molecule like family member G | 0.42 | 0.16 | 0.46 | 0.49 | 0.38 | 2.94 |
| P2rx1 | P2rx1 purinergic receptor P2X, ligand-gated ion channel, 1 | 1.03 | 0.26 | 0.77 | 0.63 | 0.25 | 2.94 |
| Scd4 | Scd4 stearoyl-coenzyme A desaturase 4 | 0.42 | 0.26 | 0.77 | 1.21 | 0.63 | 2.94 |
| Itgb1bp2 | Itgb1bp2 integrin beta 1 binding protein 2 | 0.39 | 0.55 | 1.61 | 1.18 | 1.41 | 2.94 |
| Arhgap20os | Arhgap20os Rho GTPase activating protein 20, opposite strand | 0.22 | 0.23 | 0.69 | 0.43 | 1.06 | 2.94 |
| Gbp2 | Gbp2 guanylate binding protein 2 | 4.07 | 11.56 | 33.89 | 54.83 | 2.84 | 2.93 |
| Crybg1 | Crybg1 crystallin beta-gamma domain containing 1 | 2.16 | 1.85 | 5.42 | 5.55 | 0.86 | 2.92 |
| P2rx6 | P2rx6 purinergic receptor P2X, ligand-gated ion channel, 6 | 8.23 | 7.07 | 20.57 | 22.96 | 0.86 | 2.91 |
| Rflna | Rflna refilin A | 0.83 | 0.81 | 2.35 | 0.95 | 0.97 | 2.91 |
| Lepr | Lepr leptin receptor | 3.21 | 1.59 | 4.63 | 4.17 | 0.50 | 2.91 |
| Stxbp3-ps | Stxbp3-ps syntaxin-binding protein 3, pseudogene | 1.05 | 0.70 | 2.04 | 1.75 | 0.67 | 2.90 |
| Tnk2os | Tnk2os tyrosine kinase, non-receptor 2, opposite strand | 0.64 | 0.60 | 1.74 | 1.01 | 0.94 | 2.90 |
| Capn6 | Capn6 calpain 6 | 1.44 | 0.50 | 1.43 | 1.15 | 0.34 | 2.89 |
| Smtnl2 | Smtnl2 smoothelin-like 2 | 2.83 | 3.10 | 8.94 | 11.39 | 1.10 | 2.88 |
| Acta2 | Acta2 actin, alpha 2, smooth muscle, aorta | 29.57 | 10.44 | 30.03 | 27.39 | 0.35 | 2.88 |
| Trim43a | Trim43a tripartite motif-containing 43A | 0.72 | 0.39 | 1.12 | 1.06 | 0.54 | 2.87 |
| Gbp3 | Gbp3 guanylate binding protein 3 | 3.13 | 8.64 | 24.76 | 24.65 | 2.76 | 2.87 |
| Tlr7 | Tlr7 toll-like receptor 7 | 2.11 | 4.23 | 12.11 | 9.32 | 2.01 | 2.87 |
| 4930549G23Rik | 4930549G23Rik RIKEN cDNA 4930549G23 gene | 0.47 | 0.31 | 0.89 | 0.35 | 0.66 | 2.86 |
| Eppk1 | Eppk1 epiplakin 1 | 0.14 | 0.29 | 0.82 | 0.60 | 2.07 | 2.85 |
| Mpp4 | Mpp4 membrane protein, palmitoylated 4 (MAGUK p55 subfamily member 4) | 0.58 | 0.29 | 0.82 | 0.60 | 0.49 | 2.85 |
| Snora28 | Snora28 small nucleolar RNA, H/ACA box 28 | 0.89 | 0.83 | 2.38 | 1.41 | 0.94 | 2.85 |
| Tgif1 | Tgif1 TGFB-induced factor homeobox 1 | 1.61 | 1.36 | 3.86 | 3.48 | 0.84 | 2.84 |
| Spc25 | Spc25 SPC25, NDC80 kinetochore complex component, homolog (S. cerevisiae) | 0.19 | 0.26 | 0.74 | 0.81 | 1.35 | 2.84 |
| Lum | Lum lumican | 4.57 | 1.80 | 5.11 | 3.37 | 0.39 | 2.84 |
| Aoc2 | Aoc2 amine oxidase, copper containing 2 (retina-specific) | 3.96 | 3.81 | 10.81 | 6.44 | 0.96 | 2.84 |
| Slc16a8 | Slc16a8 solute carrier family 16 (monocarboxylic acid transporters), member 8 | 8.34 | 2.27 | 6.44 | 3.62 | 0.27 | 2.84 |
| Oas3 | Oas3 2'-5' oligoadenylate synthetase 3 | 0.11 | 0.23 | 0.66 | 0.35 | 2.12 | 2.83 |
| Cd80 | Cd80 CD80 antigen | 0.22 | 0.23 | 0.66 | 0.40 | 1.06 | 2.83 |
| Ceacam2 | Ceacam2 carcinoembryonic antigen-related cell adhesion molecule 2 | 0.30 | 0.23 | 0.66 | 0.43 | 0.77 | 2.83 |
| Nox1 | Nox1 NADPH oxidase 1 | 0.08 | 0.23 | 0.66 | 0.55 | 2.82 | 2.83 |
| Tifa | Tifa TRAF-interacting protein with forkhead-associated domain | 5.74 | 6.94 | 19.60 | 11.65 | 1.21 | 2.82 |
| Sis | Sis sucrase isomaltase (alpha-glucosidase) | 0.11 | 0.21 | 0.59 | 0.89 | 1.88 | 2.82 |
| Cd44 | Cd44 CD44 antigen | 4.49 | 3.47 | 9.76 | 10.56 | 0.77 | 2.81 |
| Abcb1b | Abcb1b ATP-binding cassette, sub-family B (MDR/TAP), member 1B | 3.46 | 4.28 | 12.04 | 10.53 | 1.24 | 2.81 |
| Ttr | Ttr transthyretin | 2638.93 | 672.46 | 1886.37 | 1161.60 | 0.25 | 2.81 |
| Alx4 | Alx4 aristaless-like homeobox 4 | 1.91 | 0.57 | 1.61 | 2.04 | 0.30 | 2.80 |
| Edn1 | Edn1 endothelin 1 | 1.86 | 1.70 | 4.75 | 4.37 | 0.91 | 2.80 |
| Mir6904 | Mir6904 microRNA 6904 | 0.33 | 0.18 | 0.51 | 0.20 | 0.55 | 2.80 |
| Cers3 | Cers3 ceramide synthase 3 | 0.25 | 0.37 | 1.02 | 1.04 | 1.46 | 2.80 |
| Ankrd61 | Ankrd61 ankyrin repeat domain 61 | 0.55 | 0.89 | 2.48 | 1.61 | 1.60 | 2.79 |
| Txnip | Txnip thioredoxin interacting protein | 21.75 | 26.27 | 73.17 | 68.79 | 1.21 | 2.78 |
| Prdm1 | Prdm1 PR domain containing 1, with ZNF domain | 0.83 | 0.31 | 0.87 | 0.86 | 0.38 | 2.78 |
| Tbx18 | Tbx18 T-box18 | 1.75 | 1.10 | 3.04 | 2.07 | 0.63 | 2.78 |
| Cyp3a13 | Cyp3a13 cytochrome P450, family 3, subfamily a, polypeptide 13 | 0.42 | 0.73 | 2.02 | 1.24 | 1.76 | 2.76 |
| 1500015O10Rik | 1500015O10Rik RIKEN cDNA 1500015O10 gene | 31.51 | 7.85 | 21.67 | 11.51 | 0.25 | 2.76 |
| Entpd4 | Entpd4 ectonucleoside triphosphate diphosphohydrolase 4 | 7.15 | 2.14 | 5.90 | 2.22 | 0.30 | 2.76 |
| Cdh5 | Cdh5 cadherin 5 | 15.99 | 18.58 | 51.19 | 35.76 | 1.16 | 2.76 |
| AA467197 | AA467197 expressed sequence AA467197 | 0.14 | 0.55 | 1.51 | 1.81 | 3.95 | 2.75 |
| Ctxn3 | Ctxn3 cortexin 3 | 1.66 | 0.68 | 1.87 | 1.73 | 0.41 | 2.75 |
| Lrrc32 | Lrrc32 leucine rich repeat containing 32 | 3.88 | 9.65 | 26.50 | 24.02 | 2.49 | 2.75 |
| Rag1 | Rag1 recombination activating gene 1 | 0.22 | 0.13 | 0.36 | 0.23 | 0.59 | 2.74 |
| Hspb9 | Hspb9 heat shock protein, alpha-crystallin-related, B9 | 0.17 | 0.13 | 0.36 | 0.55 | 0.78 | 2.74 |
| Lrp2bp | Lrp2bp Lrp2 binding protein | 0.14 | 0.26 | 0.72 | 0.37 | 1.88 | 2.74 |
| Fam24a | Fam24a family with sequence similarity 24, member A | 0.06 | 0.26 | 0.72 | 0.60 | 4.71 | 2.74 |
| Spp1 | Spp1 secreted phosphoprotein 1 | 4.21 | 12.24 | 33.43 | 26.61 | 2.91 | 2.73 |
| Slc13a4 | Slc13a4 solute carrier family 13 (sodium/sulfate symporters), member 4 | 20.92 | 5.30 | 14.41 | 10.41 | 0.25 | 2.72 |
| F2rl3 | F2rl3 coagulation factor II (thrombin) receptor-like 3 | 0.58 | 0.23 | 0.64 | 0.23 | 0.40 | 2.72 |
| F5 | F5 coagulation factor V | 21.62 | 7.36 | 19.93 | 10.21 | 0.34 | 2.71 |
| Tgfbr3 | Tgfbr3 transforming growth factor, beta receptor III | 19.70 | 14.66 | 39.69 | 27.56 | 0.74 | 2.71 |
| Hs3st3b1 | Hs3st3b1 heparan sulfate (glucosamine) 3-O-sulfotransferase 3B1 | 0.72 | 0.55 | 1.48 | 1.41 | 0.76 | 2.71 |
| Aff1 | Aff1 AF4/FMR2 family, member 1 | 22.28 | 21.89 | 59.21 | 43.50 | 0.98 | 2.70 |
| 4930512B01Rik | 4930512B01Rik RIKEN cDNA 4930512B01 gene | 0.69 | 0.42 | 1.12 | 0.75 | 0.60 | 2.69 |
| Taf7l | Taf7l TATA-box binding protein associated factor 7 like | 0.22 | 0.42 | 1.12 | 0.95 | 1.88 | 2.69 |
| Btg3 | Btg3 B cell translocation gene 3 | 0.33 | 0.31 | 0.84 | 0.66 | 0.94 | 2.69 |
| Mir761 | Mir761 microRNA 761 | 0.17 | 0.10 | 0.28 | 0.12 | 0.63 | 2.69 |
| Pdzd9 | Pdzd9 PDZ domain containing 9 | 0.14 | 0.10 | 0.28 | 0.20 | 0.75 | 2.69 |
| Tnk1 | Tnk1 tyrosine kinase, non-receptor, 1 | 0.03 | 0.10 | 0.28 | 0.23 | 3.77 | 2.69 |
| Cnga3 | Cnga3 cyclic nucleotide gated channel alpha 3 | 0.11 | 0.10 | 0.28 | 0.23 | 0.94 | 2.69 |
| Pnpla1 | Pnpla1 patatin-like phospholipase domain containing 1 | 0.36 | 0.10 | 0.28 | 0.32 | 0.29 | 2.69 |
| Klf14 | Klf14 Kruppel-like factor 14 | 0.17 | 0.21 | 0.56 | 0.81 | 1.26 | 2.69 |
| Creb5 | Creb5 cAMP responsive element binding protein 5 | 2.02 | 1.15 | 3.09 | 2.07 | 0.57 | 2.69 |
| Ezr | Ezr ezrin | 65.46 | 74.86 | 201.18 | 151.64 | 1.14 | 2.69 |
| Sh3bgr | Sh3bgr SH3-binding domain glutamic acid-rich protein | 1.11 | 0.81 | 2.17 | 1.78 | 0.73 | 2.69 |
| Adamts9 | Adamts9 a disintegrin-like and metallopeptidase (reprolysin type) with thrombospondin type 1 motif, 9 | 8.90 | 12.05 | 32.33 | 22.93 | 1.36 | 2.68 |
| Mbip | Mbip MAP3K12 binding inhibitory protein 1 | 12.61 | 11.14 | 29.85 | 22.32 | 0.88 | 2.68 |
| Ifit3 | Ifit3 interferon-induced protein with tetratricopeptide repeats 3 | 3.82 | 9.63 | 25.76 | 21.89 | 2.52 | 2.68 |
| Birc3 | Birc3 baculoviral IAP repeat-containing 3 | 1.58 | 3.03 | 8.08 | 5.72 | 1.92 | 2.67 |
| Enpp2 | Enpp2 ectonucleotide pyrophosphatase/phosphodiesterase 2 | 624.39 | 198.32 | 528.89 | 265.65 | 0.32 | 2.67 |
| Cry1 | Cry1 cryptochrome 1 (photolyase-like) | 10.31 | 13.91 | 37.06 | 30.61 | 1.35 | 2.66 |
| Crispld2 | Crispld2 cysteine-rich secretory protein LCCL domain containing 2 | 4.49 | 6.16 | 16.41 | 13.72 | 1.37 | 2.66 |
| 4930528A17Rik | 4930528A17Rik RIKEN cDNA 4930528A17 gene | 0.30 | 0.52 | 1.38 | 1.21 | 1.71 | 2.64 |
| Gfpt2 | Gfpt2 glutamine fructose-6-phosphate transaminase 2 | 5.40 | 5.79 | 15.31 | 12.31 | 1.07 | 2.64 |
| Tnfrsf10b | Tnfrsf10b tumor necrosis factor receptor superfamily, member 10b | 0.94 | 1.38 | 3.65 | 2.07 | 1.47 | 2.64 |
| Fblim1 | Fblim1 filamin binding LIM protein 1 | 1.25 | 0.68 | 1.79 | 2.04 | 0.54 | 2.64 |
| Endou | Endou endonuclease, polyU-specific | 0.61 | 3.08 | 8.10 | 7.62 | 5.05 | 2.63 |
| Fbn1 | Fbn1 fibrillin 1 | 14.49 | 10.85 | 28.52 | 16.97 | 0.75 | 2.63 |
| Lrrc58 | Lrrc58 leucine rich repeat containing 58 | 73.99 | 88.89 | 233.30 | 159.78 | 1.20 | 2.62 |
| Snora81 | Snora81 small nucleolar RNA, H/ACA box 81 | 1.00 | 0.89 | 2.33 | 1.64 | 0.89 | 2.62 |
| Irak3 | Irak3 interleukin-1 receptor-associated kinase 3 | 1.08 | 1.59 | 4.17 | 5.55 | 1.47 | 2.62 |
| A930041C12Rik | A930041C12Rik RIKEN cDNA A930041C12 gene | 0.06 | 0.08 | 0.20 | 0.06 | 1.41 | 2.61 |
| Cep295nl | Cep295nl CEP295 N-terminal like | 0.14 | 0.08 | 0.20 | 0.09 | 0.56 | 2.61 |
| Ccdc169 | Ccdc169 coiled-coil domain containing 169 | 0.11 | 0.08 | 0.20 | 0.12 | 0.71 | 2.61 |
| A730090N16Rik | A730090N16Rik RIKEN cDNA A730090N16 gene | 0.11 | 0.08 | 0.20 | 0.12 | 0.71 | 2.61 |
| Mir7647 | Mir7647 microRNA 7647 | 0.03 | 0.08 | 0.20 | 0.14 | 2.82 | 2.61 |
| Mc1r | Mc1r melanocortin 1 receptor | 0.03 | 0.08 | 0.20 | 0.14 | 2.82 | 2.61 |
| Six1 | Six1 sine oculis-related homeobox 1 | 0.11 | 0.08 | 0.20 | 0.14 | 0.71 | 2.61 |
| Galr3 | Galr3 galanin receptor 3 | 0.11 | 0.08 | 0.20 | 0.17 | 0.71 | 2.61 |
| 4930482G09Rik | 4930482G09Rik RIKEN cDNA 4930482G09 gene | 0.14 | 0.08 | 0.20 | 0.20 | 0.56 | 2.61 |
| Spata46 | Spata46 spermatogenesis associated 46 | 0.19 | 0.08 | 0.20 | 0.20 | 0.40 | 2.61 |
| BB123696 | BB123696 expressed sequence BB123696 | 0.11 | 0.08 | 0.20 | 0.26 | 0.71 | 2.61 |
| Snord89 | Snord89 small nucleolar RNA, C/D box 89 | 0.19 | 0.08 | 0.20 | 0.26 | 0.40 | 2.61 |
| Ndc80 | Ndc80 NDC80 kinetochore complex component | 0.08 | 0.08 | 0.20 | 0.32 | 0.94 | 2.61 |
| F630028O10Rik | F630028O10Rik RIKEN cDNA F630028O10 gene | 0.03 | 0.08 | 0.20 | 0.35 | 2.82 | 2.61 |
| Cmah | Cmah cytidine monophospho-N-acetylneuraminic acid hydroxylase | 0.14 | 0.08 | 0.20 | 0.63 | 0.56 | 2.61 |
| Gm12185 | Gm12185 predicted gene 12185 | 0.03 | 0.16 | 0.41 | 0.32 | 5.65 | 2.61 |
| Tnnc2 | Tnnc2 troponin C2, fast | 0.03 | 0.16 | 0.41 | 0.35 | 5.65 | 2.61 |
| Acox2 | Acox2 acyl-Coenzyme A oxidase 2, branched chain | 0.44 | 0.23 | 0.61 | 0.23 | 0.53 | 2.61 |
| Moap1 | Moap1 modulator of apoptosis 1 | 0.39 | 0.23 | 0.61 | 0.26 | 0.61 | 2.61 |
| Dll4 | Dll4 delta like canonical Notch ligand 4 | 3.30 | 3.50 | 9.12 | 5.49 | 1.06 | 2.61 |
| Gtse1 | Gtse1 G two S phase expressed protein 1 | 0.64 | 0.68 | 1.76 | 1.93 | 1.06 | 2.60 |
| Nid1 | Nid1 nidogen 1 | 8.62 | 9.99 | 25.94 | 21.92 | 1.16 | 2.60 |
| Sgo2a | Sgo2a shugoshin 2A | 0.47 | 0.44 | 1.15 | 1.21 | 0.94 | 2.59 |
| Pram1 | Pram1 PML-RAR alpha-regulated adaptor molecule 1 | 1.72 | 1.44 | 3.71 | 3.16 | 0.84 | 2.58 |
| Gbp11 | Gbp11 guanylate binding protein 11 | 0.39 | 0.21 | 0.54 | 0.35 | 0.54 | 2.57 |
| Cwc22 | Cwc22 CWC22 spliceosome-associated protein | 12.19 | 10.83 | 27.75 | 18.50 | 0.89 | 2.56 |
| Adamts20 | Adamts20 a disintegrin-like and metallopeptidase (reprolysin type) with thrombospondin type 1 motif, 20 | 15.66 | 14.87 | 38.10 | 23.48 | 0.95 | 2.56 |
| Sox4 | Sox4 SRY (sex determining region Y)-box 4 | 11.75 | 11.14 | 28.52 | 23.13 | 0.95 | 2.56 |
| Dab2 | Dab2 disabled 2, mitogen-responsive phosphoprotein | 12.08 | 8.64 | 22.08 | 17.49 | 0.71 | 2.56 |
| Ctla2b | Ctla2b cytotoxic T lymphocyte-associated protein 2 beta | 0.58 | 2.40 | 6.13 | 7.85 | 4.12 | 2.56 |
| D330045A20Rik | D330045A20Rik RIKEN cDNA D330045A20 gene | 0.19 | 0.13 | 0.33 | 0.14 | 0.67 | 2.55 |
| Pcdha8 | Pcdha8 protocadherin alpha 8 | 0.39 | 0.13 | 0.33 | 0.14 | 0.34 | 2.55 |
| Muc15 | Muc15 mucin 15 | 0.03 | 0.13 | 0.33 | 0.17 | 4.71 | 2.55 |
| Sult6b2 | Sult6b2 sulfotransferase family 6B, member 2 | 0.19 | 0.13 | 0.33 | 0.17 | 0.67 | 2.55 |
| Gm12504 | Gm12504 predicted pseudogene 12504 | 0.14 | 0.13 | 0.33 | 0.20 | 0.94 | 2.55 |
| Olfr856-ps1 | Olfr856-ps1 olfactory receptor 856, pseudogene 1 | 0.22 | 0.13 | 0.33 | 0.35 | 0.59 | 2.55 |
| Rhoj | Rhoj ras homolog family member J | 3.13 | 6.44 | 16.41 | 15.68 | 2.06 | 2.55 |
| Kank1 | Kank1 KN motif and ankyrin repeat domains 1 | 18.84 | 18.34 | 46.69 | 30.93 | 0.97 | 2.55 |
| Armcx5 | Armcx5 armadillo repeat containing, X-linked 5 | 18.68 | 24.55 | 62.36 | 47.18 | 1.31 | 2.54 |
| Phf11d | Phf11d PHD finger protein 11D | 0.55 | 0.57 | 1.46 | 1.38 | 1.04 | 2.54 |
| Bmf | Bmf BCL2 modifying factor | 4.71 | 5.06 | 12.80 | 9.72 | 1.07 | 2.53 |
| Gimap6 | Gimap6 GTPase, IMAP family member 6 | 2.88 | 4.59 | 11.60 | 12.08 | 1.59 | 2.53 |
| Mvp | Mvp major vault protein | 9.70 | 19.75 | 49.89 | 43.44 | 2.04 | 2.53 |
| Csf3 | Csf3 colony stimulating factor 3 (granulocyte) | 0.08 | 7.18 | 18.12 | 27.79 | 86.31 | 2.53 |
| Pp2d1 | Pp2d1 protein phosphatase 2C-like domain containing 1 | 0.17 | 0.18 | 0.46 | 0.09 | 1.10 | 2.52 |
| Olfr543 | Olfr543 olfactory receptor 543 | 0.08 | 0.18 | 0.46 | 0.29 | 2.20 | 2.52 |
| Mybl2 | Mybl2 myeloblastosis oncogene-like 2 | 0.75 | 0.60 | 1.51 | 1.32 | 0.80 | 2.51 |
| Il13ra1 | Il13ra1 interleukin 13 receptor, alpha 1 | 5.21 | 5.22 | 13.08 | 8.66 | 1.00 | 2.51 |
| Cntd1 | Cntd1 cyclin N-terminal domain containing 1 | 0.72 | 0.70 | 1.76 | 1.35 | 0.98 | 2.50 |
| Tmprss11a | Tmprss11a transmembrane protease, serine 11a | 0.75 | 0.23 | 0.59 | 0.14 | 0.31 | 2.50 |
| Gm15694 | Gm15694 predicted gene 15694 | 0.17 | 0.29 | 0.72 | 0.37 | 1.73 | 2.49 |
| Gnl3 | Gnl3 guanine nucleotide binding protein-like 3 (nucleolar) | 19.81 | 19.20 | 47.84 | 33.43 | 0.97 | 2.49 |
| Fam76b | Fam76b family with sequence similarity 76, member B | 16.02 | 15.11 | 37.59 | 24.25 | 0.94 | 2.49 |
| Col8a1 | Col8a1 collagen, type VIII, alpha 1 | 8.37 | 2.61 | 6.49 | 2.45 | 0.31 | 2.49 |
| Rbm33 | Rbm33 RNA binding motif protein 33 | 73.13 | 59.49 | 147.36 | 118.53 | 0.81 | 2.48 |
| Tiparp | Tiparp TCDD-inducible poly(ADP-ribose) polymerase | 14.72 | 14.66 | 36.21 | 24.31 | 1.00 | 2.47 |
| Gbp5 | Gbp5 guanylate binding protein 5 | 3.02 | 7.15 | 17.61 | 26.73 | 2.37 | 2.46 |
| Adamts5 | Adamts5 a disintegrin-like and metallopeptidase (reprolysin type) with thrombospondin type 1 motif, 5 (aggrecanase-2) | 1.36 | 1.02 | 2.50 | 2.47 | 0.75 | 2.46 |
| Ubap1l | Ubap1l ubiquitin-associated protein 1-like | 1.30 | 1.44 | 3.53 | 2.65 | 1.10 | 2.46 |
| Ifi47 | Ifi47 interferon gamma inducible protein 47 | 1.08 | 2.58 | 6.34 | 8.83 | 2.39 | 2.45 |
| Rtl9 | Rtl9 retrotransposon Gag like 9 | 1.14 | 1.20 | 2.94 | 1.75 | 1.06 | 2.45 |
| Lrrc8e | Lrrc8e leucine rich repeat containing 8 family, member E | 0.14 | 0.10 | 0.26 | 0.12 | 0.75 | 2.45 |
| Pax8 | Pax8 paired box 8 | 0.03 | 0.10 | 0.26 | 0.32 | 3.77 | 2.45 |
| Baat | Baat bile acid-Coenzyme A: amino acid N-acyltransferase | 0.14 | 0.10 | 0.26 | 0.35 | 0.75 | 2.45 |
| Mir6236 | Mir6236 microRNA 6236 | 0.17 | 0.21 | 0.51 | 0.40 | 1.26 | 2.45 |
| Duox2 | Duox2 dual oxidase 2 | 0.14 | 0.10 | 0.26 | 0.46 | 0.75 | 2.45 |
| Trem1 | Trem1 triggering receptor expressed on myeloid cells 1 | 0.03 | 0.42 | 1.02 | 1.84 | 15.06 | 2.45 |
| Abi3bp | Abi3bp ABI gene family, member 3 (NESH) binding protein | 1.69 | 0.63 | 1.53 | 1.32 | 0.37 | 2.45 |
| Xlr3b | Xlr3b X-linked lymphocyte-regulated 3B | 2.00 | 0.37 | 0.89 | 0.81 | 0.18 | 2.45 |
| Serpind1 | Serpind1 serine (or cysteine) peptidase inhibitor, clade D, member 1 | 2.49 | 0.68 | 1.66 | 2.10 | 0.27 | 2.45 |
| Ly6g5b | Ly6g5b lymphocyte antigen 6 complex, locus G5B | 0.28 | 0.26 | 0.64 | 0.29 | 0.94 | 2.45 |
| Pkp3 | Pkp3 plakophilin 3 | 0.30 | 0.26 | 0.64 | 0.43 | 0.86 | 2.45 |
| 4833419F23Rik | 4833419F23Rik RIKEN cDNA 4833419F23 gene | 0.33 | 0.26 | 0.64 | 0.49 | 0.78 | 2.45 |
| E230013L22Rik | E230013L22Rik RIKEN cDNA E230013L22 gene | 0.75 | 0.26 | 0.64 | 0.63 | 0.35 | 2.45 |
| Cym | Cym chymosin | 0.03 | 0.16 | 0.38 | 0.60 | 5.65 | 2.45 |
| Gm1110 | Gm1110 predicted gene 1110 | 0.28 | 0.05 | 0.13 | 0.03 | 0.19 | 2.45 |
| F630111L10Rik | F630111L10Rik RIKEN cDNA F630111L10 gene | 0.03 | 0.05 | 0.13 | 0.06 | 1.88 | 2.45 |
| Muc4 | Muc4 mucin 4 | 0.06 | 0.05 | 0.13 | 0.09 | 0.94 | 2.45 |
| Slfn10-ps | Slfn10-ps schlafen 10, pseudogene | 0.08 | 0.05 | 0.13 | 0.09 | 0.63 | 2.45 |
| Adam3 | Adam3 a disintegrin and metallopeptidase domain 3 (cyritestin) | 0.36 | 0.05 | 0.13 | 0.09 | 0.14 | 2.45 |
| Zar1 | Zar1 zygote arrest 1 | 0.06 | 0.05 | 0.13 | 0.12 | 0.94 | 2.45 |
| Kcna10 | Kcna10 potassium voltage-gated channel, shaker-related subfamily, member 10 | 0.03 | 0.05 | 0.13 | 0.14 | 1.88 | 2.45 |
| Podnl1 | Podnl1 podocan-like 1 | 0.03 | 0.05 | 0.13 | 0.14 | 1.88 | 2.45 |
| 1700022I11Rik | 1700022I11Rik RIKEN cDNA 1700022I11 gene | 0.06 | 0.05 | 0.13 | 0.14 | 0.94 | 2.45 |
| Acoxl | Acoxl acyl-Coenzyme A oxidase-like | 0.11 | 0.05 | 0.13 | 0.14 | 0.47 | 2.45 |
| Hist2h2ab | Hist2h2ab histone cluster 2, H2ab | 0.06 | 0.05 | 0.13 | 0.17 | 0.94 | 2.45 |
| Trim10 | Trim10 tripartite motif-containing 10 | 0.06 | 0.05 | 0.13 | 0.17 | 0.94 | 2.45 |
| 4933413J09Rik | 4933413J09Rik RIKEN cDNA 4933413J09 gene | 0.11 | 0.05 | 0.13 | 0.17 | 0.47 | 2.45 |
| Lman1l | Lman1l lectin, mannose-binding 1 like | 0.22 | 0.05 | 0.13 | 0.20 | 0.24 | 2.45 |
| Folr1 | Folr1 folate receptor 1 (adult) | 39.05 | 8.90 | 21.75 | 17.78 | 0.23 | 2.44 |
| Clic6 | Clic6 chloride intracellular channel 6 | 27.55 | 10.12 | 24.74 | 11.13 | 0.37 | 2.44 |
| Dnah10 | Dnah10 dynein, axonemal, heavy chain 10 | 2.66 | 1.80 | 4.40 | 3.60 | 0.68 | 2.44 |
| Epc2 | Epc2 enhancer of polycomb homolog 2 | 34.67 | 32.95 | 80.43 | 56.21 | 0.95 | 2.44 |
| Pfkfb3 | Pfkfb3 6-phosphofructo-2-kinase/fructose-2,6-biphosphatase 3 | 37.05 | 34.26 | 83.52 | 77.27 | 0.92 | 2.44 |
| Palb2 | Palb2 partner and localizer of BRCA2 | 1.36 | 0.91 | 2.22 | 1.96 | 0.67 | 2.43 |
| Perp | Perp PERP, TP53 apoptosis effector | 4.05 | 2.48 | 6.03 | 4.32 | 0.61 | 2.43 |
| D830031N03Rik | D830031N03Rik RIKEN cDNA D830031N03 gene | 10.92 | 9.50 | 23.10 | 19.07 | 0.87 | 2.43 |
| Abca4 | Abca4 ATP-binding cassette, sub-family A (ABC1), member 4 | 12.17 | 5.24 | 12.75 | 8.26 | 0.43 | 2.43 |
| Rlf | Rlf rearranged L-myc fusion sequence | 31.09 | 28.57 | 69.44 | 41.43 | 0.92 | 2.43 |
| Hhex | Hhex hematopoietically expressed homeobox | 1.91 | 1.15 | 2.79 | 1.44 | 0.60 | 2.43 |
| **Cnn1** | **Cnn1 calponin 1** | **1.30** | **0.55** | **1.33** | **0.95** | **0.42** | **2.43** |
| Sdcbp2 | Sdcbp2 syndecan binding protein (syntenin) 2 | 2.33 | 3.42 | 8.28 | 8.03 | 1.47 | 2.42 |
| Nfkbiz | Nfkbiz nuclear factor of kappa light polypeptide gene enhancer in B cells inhibitor, zeta | 6.40 | 8.95 | 21.62 | 14.18 | 1.40 | 2.42 |
| Rbmx2 | Rbmx2 RNA binding motif protein, X-linked 2 | 4.46 | 3.60 | 8.69 | 6.82 | 0.81 | 2.41 |
| Rel | Rel reticuloendotheliosis oncogene | 1.16 | 1.07 | 2.58 | 1.32 | 0.92 | 2.41 |
| Mx2 | Mx2 MX dynamin-like GTPase 2 | 0.44 | 1.77 | 4.27 | 2.91 | 4.00 | 2.41 |
| Wdr86 | Wdr86 WD repeat domain 86 | 7.57 | 3.34 | 8.02 | 7.05 | 0.44 | 2.40 |
| Stat3 | Stat3 signal transducer and activator of transcription 3 | 48.30 | 67.03 | 161.01 | 133.31 | 1.39 | 2.40 |
| Wdhd1 | Wdhd1 WD repeat and HMG-box DNA binding protein 1 | 5.74 | 4.77 | 11.45 | 8.46 | 0.83 | 2.40 |
| Anxa1 | Anxa1 annexin A1 | 0.97 | 0.70 | 1.69 | 1.78 | 0.73 | 2.39 |
| Mctp2 | Mctp2 multiple C2 domains, transmembrane 2 | 1.05 | 1.17 | 2.81 | 2.39 | 1.11 | 2.39 |
| Apoh | Apoh apolipoprotein H | 0.22 | 0.23 | 0.56 | 0.29 | 1.06 | 2.39 |
| Acsm3 | Acsm3 acyl-CoA synthetase medium-chain family member 3 | 0.22 | 0.23 | 0.56 | 0.60 | 1.06 | 2.39 |
| Loxhd1 | Loxhd1 lipoxygenase homology domains 1 | 0.17 | 0.23 | 0.56 | 0.98 | 1.41 | 2.39 |
| Maml2 | Maml2 mastermind like transcriptional coactivator 2 | 19.70 | 16.31 | 38.95 | 25.81 | 0.83 | 2.39 |
| 4930419G24Rik | 4930419G24Rik RIKEN cDNA 4930419G24 gene | 0.47 | 0.42 | 1.00 | 0.69 | 0.89 | 2.39 |
| Mob1b | Mob1b MOB kinase activator 1B | 12.64 | 11.04 | 26.32 | 15.28 | 0.87 | 2.39 |
| Tada2b | Tada2b transcriptional adaptor 2B | 35.06 | 33.40 | 79.45 | 52.85 | 0.95 | 2.38 |
| Gprc5a | Gprc5a G protein-coupled receptor, family C, group 5, member A | 0.17 | 0.55 | 1.30 | 0.95 | 3.30 | 2.38 |
| Dyrk1a | Dyrk1a dual-specificity tyrosine-(Y)-phosphorylation regulated kinase 1a | 67.29 | 58.78 | 139.72 | 99.48 | 0.87 | 2.38 |
| Fzd5 | Fzd5 frizzled class receptor 5 | 1.77 | 1.41 | 3.35 | 2.04 | 0.79 | 2.38 |
| Rin2 | Rin2 Ras and Rab interactor 2 | 25.02 | 23.46 | 55.71 | 37.14 | 0.94 | 2.38 |
| Ssc5d | Ssc5d scavenger receptor cysteine rich family, 5 domains | 3.66 | 2.84 | 6.75 | 5.61 | 0.78 | 2.37 |
| Rora | Rora RAR-related orphan receptor alpha | 31.29 | 25.54 | 60.59 | 38.09 | 0.82 | 2.37 |
| Vwce | Vwce von Willebrand factor C and EGF domains | 1.05 | 0.50 | 1.18 | 0.92 | 0.47 | 2.37 |
| Tram2 | Tram2 translocating chain-associating membrane protein 2 | 0.53 | 0.50 | 1.18 | 0.95 | 0.94 | 2.37 |
| Gpr65 | Gpr65 G-protein coupled receptor 65 | 0.19 | 0.81 | 1.92 | 1.67 | 4.17 | 2.37 |
| Lta | Lta lymphotoxin A | 0.19 | 0.31 | 0.74 | 0.95 | 1.61 | 2.37 |
| Gm9079 | Gm9079 predicted gene 9079 | 1.50 | 0.76 | 1.79 | 1.44 | 0.51 | 2.36 |
| Hp | Hp haptoglobin | 0.33 | 0.76 | 1.79 | 2.04 | 2.28 | 2.36 |
| Ccl3 | Ccl3 chemokine (C-C motif) ligand 3 | 0.33 | 1.20 | 2.84 | 3.14 | 3.61 | 2.36 |
| Dnajb13 | Dnajb13 DnaJ heat shock protein family (Hsp40) member B13 | 1.33 | 0.57 | 1.35 | 0.83 | 0.43 | 2.36 |
| Sfn | Sfn stratifin | 1.80 | 1.49 | 3.50 | 2.30 | 0.83 | 2.35 |
| Elf3 | Elf3 E74-like factor 3 | 0.08 | 0.13 | 0.31 | 0.17 | 1.57 | 2.35 |
| Scn4a | Scn4a sodium channel, voltage-gated, type IV, alpha | 0.08 | 0.13 | 0.31 | 0.23 | 1.57 | 2.35 |
| Gm4922 | Gm4922 predicted gene 4922 | 0.19 | 0.13 | 0.31 | 0.29 | 0.67 | 2.35 |
| Pif1 | Pif1 PIF1 5'-to-3' DNA helicase | 0.25 | 0.13 | 0.31 | 0.37 | 0.52 | 2.35 |
| Top2a | Top2a topoisomerase (DNA) II alpha | 0.80 | 0.26 | 0.61 | 0.63 | 0.32 | 2.35 |
| Gab2 | Gab2 growth factor receptor bound protein 2-associated protein 2 | 23.00 | 21.37 | 50.07 | 37.37 | 0.93 | 2.34 |
| Plp1 | Plp1 proteolipid protein (myelin) 1 | 842.02 | 340.21 | 796.93 | 561.16 | 0.40 | 2.34 |
| Tex30 | Tex30 testis expressed 30 | 2.19 | 2.61 | 6.11 | 4.80 | 1.19 | 2.34 |
| Onecut2 | Onecut2 one cut domain, family member 2 | 8.73 | 6.37 | 14.85 | 8.00 | 0.73 | 2.33 |
| Tcim | Tcim transcriptional and immune response regulator | 0.97 | 0.55 | 1.28 | 1.04 | 0.56 | 2.33 |
| Pde4b | Pde4b phosphodiesterase 4B, cAMP specific | 59.69 | 55.21 | 128.68 | 85.64 | 0.92 | 2.33 |
| Atp10d | Atp10d ATPase, class V, type 10D | 3.80 | 3.39 | 7.87 | 3.94 | 0.89 | 2.32 |
| Cdk3-ps | Cdk3-ps cyclin-dependent kinase 3, pseudogene | 1.86 | 1.23 | 2.84 | 2.91 | 0.66 | 2.31 |
| Nkx3-1 | Nkx3-1 NK3 homeobox 1 | 0.89 | 1.10 | 2.53 | 2.07 | 1.24 | 2.31 |
| Hacd4 | Hacd4 3-hydroxyacyl-CoA dehydratase 4 | 1.80 | 1.17 | 2.71 | 1.70 | 0.65 | 2.31 |
| Snord22 | Snord22 small nucleolar RNA, C/D box 22 | 0.50 | 0.44 | 1.02 | 0.78 | 0.89 | 2.30 |
| Cd53 | Cd53 CD53 antigen | 4.91 | 5.27 | 12.11 | 9.18 | 1.07 | 2.30 |
| Atxn1l | Atxn1l ataxin 1-like | 40.18 | 35.09 | 80.37 | 54.55 | 0.87 | 2.29 |
| Setdb2 | Setdb2 SET domain, bifurcated 2 | 4.30 | 4.04 | 9.25 | 9.84 | 0.94 | 2.29 |
| Mlf1 | Mlf1 myeloid leukemia factor 1 | 1.30 | 0.39 | 0.89 | 0.63 | 0.30 | 2.29 |
| Comp | Comp cartilage oligomeric matrix protein | 0.72 | 0.39 | 0.89 | 0.86 | 0.54 | 2.29 |
| Abcc12 | Abcc12 ATP-binding cassette, sub-family C (CFTR/MRP), member 12 | 0.36 | 0.31 | 0.72 | 0.58 | 0.87 | 2.29 |
| 4921536K21Rik | 4921536K21Rik RIKEN cDNA 4921536K21 gene | 0.72 | 0.31 | 0.72 | 0.69 | 0.43 | 2.29 |
| Ptafr | Ptafr platelet-activating factor receptor | 0.58 | 0.31 | 0.72 | 0.83 | 0.54 | 2.29 |
| Lrrc26 | Lrrc26 leucine rich repeat containing 26 | 1.52 | 1.57 | 3.58 | 3.11 | 1.03 | 2.29 |
| Gm10416 | Gm10416 predicted pseudogene 10416 | 0.11 | 0.08 | 0.18 | 0.09 | 0.71 | 2.29 |
| Mir7037 | Mir7037 microRNA 7037 | 0.14 | 0.08 | 0.18 | 0.09 | 0.56 | 2.29 |
| Zp3r | Zp3r zona pellucida 3 receptor | 0.03 | 0.08 | 0.18 | 0.12 | 2.82 | 2.29 |
| D6Ertd474e | D6Ertd474e DNA segment, Chr 6, ERATO Doi 474, expressed | 0.08 | 0.08 | 0.18 | 0.12 | 0.94 | 2.29 |
| Cxcr6 | Cxcr6 chemokine (C-X-C motif) receptor 6 | 0.19 | 0.08 | 0.18 | 0.14 | 0.40 | 2.29 |
| Edar | Edar ectodysplasin-A receptor | 0.28 | 0.08 | 0.18 | 0.14 | 0.28 | 2.29 |
| Slamf7 | Slamf7 SLAM family member 7 | 0.03 | 0.08 | 0.18 | 0.17 | 2.82 | 2.29 |
| Snord23 | Snord23 small nucleolar RNA, C/D box 23 | 0.03 | 0.08 | 0.18 | 0.23 | 2.82 | 2.29 |
| Gm4952 | Gm4952 predicted gene 4952 | 0.17 | 0.08 | 0.18 | 0.23 | 0.47 | 2.29 |
| Cacng6 | Cacng6 calcium channel, voltage-dependent, gamma subunit 6 | 0.89 | 0.70 | 1.61 | 1.24 | 0.79 | 2.29 |
| Gm25500 | Gm25500 predicted gene, 25500 | 0.44 | 0.23 | 0.54 | 0.14 | 0.53 | 2.29 |
| Irf4 | Irf4 interferon regulatory factor 4 | 0.44 | 0.23 | 0.54 | 0.49 | 0.53 | 2.29 |
| Tcam1 | Tcam1 testicular cell adhesion molecule 1 | 0.36 | 0.23 | 0.54 | 0.58 | 0.65 | 2.29 |
| Cd86 | Cd86 CD86 antigen | 1.50 | 2.06 | 4.70 | 2.68 | 1.38 | 2.28 |
| Dsp | Dsp desmoplakin | 49.83 | 36.01 | 81.91 | 49.51 | 0.72 | 2.27 |
| Klf9 | Klf9 Kruppel-like factor 9 | 108.08 | 106.14 | 241.18 | 184.55 | 0.98 | 2.27 |
| Dcaf10 | Dcaf10 DDB1 and CUL4 associated factor 10 | 18.18 | 17.30 | 39.31 | 28.48 | 0.95 | 2.27 |
| Dram1 | Dram1 DNA-damage regulated autophagy modulator 1 | 1.27 | 1.07 | 2.43 | 2.10 | 0.84 | 2.27 |
| Dis3 | Dis3 DIS3 homolog, exosome endoribonuclease and 3'-5' exoribonuclease | 11.89 | 10.49 | 23.77 | 19.48 | 0.88 | 2.27 |
| Smad1 | Smad1 SMAD family member 1 | 15.63 | 16.78 | 38.00 | 27.56 | 1.07 | 2.27 |
| Tent5b | Tent5b terminal nucleotidyltransferase 5B | 0.30 | 0.83 | 1.89 | 2.27 | 2.74 | 2.27 |
| Mcc | Mcc mutated in colorectal cancers | 14.05 | 13.78 | 31.18 | 21.29 | 0.98 | 2.26 |
| Elf4 | Elf4 E74-like factor 4 (ets domain transcription factor) | 1.91 | 2.64 | 5.95 | 4.14 | 1.38 | 2.26 |
| Myh11 | Myh11 myosin, heavy polypeptide 11, smooth muscle | 13.86 | 5.27 | 11.91 | 8.31 | 0.38 | 2.26 |
| Filip1l | Filip1l filamin A interacting protein 1-like | 3.02 | 2.95 | 6.64 | 4.63 | 0.98 | 2.25 |
| Synpo2l | Synpo2l synaptopodin 2-like | 0.25 | 0.52 | 1.18 | 1.55 | 2.09 | 2.25 |
| Insig1 | Insig1 insulin induced gene 1 | 47.31 | 26.90 | 60.57 | 40.05 | 0.57 | 2.25 |
| Col8a2 | Col8a2 collagen, type VIII, alpha 2 | 13.94 | 4.83 | 10.86 | 5.09 | 0.35 | 2.25 |
| Fut7 | Fut7 fucosyltransferase 7 | 0.36 | 0.70 | 1.58 | 2.53 | 1.96 | 2.25 |
| Tma16 | Tma16 translation machinery associated 16 | 2.94 | 4.41 | 9.92 | 7.83 | 1.50 | 2.25 |
| Chek1 | Chek1 checkpoint kinase 1 | 0.67 | 0.63 | 1.41 | 0.89 | 0.94 | 2.24 |
| Bnipl | Bnipl BCL2/adenovirus E1B 19kD interacting protein like | 0.11 | 0.18 | 0.41 | 0.37 | 1.65 | 2.24 |
| B230208H11Rik | B230208H11Rik RIKEN cDNA B230208H11 gene | 0.14 | 0.18 | 0.41 | 0.37 | 1.32 | 2.24 |
| Ankle1 | Ankle1 ankyrin repeat and LEM domain containing 1 | 0.28 | 0.18 | 0.41 | 0.37 | 0.66 | 2.24 |
| Cip2a | Cip2a cell proliferation regulating inhibitor of protein phosphatase 2A | 2.19 | 1.57 | 3.50 | 2.82 | 0.72 | 2.24 |
| Pkd1l3 | Pkd1l3 polycystic kidney disease 1 like 3 | 4.24 | 3.81 | 8.51 | 5.90 | 0.90 | 2.23 |
| Nid2 | Nid2 nidogen 2 | 6.79 | 3.63 | 8.10 | 4.69 | 0.53 | 2.23 |
| Rdh9 | Rdh9 retinol dehydrogenase 9 | 0.42 | 0.65 | 1.46 | 2.13 | 1.57 | 2.23 |
| Zfp143 | Zfp143 zinc finger protein 143 | 8.06 | 5.35 | 11.93 | 9.18 | 0.66 | 2.23 |
| Dhx33 | Dhx33 DEAH (Asp-Glu-Ala-His) box polypeptide 33 | 74.85 | 72.95 | 162.46 | 139.64 | 0.97 | 2.23 |
| Cdc42ep3 | Cdc42ep3 CDC42 effector protein (Rho GTPase binding) 3 | 4.49 | 5.06 | 11.27 | 8.52 | 1.13 | 2.23 |
| 1700028K03Rik | 1700028K03Rik RIKEN cDNA 1700028K03 gene | 1.08 | 1.15 | 2.56 | 1.47 | 1.06 | 2.23 |
| Tagln | Tagln transgelin | 17.63 | 8.90 | 19.81 | 19.42 | 0.50 | 2.23 |
| Dopey1 | Dopey1 dopey family member 1 | 43.29 | 41.20 | 91.70 | 69.68 | 0.95 | 2.23 |
| Alg10b | Alg10b asparagine-linked glycosylation 10B (alpha-1,2-glucosyltransferase) | 25.88 | 21.60 | 48.07 | 35.53 | 0.83 | 2.23 |
| Acr | Acr acrosin prepropeptide | 1.19 | 1.64 | 3.65 | 3.02 | 1.38 | 2.22 |
| Fkbp5 | Fkbp5 FK506 binding protein 5 | 53.96 | 75.51 | 167.83 | 166.37 | 1.40 | 2.22 |
| Errfi1 | Errfi1 ERBB receptor feedback inhibitor 1 | 34.20 | 41.07 | 91.26 | 81.82 | 1.20 | 2.22 |
| Fgfbp1 | Fgfbp1 fibroblast growth factor binding protein 1 | 0.80 | 0.78 | 1.74 | 1.87 | 0.97 | 2.22 |
| Crabp2 | Crabp2 cellular retinoic acid binding protein II | 2.33 | 0.99 | 2.20 | 2.01 | 0.43 | 2.22 |
| Abhd18 | Abhd18 abhydrolase domain containing 18 | 8.01 | 5.98 | 13.24 | 10.44 | 0.75 | 2.22 |
| Heca | Heca hdc homolog, cell cycle regulator | 19.34 | 19.28 | 42.65 | 33.20 | 1.00 | 2.21 |
| Usp53 | Usp53 ubiquitin specific peptidase 53 | 13.25 | 13.54 | 29.90 | 24.05 | 1.02 | 2.21 |
| Ano7 | Ano7 anoctamin 7 | 0.25 | 0.21 | 0.46 | 0.49 | 0.84 | 2.20 |
| Lncpint | Lncpint long non-protein coding RNA, Trp53 induced transcript | 0.14 | 0.31 | 0.69 | 0.37 | 2.26 | 2.20 |
| Kdelr3 | Kdelr3 KDEL (Lys-Asp-Glu-Leu) endoplasmic reticulum protein retention receptor 3 | 0.97 | 0.42 | 0.92 | 0.78 | 0.43 | 2.20 |
| A330015K06Rik | A330015K06Rik RIKEN cDNA A330015K06 gene | 0.75 | 0.42 | 0.92 | 0.83 | 0.56 | 2.20 |
| Ckap2 | Ckap2 cytoskeleton associated protein 2 | 0.50 | 0.42 | 0.92 | 1.01 | 0.84 | 2.20 |
| 4930430J02Rik | 4930430J02Rik RIKEN cDNA 4930430J02 gene | 0.11 | 0.10 | 0.23 | 0.03 | 0.94 | 2.20 |
| Gm3264 | Gm3264 predicted gene 3264 | 0.14 | 0.10 | 0.23 | 0.09 | 0.75 | 2.20 |
| Slc35g3 | Slc35g3 solute carrier family 35, member G3 | 0.42 | 0.10 | 0.23 | 0.09 | 0.25 | 2.20 |
| 1700025N23Rik | 1700025N23Rik RIKEN cDNA 1700025N23 gene | 0.03 | 0.10 | 0.23 | 0.12 | 3.77 | 2.20 |
| Foxd2os | Foxd2os forkhead box D2, opposite strand | 0.06 | 0.10 | 0.23 | 0.14 | 1.88 | 2.20 |
| 1500015L24Rik | 1500015L24Rik RIKEN cDNA 1500015L24 gene | 0.44 | 0.10 | 0.23 | 0.14 | 0.24 | 2.20 |
| Mir7682 | Mir7682 microRNA 7682 | 0.08 | 0.10 | 0.23 | 0.20 | 1.26 | 2.20 |
| Krt26 | Krt26 keratin 26 | 0.17 | 0.10 | 0.23 | 0.26 | 0.63 | 2.20 |
| Foxd2 | Foxd2 forkhead box D2 | 0.33 | 0.10 | 0.23 | 0.29 | 0.31 | 2.20 |
| C130080G10Rik | C130080G10Rik RIKEN cDNA C130080G10 gene | 0.03 | 0.10 | 0.23 | 0.46 | 3.77 | 2.20 |
| Rdh18-ps | Rdh18-ps retinol dehydrogenase 18, pseudogene | 0.06 | 0.10 | 0.23 | 0.55 | 1.88 | 2.20 |
| Kif20a | Kif20a kinesin family member 20A | 2.99 | 2.71 | 5.98 | 4.69 | 0.91 | 2.20 |
| Kif24 | Kif24 kinesin family member 24 | 0.89 | 0.63 | 1.38 | 0.86 | 0.71 | 2.20 |
| Wnt6 | Wnt6 wingless-type MMTV integration site family, member 6 | 1.58 | 0.52 | 1.15 | 1.29 | 0.33 | 2.20 |
| Rbm7 | Rbm7 RNA binding motif protein 7 | 20.09 | 17.56 | 38.69 | 29.37 | 0.87 | 2.20 |
| Tead1 | Tead1 TEA domain family member 1 | 34.59 | 28.57 | 62.89 | 49.05 | 0.83 | 2.20 |
| Tmcc3 | Tmcc3 transmembrane and coiled coil domains 3 | 48.50 | 34.91 | 76.72 | 52.53 | 0.72 | 2.20 |
| D7Ertd443e | D7Ertd443e DNA segment, Chr 7, ERATO Doi 443, expressed | 2.74 | 2.04 | 4.47 | 4.66 | 0.74 | 2.20 |
| Lats1 | Lats1 large tumor suppressor | 36.80 | 33.35 | 73.19 | 51.93 | 0.91 | 2.20 |
| Slc43a1 | Slc43a1 solute carrier family 43, member 1 | 0.36 | 0.65 | 1.43 | 2.59 | 1.81 | 2.19 |
| Lrrn4cl | Lrrn4cl LRRN4 C-terminal like | 0.89 | 0.55 | 1.20 | 0.98 | 0.62 | 2.19 |
| Tfdp2 | Tfdp2 transcription factor Dp 2 | 31.09 | 28.70 | 62.87 | 44.97 | 0.92 | 2.19 |
| Tmem154 | Tmem154 transmembrane protein 154 | 0.22 | 0.89 | 1.94 | 3.62 | 4.00 | 2.19 |
| Cyp1b1 | Cyp1b1 cytochrome P450 family 1 subfamily b polypeptide 1 | 4.27 | 4.49 | 9.81 | 14.15 | 1.05 | 2.19 |
| Arhgap8 | Arhgap8 Rho GTPase activating protein 8 | 0.25 | 0.34 | 0.74 | 0.63 | 1.36 | 2.19 |
| Topbp1 | Topbp1 topoisomerase (DNA) II binding protein 1 | 17.65 | 12.71 | 27.73 | 22.70 | 0.72 | 2.18 |
| Krt18 | Krt18 keratin 18 | 4.71 | 2.06 | 4.50 | 3.34 | 0.44 | 2.18 |
| Rpph1 | Rpph1 ribonuclease P RNA component H1 | 0.28 | 0.47 | 1.02 | 0.29 | 1.69 | 2.18 |
| Ptger2 | Ptger2 prostaglandin E receptor 2 (subtype EP2) | 0.30 | 0.23 | 0.51 | 0.55 | 0.77 | 2.18 |
| Aldh1a2 | Aldh1a2 aldehyde dehydrogenase family 1, subfamily A2 | 7.32 | 4.83 | 10.50 | 8.00 | 0.66 | 2.18 |
| Wfikkn1 | Wfikkn1 WAP, FS, Ig, KU, and NTR-containing protein 1 | 0.50 | 7.31 | 15.90 | 4.55 | 14.65 | 2.18 |
| Lonrf3 | Lonrf3 LON peptidase N-terminal domain and ring finger 3 | 23.31 | 23.22 | 50.40 | 44.85 | 1.00 | 2.17 |
| Avil | Avil advillin | 0.64 | 0.73 | 1.58 | 1.38 | 1.15 | 2.17 |
| Hmgb3 | Hmgb3 high mobility group box 3 | 6.65 | 8.56 | 18.55 | 16.20 | 1.29 | 2.17 |
| Hnrnpdl | Hnrnpdl heterogeneous nuclear ribonucleoprotein D-like | 131.19 | 107.11 | 232.18 | 179.95 | 0.82 | 2.17 |
| Gabpb1 | Gabpb1 GA repeat binding protein, beta 1 | 12.72 | 10.98 | 23.74 | 18.44 | 0.86 | 2.16 |
| Nhlrc3 | Nhlrc3 NHL repeat containing 3 | 3.57 | 3.47 | 7.49 | 6.10 | 0.97 | 2.16 |
| Dhx15 | Dhx15 DEAH (Asp-Glu-Ala-His) box polypeptide 15 | 63.52 | 59.75 | 128.88 | 95.25 | 0.94 | 2.16 |
| 4933408B17Rik | 4933408B17Rik RIKEN cDNA 4933408B17 gene | 0.55 | 0.65 | 1.41 | 1.21 | 1.18 | 2.15 |
| Ihh | Ihh Indian hedgehog | 0.22 | 0.13 | 0.28 | 0.12 | 0.59 | 2.15 |
| 4930550C14Rik | 4930550C14Rik RIKEN cDNA 4930550C14 gene | 0.25 | 0.13 | 0.28 | 0.14 | 0.52 | 2.15 |
| Rdh1 | Rdh1 retinol dehydrogenase 1 (all trans) | 0.08 | 0.13 | 0.28 | 0.37 | 1.57 | 2.15 |
| Loxl4 | Loxl4 lysyl oxidase-like 4 | 0.53 | 0.39 | 0.84 | 0.35 | 0.74 | 2.15 |
| Slc22a2 | Slc22a2 solute carrier family 22 (organic cation transporter), member 2 | 0.67 | 0.26 | 0.56 | 0.40 | 0.39 | 2.15 |
| Mss51 | Mss51 MSS51 mitochondrial translational activator | 0.36 | 0.26 | 0.56 | 0.66 | 0.72 | 2.15 |
| Arl4a | Arl4a ADP-ribosylation factor-like 4A | 23.17 | 22.44 | 48.33 | 35.04 | 0.97 | 2.15 |
| Hells | Hells helicase, lymphoid specific | 1.58 | 0.94 | 2.02 | 1.32 | 0.59 | 2.15 |
| Zranb1 | Zranb1 zinc finger, RAN-binding domain containing 1 | 28.90 | 27.66 | 59.27 | 41.20 | 0.96 | 2.14 |
| Fut4 | Fut4 fucosyltransferase 4 | 0.25 | 0.29 | 0.61 | 0.52 | 1.15 | 2.14 |
| Snora16a | Snora16a small nucleolar RNA, H/ACA box 16A | 0.67 | 0.29 | 0.61 | 0.60 | 0.43 | 2.14 |
| Ska3 | Ska3 spindle and kinetochore associated complex subunit 3 | 1.14 | 0.86 | 1.84 | 1.87 | 0.76 | 2.14 |
| Wwtr1 | Wwtr1 WW domain containing transcription regulator 1 | 16.13 | 13.93 | 29.77 | 19.62 | 0.86 | 2.14 |
| Marveld2 | Marveld2 MARVEL (membrane-associating) domain containing 2 | 0.97 | 1.02 | 2.17 | 1.44 | 1.05 | 2.13 |
| Il6ra | Il6ra interleukin 6 receptor, alpha | 5.40 | 6.91 | 14.75 | 13.78 | 1.28 | 2.13 |
| Adap2 | Adap2 ArfGAP with dual PH domains 2 | 4.05 | 3.68 | 7.85 | 5.81 | 0.91 | 2.13 |
| Mast4 | Mast4 microtubule associated serine/threonine kinase family member 4 | 66.34 | 58.11 | 123.90 | 94.48 | 0.88 | 2.13 |
| Nek5 | Nek5 NIMA (never in mitosis gene a)-related expressed kinase 5 | 1.03 | 0.44 | 0.95 | 0.52 | 0.43 | 2.13 |
| Gm11517 | Gm11517 predicted gene 11517 | 0.64 | 0.89 | 1.89 | 1.50 | 1.39 | 2.13 |
| Timp1 | Timp1 tissue inhibitor of metalloproteinase 1 | 0.47 | 3.94 | 8.38 | 10.56 | 8.36 | 2.13 |
| Fam89a | Fam89a family with sequence similarity 89, member A | 1.47 | 1.07 | 2.27 | 1.99 | 0.73 | 2.13 |
| Lpxn | Lpxn leupaxin | 1.91 | 1.38 | 2.94 | 3.31 | 0.72 | 2.13 |
| Zfp36 | Zfp36 zinc finger protein 36 | 5.76 | 14.69 | 31.20 | 28.71 | 2.55 | 2.12 |
| Prr11 | Prr11 proline rich 11 | 0.53 | 0.31 | 0.66 | 0.92 | 0.59 | 2.12 |
| Gpr152 | Gpr152 G protein-coupled receptor 152 | 0.11 | 0.16 | 0.33 | 0.12 | 1.41 | 2.12 |
| Adh1 | Adh1 alcohol dehydrogenase 1 (class I) | 0.64 | 0.16 | 0.33 | 0.20 | 0.25 | 2.12 |
| Camp | Camp cathelicidin antimicrobial peptide | 0.03 | 0.16 | 0.33 | 0.23 | 5.65 | 2.12 |
| Qrfp | Qrfp pyroglutamylated RFamide peptide | 0.14 | 0.16 | 0.33 | 0.23 | 1.13 | 2.12 |
| Odf3 | Odf3 outer dense fiber of sperm tails 3 | 0.28 | 0.16 | 0.33 | 0.26 | 0.56 | 2.12 |
| 4931423N10Rik | 4931423N10Rik RIKEN cDNA 4931423N10 gene | 0.17 | 0.16 | 0.33 | 0.35 | 0.94 | 2.12 |
| Has2 | Has2 hyaluronan synthase 2 | 0.22 | 0.16 | 0.33 | 0.37 | 0.71 | 2.12 |
| Mylk4 | Mylk4 myosin light chain kinase family, member 4 | 0.17 | 0.16 | 0.33 | 0.72 | 0.94 | 2.12 |
| Kdm5a | Kdm5a lysine (K)-specific demethylase 5A | 42.48 | 37.83 | 80.07 | 54.29 | 0.89 | 2.12 |
| Siah1a | Siah1a siah E3 ubiquitin protein ligase 1A | 18.26 | 15.76 | 33.35 | 23.45 | 0.86 | 2.12 |
| Flrt3 | Flrt3 fibronectin leucine rich transmembrane protein 3 | 28.65 | 23.90 | 50.47 | 43.21 | 0.83 | 2.11 |
| E2f7 | E2f7 E2F transcription factor 7 | 0.36 | 0.34 | 0.72 | 0.29 | 0.94 | 2.11 |
| Serpina3i | Serpina3i serine (or cysteine) peptidase inhibitor, clade A, member 3I | 0.08 | 0.34 | 0.72 | 0.98 | 4.08 | 2.11 |
| Tbc1d15 | Tbc1d15 TBC1 domain family, member 15 | 26.27 | 23.74 | 49.99 | 33.54 | 0.90 | 2.11 |
| Samd8 | Samd8 sterile alpha motif domain containing 8 | 72.66 | 60.77 | 127.94 | 95.34 | 0.84 | 2.11 |
| Slc4a5 | Slc4a5 solute carrier family 4, sodium bicarbonate cotransporter, member 5 | 12.78 | 4.20 | 8.84 | 4.06 | 0.33 | 2.10 |
| Mms22l | Mms22l MMS22-like, DNA repair protein | 0.67 | 1.07 | 2.25 | 1.67 | 1.61 | 2.10 |
| Adgrg3 | Adgrg3 adhesion G protein-coupled receptor G3 | 0.19 | 1.25 | 2.63 | 3.19 | 6.46 | 2.10 |
| Gldn | Gldn gliomedin | 1.30 | 2.35 | 4.93 | 5.84 | 1.80 | 2.10 |
| Ifi208 | Ifi208 interferon activated gene 208 | 0.03 | 0.18 | 0.38 | 0.14 | 6.59 | 2.10 |
| Mip | Mip major intrinsic protein of lens fiber | 0.22 | 0.18 | 0.38 | 0.14 | 0.82 | 2.10 |
| Gm8234 | Gm8234 predicted gene 8234 | 0.19 | 0.18 | 0.38 | 0.17 | 0.94 | 2.10 |
| Snord98 | Snord98 small nucleolar RNA, C/D box 98 | 0.06 | 0.18 | 0.38 | 0.20 | 3.30 | 2.10 |
| Morn3 | Morn3 MORN repeat containing 3 | 0.78 | 0.18 | 0.38 | 0.23 | 0.24 | 2.10 |
| Gm10865 | Gm10865 predicted gene 10865 | 0.42 | 0.18 | 0.38 | 0.55 | 0.44 | 2.10 |
| Gm16712 | Gm16712 predicted gene, 16712 | 0.28 | 0.18 | 0.38 | 0.75 | 0.66 | 2.10 |
| Chil3 | Chil3 chitinase-like 3 | 0.08 | 0.37 | 0.77 | 0.81 | 4.39 | 2.10 |
| Rbm48 | Rbm48 RNA binding motif protein 48 | 5.99 | 5.53 | 11.60 | 8.86 | 0.92 | 2.10 |
| Rnf169 | Rnf169 ring finger protein 169 | 33.26 | 33.16 | 69.51 | 51.32 | 1.00 | 2.10 |
| Slc12a2 | Slc12a2 solute carrier family 12, member 2 | 73.63 | 45.58 | 95.53 | 60.33 | 0.62 | 2.10 |
| Dicer1 | Dicer1 dicer 1, ribonuclease type III | 56.06 | 52.68 | 110.30 | 79.46 | 0.94 | 2.09 |
| Calml4 | Calml4 calmodulin-like 4 | 6.26 | 1.15 | 2.40 | 1.81 | 0.18 | 2.09 |
| Mir1898 | Mir1898 microRNA 1898 | 0.47 | 0.39 | 0.82 | 0.40 | 0.83 | 2.09 |
| Hnf1a | Hnf1a HNF1 homeobox A | 0.61 | 0.39 | 0.82 | 0.55 | 0.64 | 2.09 |
| Mob3c | Mob3c MOB kinase activator 3C | 4.05 | 4.33 | 9.05 | 7.57 | 1.07 | 2.09 |
| Ube2j2 | Ube2j2 ubiquitin-conjugating enzyme E2J 2 | 1.94 | 2.40 | 5.01 | 3.51 | 1.24 | 2.09 |
| Zfp24 | Zfp24 zinc finger protein 24 | 26.74 | 24.42 | 50.96 | 36.48 | 0.91 | 2.09 |
| 0610039K10Rik | 0610039K10Rik RIKEN cDNA 0610039K10 gene | 0.08 | 0.21 | 0.43 | 0.06 | 2.51 | 2.08 |
| Rec114 | Rec114 REC114 meiotic recombination protein | 0.36 | 0.42 | 0.87 | 0.35 | 1.16 | 2.08 |
| Birc7 | Birc7 baculoviral IAP repeat-containing 7 (livin) | 1.25 | 0.42 | 0.87 | 0.58 | 0.33 | 2.08 |
| Kif14 | Kif14 kinesin family member 14 | 0.47 | 0.21 | 0.43 | 0.63 | 0.44 | 2.08 |
| Bmp5 | Bmp5 bone morphogenetic protein 5 | 0.83 | 0.63 | 1.30 | 1.38 | 0.75 | 2.08 |
| Erbin | Erbin Erbb2 interacting protein | 66.04 | 56.59 | 117.41 | 81.70 | 0.86 | 2.07 |
| Krt8 | Krt8 keratin 8 | 3.80 | 1.33 | 2.76 | 1.87 | 0.35 | 2.07 |
| Lpin3 | Lpin3 lipin 3 | 1.52 | 2.95 | 6.11 | 5.70 | 1.93 | 2.07 |
| Bcl2l11 | Bcl2l11 BCL2-like 11 (apoptosis facilitator) | 6.60 | 4.54 | 9.40 | 6.76 | 0.69 | 2.07 |
| Tet2 | Tet2 tet methylcytosine dioxygenase 2 | 29.60 | 31.60 | 65.42 | 52.07 | 1.07 | 2.07 |
| Pkdrej | Pkdrej polycystin (PKD) family receptor for egg jelly | 1.22 | 1.15 | 2.38 | 2.96 | 0.94 | 2.07 |
| Itpripl1 | Itpripl1 inositol 1,4,5-triphosphate receptor interacting protein-like 1 | 4.35 | 2.64 | 5.44 | 3.16 | 0.61 | 2.07 |
| Vsig2 | Vsig2 V-set and immunoglobulin domain containing 2 | 7.57 | 9.47 | 19.55 | 14.21 | 1.25 | 2.06 |
| Lrp12 | Lrp12 low density lipoprotein-related protein 12 | 48.64 | 40.60 | 83.80 | 65.88 | 0.83 | 2.06 |
| Tnpo1 | Tnpo1 transportin 1 | 39.41 | 32.88 | 67.85 | 43.04 | 0.83 | 2.06 |
| Rad51ap1 | Rad51ap1 RAD51 associated protein 1 | 1.11 | 1.23 | 2.53 | 2.53 | 1.11 | 2.06 |
| Slc7a2 | Slc7a2 solute carrier family 7 (cationic amino acid transporter, y+ system), member 2 | 34.14 | 24.55 | 50.58 | 37.54 | 0.72 | 2.06 |
| Zc3h12a | Zc3h12a zinc finger CCCH type containing 12A | 1.77 | 3.81 | 7.85 | 5.98 | 2.15 | 2.06 |
| Bmp4 | Bmp4 bone morphogenetic protein 4 | 2.33 | 1.02 | 2.10 | 1.38 | 0.44 | 2.06 |
| Abca1 | Abca1 ATP-binding cassette, sub-family A (ABC1), member 1 | 42.32 | 50.04 | 103.04 | 80.50 | 1.18 | 2.06 |
| Pnldc1 | Pnldc1 poly(A)-specific ribonuclease (PARN)-like domain containing 1 | 1.25 | 0.78 | 1.61 | 0.75 | 0.63 | 2.06 |
| 1700125H20Rik | 1700125H20Rik RIKEN cDNA 1700125H20 gene | 0.25 | 0.26 | 0.54 | 0.83 | 1.05 | 2.06 |
| Pdgfrl | Pdgfrl platelet-derived growth factor receptor-like | 1.64 | 0.78 | 1.61 | 0.98 | 0.48 | 2.06 |
| Wdr26 | Wdr26 WD repeat domain 26 | 113.95 | 99.91 | 205.37 | 160.36 | 0.88 | 2.06 |
| Cep57 | Cep57 centrosomal protein 57 | 13.22 | 11.64 | 23.92 | 17.23 | 0.88 | 2.06 |
| Pik3c2a | Pik3c2a phosphatidylinositol-4-phosphate 3-kinase catalytic subunit type 2 alpha | 27.63 | 24.60 | 50.58 | 41.17 | 0.89 | 2.06 |
| Abl2 | Abl2 v-abl Abelson murine leukemia viral oncogene 2 (arg, Abelson-related gene) | 87.35 | 77.57 | 159.40 | 110.21 | 0.89 | 2.05 |
| Top3a | Top3a topoisomerase (DNA) III alpha | 7.09 | 7.25 | 14.90 | 14.30 | 1.02 | 2.05 |
| Nifk | Nifk nucleolar protein interacting with the FHA domain of MKI67 | 20.95 | 16.15 | 33.17 | 23.91 | 0.77 | 2.05 |
| Sfmbt1 | Sfmbt1 Scm-like with four mbt domains 1 | 29.51 | 25.73 | 52.72 | 36.13 | 0.87 | 2.05 |
| Krt80 | Krt80 keratin 80 | 0.42 | 0.29 | 0.59 | 0.63 | 0.69 | 2.05 |
| Lamc1 | Lamc1 laminin, gamma 1 | 40.29 | 37.81 | 77.41 | 59.55 | 0.94 | 2.05 |
| Mgea5 | Mgea5 meningioma expressed antigen 5 (hyaluronidase) | 163.86 | 122.40 | 250.56 | 162.98 | 0.75 | 2.05 |
| Timeless | Timeless timeless circadian clock 1 | 3.44 | 4.67 | 9.56 | 8.14 | 1.36 | 2.05 |
| Nhlh2 | Nhlh2 nescient helix loop helix 2 | 1.30 | 1.17 | 2.40 | 2.24 | 0.90 | 2.05 |
| Traf6 | Traf6 TNF receptor-associated factor 6 | 19.62 | 17.66 | 36.11 | 23.88 | 0.90 | 2.04 |
| Ctnna3 | Ctnna3 catenin (cadherin associated protein), alpha 3 | 1.14 | 0.60 | 1.23 | 0.52 | 0.53 | 2.04 |
| Twf1 | Twf1 twinfilin actin binding protein 1 | 40.29 | 37.60 | 76.85 | 57.28 | 0.93 | 2.04 |
| Tdp2 | Tdp2 tyrosyl-DNA phosphodiesterase 2 | 11.75 | 9.78 | 19.99 | 16.63 | 0.83 | 2.04 |
| Tead4 | Tead4 TEA domain family member 4 | 0.75 | 1.85 | 3.78 | 3.19 | 2.48 | 2.04 |
| Rbm46 | Rbm46 RNA binding motif protein 46 | 0.47 | 0.31 | 0.64 | 0.83 | 0.66 | 2.04 |
| Nedd9 | Nedd9 neural precursor cell expressed, developmentally down-regulated gene 9 | 9.92 | 9.97 | 20.29 | 15.25 | 1.00 | 2.04 |
| Cwf19l2 | Cwf19l2 CWF19-like 2, cell cycle control (S. pombe) | 7.84 | 8.04 | 16.36 | 13.90 | 1.02 | 2.04 |
| Klf11 | Klf11 Kruppel-like factor 11 | 5.29 | 4.70 | 9.56 | 6.33 | 0.89 | 2.04 |
| Dapk1 | Dapk1 death associated protein kinase 1 | 136.35 | 153.71 | 312.79 | 242.84 | 1.13 | 2.03 |
| Hs3st3a1 | Hs3st3a1 heparan sulfate (glucosamine) 3-O-sulfotransferase 3A1 | 0.67 | 0.34 | 0.69 | 0.46 | 0.51 | 2.03 |
| Dpp4 | Dpp4 dipeptidylpeptidase 4 | 1.47 | 0.68 | 1.38 | 1.44 | 0.46 | 2.03 |
| Srsf3 | Srsf3 serine/arginine-rich splicing factor 3 | 79.84 | 62.12 | 126.35 | 99.91 | 0.78 | 2.03 |
| Rbm12 | Rbm12 RNA binding motif protein 12 | 15.99 | 15.26 | 31.00 | 14.82 | 0.95 | 2.03 |
| Zfp26 | Zfp26 zinc finger protein 26 | 20.48 | 18.39 | 37.34 | 26.47 | 0.90 | 2.03 |
| **Igf2** | **Igf2 insulin-like growth factor 2** | **134.99** | **44.88** | **91.08** | **76.64** | **0.33** | **2.03** |
| Rnf17 | Rnf17 ring finger protein 17 | 0.61 | 0.37 | 0.74 | 0.17 | 0.60 | 2.03 |
| Mx1 | Mx1 MX dynamin-like GTPase 1 | 0.17 | 1.10 | 2.22 | 1.21 | 6.59 | 2.03 |
| Mtmr2 | Mtmr2 myotubularin related protein 2 | 35.11 | 30.37 | 61.54 | 55.81 | 0.86 | 2.03 |
| Wdr43 | Wdr43 WD repeat domain 43 | 19.70 | 17.69 | 35.83 | 24.65 | 0.90 | 2.03 |
| Col2a1 | Col2a1 collagen, type II, alpha 1 | 1.91 | 1.17 | 2.38 | 2.30 | 0.61 | 2.02 |
| Fgf7 | Fgf7 fibroblast growth factor 7 | 0.55 | 0.39 | 0.79 | 0.66 | 0.71 | 2.02 |
| Rab11fip1 | Rab11fip1 RAB11 family interacting protein 1 (class I) | 4.27 | 1.98 | 4.01 | 2.30 | 0.46 | 2.02 |
| Ncaph | Ncaph non-SMC condensin I complex, subunit H | 0.78 | 0.81 | 1.64 | 1.32 | 1.04 | 2.02 |
| Zfp354c | Zfp354c zinc finger protein 354C | 39.07 | 34.91 | 70.56 | 59.41 | 0.89 | 2.02 |
| Foxc2 | Foxc2 forkhead box C2 | 0.78 | 0.42 | 0.84 | 0.81 | 0.54 | 2.02 |
| Nufip2 | Nufip2 nuclear fragile X mental retardation protein interacting protein 2 | 48.03 | 48.17 | 97.27 | 66.54 | 1.00 | 2.02 |
| Rbsn | Rbsn rabenosyn, RAB effector | 43.31 | 37.94 | 76.52 | 54.98 | 0.88 | 2.02 |
| Impad1 | Impad1 inositol monophosphatase domain containing 1 | 59.86 | 48.19 | 97.19 | 73.85 | 0.81 | 2.02 |
| Nup107 | Nup107 nucleoporin 107 | 12.89 | 10.20 | 20.57 | 16.05 | 0.79 | 2.02 |
| Plekhg6 | Plekhg6 pleckstrin homology domain containing, family G (with RhoGef domain) member 6 | 0.47 | 0.44 | 0.89 | 0.92 | 0.94 | 2.02 |
| Sfrp1 | Sfrp1 secreted frizzled-related protein 1 | 5.99 | 3.57 | 7.21 | 6.99 | 0.60 | 2.02 |
| Akap2 | Akap2 A kinase (PRKA) anchor protein 2 | 71.86 | 67.39 | 135.83 | 110.07 | 0.94 | 2.02 |
| Phf11c | Phf11c PHD finger protein 11C | 0.42 | 0.47 | 0.95 | 0.89 | 1.13 | 2.01 |
| Fgf23 | Fgf23 fibroblast growth factor 23 | 0.55 | 0.47 | 0.95 | 1.18 | 0.85 | 2.01 |
| Arntl | Arntl aryl hydrocarbon receptor nuclear translocator-like | 20.45 | 20.40 | 41.07 | 26.73 | 1.00 | 2.01 |
| Dtwd2 | Dtwd2 DTW domain containing 2 | 2.58 | 1.90 | 3.83 | 3.05 | 0.74 | 2.01 |
| Kdm3a | Kdm3a lysine (K)-specific demethylase 3A | 29.98 | 29.90 | 60.13 | 51.73 | 1.00 | 2.01 |
| Cd2ap | Cd2ap CD2-associated protein | 19.95 | 18.21 | 36.62 | 24.34 | 0.91 | 2.01 |
| Snord53 | Snord53 small nucleolar RNA, C/D box 53 | 0.42 | 0.50 | 1.00 | 0.43 | 1.19 | 2.01 |
| Zfp433 | Zfp433 zinc finger protein 433 | 0.91 | 0.50 | 1.00 | 1.04 | 0.54 | 2.01 |
| Chrdl1 | Chrdl1 chordin-like 1 | 4.43 | 2.06 | 4.14 | 3.16 | 0.46 | 2.01 |
| Trim24 | Trim24 tripartite motif-containing 24 | 23.20 | 19.10 | 38.33 | 29.34 | 0.82 | 2.01 |
| Idi1 | Idi1 isopentenyl-diphosphate delta isomerase | 10.53 | 4.80 | 9.63 | 8.57 | 0.46 | 2.01 |
| Cep152 | Cep152 centrosomal protein 152 | 3.21 | 3.24 | 6.49 | 5.44 | 1.01 | 2.01 |
| Fap | Fap fibroblast activation protein | 1.39 | 0.55 | 1.10 | 0.83 | 0.40 | 2.01 |
| Rbak | Rbak RB-associated KRAB zinc finger | 10.61 | 9.52 | 19.09 | 13.72 | 0.90 | 2.00 |
| Gmeb1 | Gmeb1 glucocorticoid modulatory element binding protein 1 | 17.49 | 14.35 | 28.73 | 24.63 | 0.82 | 2.00 |
| Elmsan1 | Elmsan1 ELM2 and Myb/SANT-like domain containing 1 | 33.89 | 36.58 | 73.22 | 51.93 | 1.08 | 2.00 |
| Riok3 | Riok3 RIO kinase 3 | 44.76 | 41.20 | 82.42 | 65.48 | 0.92 | 2.00 |
| Lrrc24 | Lrrc24 leucine rich repeat containing 24 | 60.80 | 60.51 | 30.23 | 37.89 | 1.00 | 0.50 |
| Fam198b | Fam198b family with sequence similarity 198, member B | 3.77 | 3.89 | 1.94 | 2.01 | 1.03 | 0.50 |
| Best1 | Best1 bestrophin 1 | 1.30 | 1.33 | 0.66 | 0.52 | 1.02 | 0.50 |
| Zfp133-ps | Zfp133-ps zinc finger protein 133, pseudogene | 2.47 | 1.38 | 0.69 | 0.55 | 0.56 | 0.50 |
| Cbfa2t3 | Cbfa2t3 core-binding factor, runt domain, alpha subunit 2, translocated to, 3 (human) | 39.46 | 32.17 | 16.05 | 18.01 | 0.82 | 0.50 |
| Acp6 | Acp6 acid phosphatase 6, lysophosphatidic | 14.19 | 13.80 | 6.87 | 6.99 | 0.97 | 0.50 |
| C030018K13Rik | C030018K13Rik RIKEN cDNA C030018K13 gene | 2.99 | 1.54 | 0.77 | 0.32 | 0.51 | 0.50 |
| Nxph2 | Nxph2 neurexophilin 2 | 1.58 | 1.54 | 0.77 | 0.78 | 0.97 | 0.50 |
| 2010315B03Rik | 2010315B03Rik RIKEN cDNA 2010315B03 gene | 10.50 | 9.29 | 4.63 | 6.16 | 0.88 | 0.50 |
| F8 | F8 coagulation factor VIII | 1.11 | 1.64 | 0.82 | 0.86 | 1.48 | 0.50 |
| Alkbh2 | Alkbh2 alkB homolog 2, alpha-ketoglutarate-dependent dioxygenase | 5.63 | 6.63 | 3.30 | 4.23 | 1.18 | 0.50 |
| Gna15 | Gna15 guanine nucleotide binding protein, alpha 15 | 3.46 | 5.09 | 2.53 | 3.39 | 1.47 | 0.50 |
| Rpgrip1l | Rpgrip1l Rpgrip1-like | 18.65 | 16.93 | 8.41 | 10.27 | 0.91 | 0.50 |
| Mrpl36 | Mrpl36 mitochondrial ribosomal protein L36 | 24.50 | 19.10 | 9.48 | 10.44 | 0.78 | 0.50 |
| Tmem74b | Tmem74b transmembrane protein 74B | 4.18 | 3.91 | 1.94 | 1.81 | 0.94 | 0.50 |
| Fam208a | Fam208a family with sequence similarity 208, member A | 35.36 | 34.26 | 17.00 | 23.59 | 0.97 | 0.50 |
| Nmbr | Nmbr neuromedin B receptor | 1.97 | 2.11 | 1.05 | 1.90 | 1.07 | 0.50 |
| Zfp558 | Zfp558 zinc finger protein 558 | 2.22 | 2.17 | 1.07 | 1.47 | 0.98 | 0.50 |
| Lgi4 | Lgi4 leucine-rich repeat LGI family, member 4 | 82.81 | 92.47 | 45.80 | 45.77 | 1.12 | 0.50 |
| Amdhd2 | Amdhd2 amidohydrolase domain containing 2 | 29.68 | 32.48 | 16.07 | 21.35 | 1.09 | 0.49 |
| Ethe1 | Ethe1 ethylmalonic encephalopathy 1 | 12.47 | 15.08 | 7.46 | 9.58 | 1.21 | 0.49 |
| Eef1akmt1 | Eef1akmt1 EEF1A alpha lysine methyltransferase 1 | 33.95 | 30.37 | 15.03 | 19.22 | 0.89 | 0.49 |
| Mettl26 | Mettl26 methyltransferase like 26 | 42.04 | 40.08 | 19.81 | 24.77 | 0.95 | 0.49 |
| Gm266 | Gm266 predicted gene 266 | 3.63 | 3.05 | 1.51 | 2.07 | 0.84 | 0.49 |
| Zfp760 | Zfp760 zinc finger protein 760 | 6.54 | 6.21 | 3.07 | 4.69 | 0.95 | 0.49 |
| Ntn3 | Ntn3 netrin 3 | 22.92 | 22.36 | 11.04 | 12.66 | 0.98 | 0.49 |
| Pot1a | Pot1a protection of telomeres 1A | 13.44 | 11.40 | 5.62 | 7.68 | 0.85 | 0.49 |
| Gtf2h4 | Gtf2h4 general transcription factor II H, polypeptide 4 | 26.19 | 29.54 | 14.54 | 22.96 | 1.13 | 0.49 |
| Josd2 | Josd2 Josephin domain containing 2 | 54.62 | 40.91 | 20.14 | 17.15 | 0.75 | 0.49 |
| Ankrd63 | Ankrd63 ankyrin repeat domain 63 | 26.49 | 27.68 | 13.62 | 14.56 | 1.04 | 0.49 |
| Kitl | Kitl kit ligand | 20.81 | 20.06 | 9.86 | 10.24 | 0.96 | 0.49 |
| Nwd2 | Nwd2 NACHT and WD repeat domain containing 2 | 31.20 | 25.07 | 12.29 | 14.76 | 0.80 | 0.49 |
| Gm13498 | Gm13498 predicted gene 13498 | 0.03 | 0.10 | 0.05 | 0.03 | 3.77 | 0.49 |
| Kncn | Kncn kinocilin | 0.03 | 0.10 | 0.05 | 0.03 | 3.77 | 0.49 |
| Rab19 | Rab19 RAB19, member RAS oncogene family | 0.03 | 0.10 | 0.05 | 0.03 | 3.77 | 0.49 |
| B230112J18Rik | B230112J18Rik RIKEN cDNA B230112J18 gene | 0.06 | 0.10 | 0.05 | 0.03 | 1.88 | 0.49 |
| Sfta3-ps | Sfta3-ps surfactant associated 3, pseudogene | 0.06 | 0.10 | 0.05 | 0.03 | 1.88 | 0.49 |
| Olfr461 | Olfr461 olfactory receptor 461 | 0.08 | 0.10 | 0.05 | 0.03 | 1.26 | 0.49 |
| E330017L17Rik | E330017L17Rik RIKEN cDNA E330017L17 gene | 0.11 | 0.10 | 0.05 | 0.03 | 0.94 | 0.49 |
| Nphs1os | Nphs1os nephrosis 1 homolog, nephrin, opposite strand | 0.11 | 0.10 | 0.05 | 0.03 | 0.94 | 0.49 |
| Lpar3 | Lpar3 lysophosphatidic acid receptor 3 | 0.14 | 0.21 | 0.10 | 0.03 | 1.51 | 0.49 |
| Allc | Allc allantoicase | 0.17 | 0.10 | 0.05 | 0.03 | 0.63 | 0.49 |
| Tmem51os1 | Tmem51os1 Tmem51 opposite strand 1 | 0.22 | 0.21 | 0.10 | 0.03 | 0.94 | 0.49 |
| Snord35b | Snord35b small nucleolar RNA, C/D box 35B | 0.75 | 0.21 | 0.10 | 0.03 | 0.28 | 0.49 |
| Hephl1 | Hephl1 hephaestin-like 1 | 0.03 | 0.10 | 0.05 | 0.06 | 3.77 | 0.49 |
| 1700092K14Rik | 1700092K14Rik RIKEN cDNA 1700092K14 gene | 0.03 | 0.10 | 0.05 | 0.06 | 3.77 | 0.49 |
| Slc7a15 | Slc7a15 solute carrier family 7 (cationic amino acid transporter, y+ system), member 15 | 0.06 | 0.10 | 0.05 | 0.06 | 1.88 | 0.49 |
| Gpr171 | Gpr171 G protein-coupled receptor 171 | 0.08 | 0.10 | 0.05 | 0.06 | 1.26 | 0.49 |
| Areg | Areg amphiregulin | 0.08 | 0.10 | 0.05 | 0.06 | 1.26 | 0.49 |
| Gm10653 | Gm10653 predicted gene 10653 | 0.08 | 0.10 | 0.05 | 0.06 | 1.26 | 0.49 |
| Mov10l1 | Mov10l1 Moloney leukemia virus 10-like 1 | 0.08 | 0.10 | 0.05 | 0.06 | 1.26 | 0.49 |
| Mir688 | Mir688 microRNA 688 | 0.17 | 0.21 | 0.10 | 0.06 | 1.26 | 0.49 |
| Nr1h4 | Nr1h4 nuclear receptor subfamily 1, group H, member 4 | 0.33 | 0.10 | 0.05 | 0.06 | 0.31 | 0.49 |
| Ccdc158 | Ccdc158 coiled-coil domain containing 158 | 0.36 | 0.21 | 0.10 | 0.06 | 0.58 | 0.49 |
| 1700056E22Rik | 1700056E22Rik RIKEN cDNA 1700056E22 gene | 0.03 | 0.10 | 0.05 | 0.09 | 3.77 | 0.49 |
| Fbxo43 | Fbxo43 F-box protein 43 | 0.08 | 0.21 | 0.10 | 0.09 | 2.51 | 0.49 |
| 4930593C16Rik | 4930593C16Rik RIKEN cDNA 4930593C16 gene | 0.17 | 0.21 | 0.10 | 0.09 | 1.26 | 0.49 |
| Rs1 | Rs1 retinoschisis (X-linked, juvenile) 1 (human) | 0.25 | 0.21 | 0.10 | 0.09 | 0.84 | 0.49 |
| Cyp2e1 | Cyp2e1 cytochrome P450, family 2, subfamily e, polypeptide 1 | 0.30 | 0.31 | 0.15 | 0.09 | 1.03 | 0.49 |
| Rhbdl2 | Rhbdl2 rhomboid like 2 | 0.47 | 0.42 | 0.20 | 0.09 | 0.89 | 0.49 |
| D630024D03Rik | D630024D03Rik RIKEN cDNA D630024D03 gene | 0.53 | 0.31 | 0.15 | 0.09 | 0.59 | 0.49 |
| Scn10a | Scn10a sodium channel, voltage-gated, type X, alpha | 0.03 | 0.42 | 0.20 | 0.12 | 15.06 | 0.49 |
| Snord68 | Snord68 small nucleolar RNA, C/D box 68 | 0.08 | 0.10 | 0.05 | 0.12 | 1.26 | 0.49 |
| Snora62 | Snora62 small nucleolar RNA, H/ACA box 62 | 0.19 | 0.10 | 0.05 | 0.12 | 0.54 | 0.49 |
| Abca12 | Abca12 ATP-binding cassette, sub-family A (ABC1), member 12 | 0.30 | 0.10 | 0.05 | 0.12 | 0.34 | 0.49 |
| Ttc30a2 | Ttc30a2 tetratricopeptide repeat domain 30A2 | 0.08 | 0.10 | 0.05 | 0.14 | 1.26 | 0.49 |
| Gm8787 | Gm8787 predicted gene 8787 | 0.30 | 0.21 | 0.10 | 0.14 | 0.68 | 0.49 |
| Gsx1 | Gsx1 GS homeobox 1 | 0.42 | 0.42 | 0.20 | 0.14 | 1.00 | 0.49 |
| Ifi205 | Ifi205 interferon activated gene 205 | 0.03 | 0.10 | 0.05 | 0.17 | 3.77 | 0.49 |
| Gm13034 | Gm13034 predicted gene 13034 | 0.06 | 0.10 | 0.05 | 0.17 | 1.88 | 0.49 |
| Gm10190 | Gm10190 predicted gene 10190 | 0.17 | 0.21 | 0.10 | 0.17 | 1.26 | 0.49 |
| 4833427G06Rik | 4833427G06Rik RIKEN cDNA 4833427G06 gene | 0.42 | 0.31 | 0.15 | 0.17 | 0.75 | 0.49 |
| Ercc6l | Ercc6l excision repair cross-complementing rodent repair deficiency complementation group 6 like | 0.03 | 0.10 | 0.05 | 0.20 | 3.77 | 0.49 |
| Ptcra | Ptcra pre T cell antigen receptor alpha | 0.11 | 0.10 | 0.05 | 0.20 | 0.94 | 0.49 |
| Gm6588 | Gm6588 predicted gene 6588 | 0.19 | 0.42 | 0.20 | 0.20 | 2.15 | 0.49 |
| Gm14393 | Gm14393 predicted gene 14393 | 0.28 | 0.21 | 0.10 | 0.23 | 0.75 | 0.49 |
| Scnn1g | Scnn1g sodium channel, nonvoltage-gated 1 gamma | 0.30 | 0.10 | 0.05 | 0.23 | 0.34 | 0.49 |
| Tsga13 | Tsga13 testis specific gene A13 | 0.08 | 0.10 | 0.05 | 0.29 | 1.26 | 0.49 |
| Ccdc105 | Ccdc105 coiled-coil domain containing 105 | 0.55 | 0.42 | 0.20 | 0.29 | 0.75 | 0.49 |
| Cfap57 | Cfap57 cilia and flagella associated protein 57 | 1.33 | 0.31 | 0.15 | 0.29 | 0.24 | 0.49 |
| Dnd1 | Dnd1 DND microRNA-mediated repression inhibitor 1 | 1.39 | 1.51 | 0.74 | 0.63 | 1.09 | 0.49 |
| Tcte2 | Tcte2 t-complex-associated testis expressed 2 | 1.72 | 1.51 | 0.74 | 0.75 | 0.88 | 0.49 |
| Atp7b | Atp7b ATPase, Cu++ transporting, beta polypeptide | 2.77 | 2.56 | 1.25 | 1.35 | 0.92 | 0.49 |
| 3110082J24Rik | 3110082J24Rik RIKEN cDNA 3110082J24 gene | 1.44 | 0.83 | 0.41 | 0.86 | 0.58 | 0.49 |
| Nme4 | Nme4 NME/NM23 nucleoside diphosphate kinase 4 | 6.65 | 6.37 | 3.12 | 5.44 | 0.96 | 0.49 |
| Platr26 | Platr26 pluripotency associated transcript 26 | 0.03 | 0.05 | 0.03 | 0.03 | 1.88 | 0.49 |
| Gm13212 | Gm13212 predicted gene 13212 | 0.03 | 0.05 | 0.03 | 0.03 | 1.88 | 0.49 |
| Slfnl1 | Slfnl1 schlafen like 1 | 0.03 | 0.05 | 0.03 | 0.03 | 1.88 | 0.49 |
| Ovol3 | Ovol3 ovo like zinc finger 3 | 0.03 | 0.05 | 0.03 | 0.03 | 1.88 | 0.49 |
| 1700047A11Rik | 1700047A11Rik RIKEN cDNA 1700047A11 gene | 0.03 | 0.05 | 0.03 | 0.03 | 1.88 | 0.49 |
| 4932413F04Rik | 4932413F04Rik RIKEN cDNA 4932413F04 gene | 0.03 | 0.05 | 0.03 | 0.03 | 1.88 | 0.49 |
| Nepn | Nepn nephrocan | 0.03 | 0.05 | 0.03 | 0.03 | 1.88 | 0.49 |
| Adam28 | Adam28 a disintegrin and metallopeptidase domain 28 | 0.03 | 0.05 | 0.03 | 0.03 | 1.88 | 0.49 |
| Stpg4 | Stpg4 sperm tail PG rich repeat containing 4 | 0.03 | 0.05 | 0.03 | 0.03 | 1.88 | 0.49 |
| A930018P22Rik | A930018P22Rik RIKEN cDNA A930018P22 gene | 0.06 | 0.05 | 0.03 | 0.03 | 0.94 | 0.49 |
| 4930567H12Rik | 4930567H12Rik RIKEN cDNA 4930567H12 gene | 0.06 | 0.05 | 0.03 | 0.03 | 0.94 | 0.49 |
| Iqcf5 | Iqcf5 IQ motif containing F5 | 0.06 | 0.05 | 0.03 | 0.03 | 0.94 | 0.49 |
| 5730435O14Rik | 5730435O14Rik RIKEN cDNA 5730435O14 gene | 0.06 | 0.05 | 0.03 | 0.03 | 0.94 | 0.49 |
| Mir6906 | Mir6906 microRNA 6906 | 0.06 | 0.05 | 0.03 | 0.03 | 0.94 | 0.49 |
| Mir665 | Mir665 microRNA 665 | 0.06 | 0.05 | 0.03 | 0.03 | 0.94 | 0.49 |
| Cyp2d9 | Cyp2d9 cytochrome P450, family 2, subfamily d, polypeptide 9 | 0.06 | 0.05 | 0.03 | 0.03 | 0.94 | 0.49 |
| Mir6984 | Mir6984 microRNA 6984 | 0.06 | 0.05 | 0.03 | 0.03 | 0.94 | 0.49 |
| Myog | Myog myogenin | 0.08 | 0.05 | 0.03 | 0.03 | 0.63 | 0.49 |
| Nat8f2 | Nat8f2 N-acetyltransferase 8 (GCN5-related) family member 2 | 0.08 | 0.05 | 0.03 | 0.03 | 0.63 | 0.49 |
| Dyrk4 | Dyrk4 dual-specificity tyrosine-(Y)-phosphorylation regulated kinase 4 | 0.11 | 0.05 | 0.03 | 0.03 | 0.47 | 0.49 |
| Cyp4f41-ps | Cyp4f41-ps cytochrome P450, family 4, subfamily f, polypeptide 41 pseudogene | 0.11 | 0.05 | 0.03 | 0.03 | 0.47 | 0.49 |
| 4933424G05Rik | 4933424G05Rik RIKEN cDNA 4933424G05 gene | 0.11 | 0.05 | 0.03 | 0.03 | 0.47 | 0.49 |
| Mir154 | Mir154 microRNA 154 | 0.19 | 0.05 | 0.03 | 0.03 | 0.27 | 0.49 |
| Piwil1 | Piwil1 piwi-like RNA-mediated gene silencing 1 | 0.33 | 0.05 | 0.03 | 0.03 | 0.16 | 0.49 |
| Gal3st2c | Gal3st2c galactose-3-O-sulfotransferase 2C | 0.03 | 0.05 | 0.03 | 0.06 | 1.88 | 0.49 |
| Gm5878 | Gm5878 predicted gene 5878 | 0.03 | 0.05 | 0.03 | 0.06 | 1.88 | 0.49 |
| Klk1b27 | Klk1b27 kallikrein 1-related peptidase b27 | 0.03 | 0.05 | 0.03 | 0.06 | 1.88 | 0.49 |
| Ssxb5 | Ssxb5 synovial sarcoma, X member B5 | 0.03 | 0.05 | 0.03 | 0.06 | 1.88 | 0.49 |
| Fam71b | Fam71b family with sequence similarity 71, member B | 0.03 | 0.05 | 0.03 | 0.06 | 1.88 | 0.49 |
| Krt33b | Krt33b keratin 33B | 0.03 | 0.05 | 0.03 | 0.06 | 1.88 | 0.49 |
| Muc20 | Muc20 mucin 20 | 0.03 | 0.05 | 0.03 | 0.06 | 1.88 | 0.49 |
| Tgm5 | Tgm5 transglutaminase 5 | 0.06 | 0.05 | 0.03 | 0.06 | 0.94 | 0.49 |
| Lmx1b | Lmx1b LIM homeobox transcription factor 1 beta | 0.08 | 0.05 | 0.03 | 0.06 | 0.63 | 0.49 |
| Olfr53 | Olfr53 olfactory receptor 53 | 0.08 | 0.05 | 0.03 | 0.06 | 0.63 | 0.49 |
| Snai3 | Snai3 snail family zinc finger 3 | 0.08 | 0.05 | 0.03 | 0.06 | 0.63 | 0.49 |
| Mir215 | Mir215 microRNA 215 | 0.11 | 0.05 | 0.03 | 0.06 | 0.47 | 0.49 |
| Epgn | Epgn epithelial mitogen | 0.03 | 0.05 | 0.03 | 0.09 | 1.88 | 0.49 |
| Clec12b | Clec12b C-type lectin domain family 12, member B | 0.03 | 0.05 | 0.03 | 0.09 | 1.88 | 0.49 |
| A930002C04Rik | A930002C04Rik RIKEN cDNA A930002C04 gene | 0.03 | 0.05 | 0.03 | 0.09 | 1.88 | 0.49 |
| Cd3d | Cd3d CD3 antigen, delta polypeptide | 0.06 | 0.05 | 0.03 | 0.09 | 0.94 | 0.49 |
| Cpb2 | Cpb2 carboxypeptidase B2 (plasma) | 0.08 | 0.05 | 0.03 | 0.09 | 0.63 | 0.49 |
| Gm833 | Gm833 predicted gene 833 | 0.03 | 0.05 | 0.03 | 0.12 | 1.88 | 0.49 |
| Bpifb6 | Bpifb6 BPI fold containing family B, member 6 | 0.06 | 0.05 | 0.03 | 0.12 | 0.94 | 0.49 |
| Hist1h3e | Hist1h3e histone cluster 1, H3e | 0.06 | 0.05 | 0.03 | 0.12 | 0.94 | 0.49 |
| Tomm20l | Tomm20l translocase of outer mitochondrial membrane 20-like | 0.08 | 0.05 | 0.03 | 0.12 | 0.63 | 0.49 |
| A4gnt | A4gnt alpha-1,4-N-acetylglucosaminyltransferase | 0.11 | 0.05 | 0.03 | 0.12 | 0.47 | 0.49 |
| Glyat | Glyat glycine-N-acyltransferase | 0.17 | 0.05 | 0.03 | 0.12 | 0.31 | 0.49 |
| Ctla4 | Ctla4 cytotoxic T-lymphocyte-associated protein 4 | 0.03 | 0.05 | 0.03 | 0.14 | 1.88 | 0.49 |
| Bpifb4 | Bpifb4 BPI fold containing family B, member 4 | 0.03 | 0.05 | 0.03 | 0.14 | 1.88 | 0.49 |
| H60b | H60b histocompatibility 60b | 0.06 | 0.05 | 0.03 | 0.14 | 0.94 | 0.49 |
| Hist2h4 | Hist2h4 histone cluster 2, H4 | 0.14 | 0.05 | 0.03 | 0.14 | 0.38 | 0.49 |
| Piwil4 | Piwil4 piwi-like RNA-mediated gene silencing 4 | 0.06 | 0.05 | 0.03 | 0.17 | 0.94 | 0.49 |
| Btn1a1 | Btn1a1 butyrophilin, subfamily 1, member A1 | 0.06 | 0.05 | 0.03 | 0.17 | 0.94 | 0.49 |
| Zfp781 | Zfp781 zinc finger protein 781 | 1.36 | 0.89 | 0.43 | 0.46 | 0.65 | 0.49 |
| 4930426L09Rik | 4930426L09Rik RIKEN cDNA 4930426L09 gene | 0.72 | 0.89 | 0.43 | 1.12 | 1.23 | 0.49 |
| Rem1 | Rem1 rad and gem related GTP binding protein 1 | 0.55 | 0.52 | 0.26 | 0.23 | 0.94 | 0.49 |
| Atp8b3 | Atp8b3 ATPase, class I, type 8B, member 3 | 0.53 | 0.52 | 0.26 | 0.35 | 0.99 | 0.49 |
| D330041H03Rik | D330041H03Rik RIKEN cDNA D330041H03 gene | 0.78 | 0.78 | 0.38 | 0.89 | 1.01 | 0.49 |
| Lyzl4 | Lyzl4 lysozyme-like 4 | 0.72 | 0.68 | 0.33 | 0.40 | 0.94 | 0.49 |
| Coa6 | Coa6 cytochrome c oxidase assembly factor 6 | 10.17 | 9.91 | 4.86 | 5.93 | 0.97 | 0.49 |
| Mnd1 | Mnd1 meiotic nuclear divisions 1 | 0.55 | 0.57 | 0.28 | 0.29 | 1.04 | 0.49 |
| Kif20b | Kif20b kinesin family member 20B | 0.36 | 0.57 | 0.28 | 0.40 | 1.59 | 0.49 |
| Erp27 | Erp27 endoplasmic reticulum protein 27 | 0.22 | 0.57 | 0.28 | 0.52 | 2.59 | 0.49 |
| Hcst | Hcst hematopoietic cell signal transducer | 0.47 | 0.99 | 0.49 | 0.58 | 2.10 | 0.49 |
| D630033O11Rik | D630033O11Rik RIKEN cDNA D630033O11 gene | 0.55 | 1.15 | 0.56 | 0.72 | 2.07 | 0.49 |
| Chst5 | Chst5 carbohydrate (N-acetylglucosamine 6-O) sulfotransferase 5 | 1.58 | 1.15 | 0.56 | 0.75 | 0.73 | 0.49 |
| 9630028B13Rik | 9630028B13Rik RIKEN cDNA 9630028B13 gene | 0.42 | 0.47 | 0.23 | 0.06 | 1.13 | 0.49 |
| Trmt1l | Trmt1l tRNA methyltransferase 1 like | 23.64 | 20.61 | 10.09 | 16.92 | 0.87 | 0.49 |
| Gpat2 | Gpat2 glycerol-3-phosphate acyltransferase 2, mitochondrial | 0.33 | 0.37 | 0.18 | 0.63 | 1.10 | 0.49 |
| Fgf22 | Fgf22 fibroblast growth factor 22 | 1.00 | 1.46 | 0.72 | 1.29 | 1.46 | 0.49 |
| Prr16 | Prr16 proline rich 16 | 3.21 | 4.02 | 1.97 | 2.82 | 1.25 | 0.49 |
| AF357355 | AF357355 snoRNA AF357355 | 0.14 | 0.16 | 0.08 | 0.03 | 1.13 | 0.49 |
| E330020D12Rik | E330020D12Rik Riken cDNA E330020D12 gene | 0.03 | 0.16 | 0.08 | 0.06 | 5.65 | 0.49 |
| D830046C22Rik | D830046C22Rik RIKEN cDNA D830046C22 gene | 0.03 | 0.16 | 0.08 | 0.06 | 5.65 | 0.49 |
| Gm10471 | Gm10471 predicted gene 10471 | 0.33 | 0.16 | 0.08 | 0.06 | 0.47 | 0.49 |
| Apela | Apela apelin receptor early endogenous ligand | 0.11 | 0.16 | 0.08 | 0.09 | 1.41 | 0.49 |
| Tmco5b | Tmco5b transmembrane and coiled-coil domains 5B | 0.14 | 0.16 | 0.08 | 0.09 | 1.13 | 0.49 |
| Hsd11b2 | Hsd11b2 hydroxysteroid 11-beta dehydrogenase 2 | 0.03 | 0.16 | 0.08 | 0.12 | 5.65 | 0.49 |
| AY074887 | AY074887 cDNA sequence AY074887 | 0.03 | 0.16 | 0.08 | 0.14 | 5.65 | 0.49 |
| Hes3 | Hes3 hes family bHLH transcription factor 3 | 0.17 | 0.16 | 0.08 | 0.14 | 0.94 | 0.49 |
| Enpp3 | Enpp3 ectonucleotide pyrophosphatase/phosphodiesterase 3 | 0.17 | 0.16 | 0.08 | 0.14 | 0.94 | 0.49 |
| Kcnk7 | Kcnk7 potassium channel, subfamily K, member 7 | 0.42 | 0.16 | 0.08 | 0.14 | 0.38 | 0.49 |
| Pate4 | Pate4 prostate and testis expressed 4 | 0.08 | 0.16 | 0.08 | 0.17 | 1.88 | 0.49 |
| LOC100861615 | LOC100861615 alpha takusan-like | 0.17 | 0.16 | 0.08 | 0.20 | 0.94 | 0.49 |
| Hepacam2 | Hepacam2 HEPACAM family member 2 | 0.25 | 0.16 | 0.08 | 0.20 | 0.63 | 0.49 |
| Cfap61 | Cfap61 cilia and flagella associated protein 61 | 0.69 | 0.16 | 0.08 | 0.32 | 0.23 | 0.49 |
| Gm16675 | Gm16675 predicted gene, 16675 | 0.19 | 0.16 | 0.08 | 0.37 | 0.81 | 0.49 |
| 4930481A15Rik | 4930481A15Rik RIKEN cDNA 4930481A15 gene | 2.85 | 1.67 | 0.82 | 1.18 | 0.59 | 0.49 |
| Cyb561d2 | Cyb561d2 cytochrome b-561 domain containing 2 | 22.14 | 20.51 | 10.04 | 13.98 | 0.93 | 0.49 |
| Gm6756 | Gm6756 predicted gene 6756 | 0.19 | 0.26 | 0.13 | 0.03 | 1.35 | 0.49 |
| Mir541 | Mir541 microRNA 541 | 0.44 | 0.26 | 0.13 | 0.09 | 0.59 | 0.49 |
| Snord34 | Snord34 small nucleolar RNA, C/D box 34 | 0.14 | 0.26 | 0.13 | 0.12 | 1.88 | 0.49 |
| Ribc2 | Ribc2 RIB43A domain with coiled-coils 2 | 0.75 | 0.26 | 0.13 | 0.12 | 0.35 | 0.49 |
| Tacstd2 | Tacstd2 tumor-associated calcium signal transducer 2 | 0.11 | 0.26 | 0.13 | 0.17 | 2.35 | 0.49 |
| Gm5512 | Gm5512 predicted gene 5512 | 0.39 | 0.26 | 0.13 | 0.17 | 0.67 | 0.49 |
| Ifi27l2b | Ifi27l2b interferon, alpha-inducible protein 27 like 2B | 0.11 | 0.26 | 0.13 | 0.23 | 2.35 | 0.49 |
| Naip6 | Naip6 NLR family, apoptosis inhibitory protein 6 | 0.19 | 0.26 | 0.13 | 0.23 | 1.35 | 0.49 |
| 9330158H04Rik | 9330158H04Rik RIKEN cDNA 9330158H04 gene | 0.42 | 0.26 | 0.13 | 0.23 | 0.63 | 0.49 |
| Cd79a | Cd79a CD79A antigen (immunoglobulin-associated alpha) | 0.44 | 0.26 | 0.13 | 0.23 | 0.59 | 0.49 |
| Ebf2 | Ebf2 early B cell factor 2 | 0.30 | 0.26 | 0.13 | 0.35 | 0.86 | 0.49 |
| Dnmt3l | Dnmt3l DNA (cytosine-5-)-methyltransferase 3-like | 0.42 | 0.26 | 0.13 | 0.40 | 0.63 | 0.49 |
| Ankrd26 | Ankrd26 ankyrin repeat domain 26 | 11.09 | 11.01 | 5.39 | 8.89 | 0.99 | 0.49 |
| Paqr7 | Paqr7 progestin and adipoQ receptor family member VII | 131.97 | 103.79 | 50.81 | 62.17 | 0.79 | 0.49 |
| 2610507I01Rik | 2610507I01Rik RIKEN cDNA 2610507I01 gene | 19.73 | 17.04 | 8.33 | 8.26 | 0.86 | 0.49 |
| Ydjc | Ydjc YdjC homolog (bacterial) | 38.27 | 40.57 | 19.78 | 24.77 | 1.06 | 0.49 |
| Lppos | Lppos LIM domain containing preferred translocation partner in lipoma, opposite strand | 3.52 | 4.77 | 2.33 | 2.19 | 1.36 | 0.49 |
| Tmco6 | Tmco6 transmembrane and coiled-coil domains 6 | 19.34 | 17.61 | 8.54 | 14.47 | 0.91 | 0.48 |
| Iqcc | Iqcc IQ motif containing C | 15.33 | 15.03 | 7.28 | 10.36 | 0.98 | 0.48 |
| 1190007I07Rik | 1190007I07Rik RIKEN cDNA 1190007I07 gene | 6.51 | 6.60 | 3.19 | 3.48 | 1.01 | 0.48 |
| Gpr150 | Gpr150 G protein-coupled receptor 150 | 4.66 | 6.39 | 3.09 | 4.49 | 1.37 | 0.48 |
| Th | Th tyrosine hydroxylase | 1.39 | 2.01 | 0.97 | 1.18 | 1.45 | 0.48 |
| Snord104 | Snord104 small nucleolar RNA, C/D box 104 | 2.77 | 3.81 | 1.84 | 2.91 | 1.37 | 0.48 |
| Saysd1 | Saysd1 SAYSVFN motif domain containing 1 | 16.93 | 16.15 | 7.79 | 15.56 | 0.95 | 0.48 |
| Slc52a3 | Slc52a3 solute carrier protein family 52, member 3 | 3.41 | 5.30 | 2.56 | 3.11 | 1.55 | 0.48 |
| Slc38a11 | Slc38a11 solute carrier family 38, member 11 | 1.83 | 1.75 | 0.84 | 1.15 | 0.96 | 0.48 |
| Pilrb1 | Pilrb1 paired immunoglobin-like type 2 receptor beta 1 | 0.06 | 1.59 | 0.77 | 0.75 | 28.72 | 0.48 |
| Cd244a | Cd244a CD244 molecule A | 0.06 | 1.59 | 0.77 | 0.83 | 28.72 | 0.48 |
| Zfp60 | Zfp60 zinc finger protein 60 | 25.02 | 21.86 | 10.53 | 18.50 | 0.87 | 0.48 |
| Pi16 | Pi16 peptidase inhibitor 16 | 1.50 | 1.54 | 0.74 | 1.01 | 1.03 | 0.48 |
| Shh | Shh sonic hedgehog | 1.39 | 1.54 | 0.74 | 1.06 | 1.11 | 0.48 |
| Gmppb | Gmppb GDP-mannose pyrophosphorylase B | 24.72 | 22.73 | 10.94 | 15.22 | 0.92 | 0.48 |
| Sp6 | Sp6 trans-acting transcription factor 6 | 1.19 | 1.49 | 0.72 | 0.86 | 1.25 | 0.48 |
| Pnma1 | Pnma1 paraneoplastic antigen MA1 | 3.63 | 2.87 | 1.38 | 2.01 | 0.79 | 0.48 |
| Gimap1 | Gimap1 GTPase, IMAP family member 1 | 1.36 | 1.38 | 0.66 | 0.81 | 1.02 | 0.48 |
| Efcab12 | Efcab12 EF-hand calcium binding domain 12 | 6.24 | 5.01 | 2.40 | 2.53 | 0.80 | 0.48 |
| Vamp5 | Vamp5 vesicle-associated membrane protein 5 | 4.27 | 3.68 | 1.76 | 2.19 | 0.86 | 0.48 |
| Gbgt1 | Gbgt1 globoside alpha-1,3-N-acetylgalactosaminyltransferase 1 | 0.69 | 1.23 | 0.59 | 0.89 | 1.77 | 0.48 |
| Ndp | Ndp Norrie disease (pseudoglioma) (human) | 8.37 | 7.64 | 3.65 | 5.96 | 0.91 | 0.48 |
| 1700020D05Rik | 1700020D05Rik RIKEN cDNA 1700020D05 gene | 1.39 | 1.07 | 0.51 | 0.95 | 0.77 | 0.48 |
| Hist1h4h | Hist1h4h histone cluster 1, H4h | 1.61 | 1.02 | 0.49 | 1.09 | 0.63 | 0.48 |
| Dleu2 | Dleu2 deleted in lymphocytic leukemia, 2 | 3.49 | 1.98 | 0.95 | 1.35 | 0.57 | 0.48 |
| Zfp719 | Zfp719 zinc finger protein 719 | 14.60 | 11.64 | 5.55 | 10.18 | 0.80 | 0.48 |
| Hk3 | Hk3 hexokinase 3 | 2.55 | 3.81 | 1.81 | 1.58 | 1.49 | 0.48 |
| Arc | Arc activity regulated cytoskeletal-associated protein | 196.79 | 152.01 | 72.40 | 70.40 | 0.77 | 0.48 |
| Pdia5 | Pdia5 protein disulfide isomerase associated 5 | 2.24 | 1.88 | 0.89 | 0.81 | 0.84 | 0.48 |
| G0s2 | G0s2 G0/G1 switch gene 2 | 6.93 | 6.50 | 3.09 | 4.57 | 0.94 | 0.48 |
| Tmem51 | Tmem51 transmembrane protein 51 | 9.62 | 7.75 | 3.68 | 5.03 | 0.81 | 0.47 |
| Zfp983 | Zfp983 zinc finger protein 983 | 8.65 | 8.30 | 3.94 | 4.66 | 0.96 | 0.47 |
| Stk33 | Stk33 serine/threonine kinase 33 | 1.47 | 1.62 | 0.77 | 0.83 | 1.10 | 0.47 |
| Fam69c | Fam69c family with sequence similarity 69, member C | 11.92 | 10.65 | 5.03 | 5.78 | 0.89 | 0.47 |
| Caskin2 | Caskin2 CASK-interacting protein 2 | 42.23 | 50.80 | 24.02 | 28.19 | 1.20 | 0.47 |
| Atad3aos | Atad3aos ATPase family, AAA domain containing 3A, opposite strand | 0.67 | 0.76 | 0.36 | 0.40 | 1.14 | 0.47 |
| Dnase1l2 | Dnase1l2 deoxyribonuclease 1-like 2 | 3.71 | 2.92 | 1.38 | 1.64 | 0.79 | 0.47 |
| Fam173b | Fam173b family with sequence similarity 173, member B | 6.51 | 5.09 | 2.40 | 2.65 | 0.78 | 0.47 |
| Ano1 | Ano1 anoctamin 1, calcium activated chloride channel | 3.52 | 2.17 | 1.02 | 0.95 | 0.62 | 0.47 |
| Alkbh7 | Alkbh7 alkB homolog 7 | 29.43 | 36.89 | 17.40 | 28.54 | 1.25 | 0.47 |
| Hspa12b | Hspa12b heat shock protein 12B | 11.86 | 13.44 | 6.34 | 5.84 | 1.13 | 0.47 |
| Mcidas | Mcidas multiciliate differentiation and DNA synthesis associated cell cycle protein | 0.61 | 0.70 | 0.33 | 0.49 | 1.16 | 0.47 |
| Gli1 | Gli1 GLI-Kruppel family member GLI1 | 4.07 | 15.50 | 7.31 | 8.08 | 3.80 | 0.47 |
| Prss36 | Prss36 protease, serine 36 | 17.57 | 20.74 | 9.76 | 14.53 | 1.18 | 0.47 |
| BB557941 | BB557941 expressed sequence BB557941 | 0.75 | 0.65 | 0.31 | 0.23 | 0.87 | 0.47 |
| Gm19897 | Gm19897 predicted gene, 19897 | 0.42 | 0.65 | 0.31 | 0.35 | 1.57 | 0.47 |
| Apoa1 | Apoa1 apolipoprotein A-I | 0.44 | 0.65 | 0.31 | 0.46 | 1.47 | 0.47 |
| Slc25a45 | Slc25a45 solute carrier family 25, member 45 | 4.74 | 4.25 | 1.99 | 2.19 | 0.90 | 0.47 |
| Fam71e1 | Fam71e1 family with sequence similarity 71, member E1 | 5.60 | 7.85 | 3.68 | 5.90 | 1.40 | 0.47 |
| Meis1 | Meis1 Meis homeobox 1 | 0.91 | 1.20 | 0.56 | 0.60 | 1.31 | 0.47 |
| Armc2 | Armc2 armadillo repeat containing 2 | 7.04 | 5.30 | 2.48 | 3.34 | 0.75 | 0.47 |
| Oxld1 | Oxld1 oxidoreductase like domain containing 1 | 8.87 | 13.33 | 6.24 | 8.72 | 1.50 | 0.47 |
| Nupr1l | Nupr1l nuclear protein transcriptional regulator 1 like | 0.86 | 1.15 | 0.54 | 0.81 | 1.34 | 0.47 |
| Foxl2 | Foxl2 forkhead box L2 | 1.11 | 1.15 | 0.54 | 0.83 | 1.04 | 0.47 |
| 4930525G20Rik | 4930525G20Rik RIKEN cDNA 4930525G20 gene | 0.97 | 1.70 | 0.79 | 1.27 | 1.75 | 0.47 |
| Ttc30a1 | Ttc30a1 tetratricopeptide repeat domain 30A1 | 3.05 | 2.19 | 1.02 | 1.81 | 0.72 | 0.47 |
| Cd6 | Cd6 CD6 antigen | 0.53 | 0.55 | 0.26 | 0.35 | 1.04 | 0.47 |
| Uts2r | Uts2r urotensin 2 receptor | 0.30 | 0.55 | 0.26 | 0.46 | 1.80 | 0.47 |
| Gipc2 | Gipc2 GIPC PDZ domain containing family, member 2 | 0.42 | 0.55 | 0.26 | 0.55 | 1.32 | 0.47 |
| A330076C08Rik | A330076C08Rik RIKEN cDNA A330076C08 gene | 0.50 | 0.55 | 0.26 | 0.55 | 1.10 | 0.47 |
| Degs2 | Degs2 delta(4)-desaturase, sphingolipid 2 | 8.40 | 10.20 | 4.75 | 4.92 | 1.21 | 0.47 |
| Ccdc8 | Ccdc8 coiled-coil domain containing 8 | 3.57 | 2.58 | 1.20 | 1.50 | 0.72 | 0.47 |
| Itgb3bp | Itgb3bp integrin beta 3 binding protein (beta3-endonexin) | 2.69 | 2.04 | 0.95 | 1.32 | 0.76 | 0.46 |
| D130017N08Rik | D130017N08Rik RIKEN cDNA D130017N08 gene | 8.87 | 7.54 | 3.50 | 3.48 | 0.85 | 0.46 |
| Ankef1 | Ankef1 ankyrin repeat and EF-hand domain containing 1 | 0.94 | 0.50 | 0.23 | 0.37 | 0.53 | 0.46 |
| E030013I19Rik | E030013I19Rik RIKEN cDNA E030013I19 gene | 2.16 | 2.48 | 1.15 | 2.24 | 1.15 | 0.46 |
| Med20 | Med20 mediator complex subunit 20 | 21.45 | 20.04 | 9.25 | 12.34 | 0.93 | 0.46 |
| Leng9 | Leng9 leukocyte receptor cluster (LRC) member 9 | 9.98 | 12.13 | 5.60 | 8.20 | 1.22 | 0.46 |
| Nodal | Nodal nodal | 1.05 | 0.89 | 0.41 | 0.06 | 0.84 | 0.46 |
| Gm10433 | Gm10433 predicted gene 10433 | 0.17 | 0.44 | 0.20 | 0.06 | 2.67 | 0.46 |
| Npw | Npw neuropeptide W | 0.58 | 0.44 | 0.20 | 0.23 | 0.76 | 0.46 |
| Fsip1 | Fsip1 fibrous sheath-interacting protein 1 | 0.55 | 0.44 | 0.20 | 0.29 | 0.80 | 0.46 |
| Ms4a4c | Ms4a4c membrane-spanning 4-domains, subfamily A, member 4C | 0.19 | 0.44 | 0.20 | 0.52 | 2.29 | 0.46 |
| Hes7 | Hes7 hes family bHLH transcription factor 7 | 1.91 | 2.61 | 1.20 | 1.09 | 1.36 | 0.46 |
| Slc25a35 | Slc25a35 solute carrier family 25, member 35 | 6.93 | 6.05 | 2.79 | 3.62 | 0.87 | 0.46 |
| Srbd1 | Srbd1 S1 RNA binding domain 1 | 7.15 | 5.56 | 2.56 | 2.59 | 0.78 | 0.46 |
| A330070K13Rik | A330070K13Rik RIKEN cDNA A330070K13 gene | 2.02 | 1.67 | 0.77 | 1.58 | 0.83 | 0.46 |
| Abcc6 | Abcc6 ATP-binding cassette, sub-family C (CFTR/MRP), member 6 | 2.91 | 1.23 | 0.56 | 0.37 | 0.42 | 0.46 |
| Spats1 | Spats1 spermatogenesis associated, serine-rich 1 | 1.75 | 1.62 | 0.74 | 0.72 | 0.93 | 0.46 |
| Mgmt | Mgmt O-6-methylguanine-DNA methyltransferase | 1.86 | 1.62 | 0.74 | 0.75 | 0.87 | 0.46 |
| Rinl | Rinl Ras and Rab interactor-like | 3.13 | 4.02 | 1.84 | 2.07 | 1.28 | 0.46 |
| Znrd1as | Znrd1as zinc ribbon domain containing 1, antisense | 2.00 | 2.01 | 0.92 | 1.21 | 1.01 | 0.46 |
| Tmem177 | Tmem177 transmembrane protein 177 | 10.17 | 8.32 | 3.81 | 5.29 | 0.82 | 0.46 |
| A630023P12Rik | A630023P12Rik RIKEN cDNA A630023P12 gene | 0.53 | 0.78 | 0.36 | 0.35 | 1.49 | 0.46 |
| Gm10033 | Gm10033 predicted gene 10033 | 2.05 | 1.96 | 0.89 | 1.47 | 0.95 | 0.46 |
| Zfp354b | Zfp354b zinc finger protein 354B | 3.05 | 2.74 | 1.25 | 2.36 | 0.90 | 0.46 |
| Bhlha15 | Bhlha15 basic helix-loop-helix family, member a15 | 0.17 | 0.39 | 0.18 | 0.17 | 2.35 | 0.46 |
| Gm4890 | Gm4890 predicted gene 4890 | 0.67 | 0.39 | 0.18 | 0.20 | 0.59 | 0.46 |
| Trp53cor1 | Trp53cor1 tumor protein p53 pathway corepressor 1 | 0.42 | 0.39 | 0.18 | 0.23 | 0.94 | 0.46 |
| Hist2h2bb | Hist2h2bb histone cluster 2, H2bb | 0.22 | 0.39 | 0.18 | 0.26 | 1.77 | 0.46 |
| Clec10a | Clec10a C-type lectin domain family 10, member A | 0.72 | 0.39 | 0.18 | 0.52 | 0.54 | 0.46 |
| Ptpn18 | Ptpn18 protein tyrosine phosphatase, non-receptor type 18 | 4.13 | 4.25 | 1.94 | 2.42 | 1.03 | 0.46 |
| AW011738 | AW011738 expressed sequence AW011738 | 3.10 | 4.15 | 1.89 | 2.79 | 1.34 | 0.46 |
| 4930578M01Rik | 4930578M01Rik RIKEN cDNA 4930578M01 gene | 1.33 | 1.12 | 0.51 | 0.37 | 0.84 | 0.46 |
| Neat1 | Neat1 nuclear paraspeckle assembly transcript 1 (non-protein coding) | 113.73 | 61.29 | 27.91 | 33.92 | 0.54 | 0.46 |
| Ccdc63 | Ccdc63 coiled-coil domain containing 63 | 0.50 | 0.73 | 0.33 | 0.66 | 1.46 | 0.45 |
| Nme3 | Nme3 NME/NM23 nucleoside diphosphate kinase 3 | 53.04 | 49.91 | 22.69 | 28.16 | 0.94 | 0.45 |
| Slc2a4rg-ps | Slc2a4rg-ps Slc2a4 regulator, pseudogene | 11.81 | 7.83 | 3.55 | 3.91 | 0.66 | 0.45 |
| Zfp862-ps | Zfp862-ps zinc finger protein 862, pseudogene | 16.24 | 13.46 | 6.11 | 9.93 | 0.83 | 0.45 |
| Sox18 | Sox18 SRY (sex determining region Y)-box 18 | 12.47 | 13.54 | 6.13 | 7.19 | 1.09 | 0.45 |
| Kank3 | Kank3 KN motif and ankyrin repeat domains 3 | 28.77 | 30.16 | 13.65 | 19.13 | 1.05 | 0.45 |
| 4933427I22Rik | 4933427I22Rik RIKEN cDNA 4933427I22 gene | 0.47 | 0.68 | 0.31 | 0.40 | 1.44 | 0.45 |
| Tnfsf10 | Tnfsf10 tumor necrosis factor (ligand) superfamily, member 10 | 3.63 | 3.39 | 1.53 | 2.45 | 0.93 | 0.45 |
| Gm1123 | Gm1123 predicted gene 1123 | 0.11 | 0.34 | 0.15 | 0.14 | 3.06 | 0.45 |
| Bard1 | Bard1 BRCA1 associated RING domain 1 | 0.39 | 0.34 | 0.15 | 0.43 | 0.87 | 0.45 |
| Fat2 | Fat2 FAT atypical cadherin 2 | 0.53 | 0.34 | 0.15 | 0.75 | 0.64 | 0.45 |
| Gabrq | Gabrq gamma-aminobutyric acid (GABA) A receptor, subunit theta | 0.64 | 1.36 | 0.61 | 0.58 | 2.13 | 0.45 |
| Susd3 | Susd3 sushi domain containing 3 | 3.35 | 1.36 | 0.61 | 0.69 | 0.40 | 0.45 |
| Btbd8 | Btbd8 BTB (POZ) domain containing 8 | 1.72 | 1.36 | 0.61 | 0.86 | 0.79 | 0.45 |
| Ankrd34a | Ankrd34a ankyrin repeat domain 34A | 100.49 | 90.83 | 41.02 | 51.76 | 0.90 | 0.45 |
| Tmem116 | Tmem116 transmembrane protein 116 | 2.49 | 2.95 | 1.33 | 1.73 | 1.18 | 0.45 |
| Zswim3 | Zswim3 zinc finger SWIM-type containing 3 | 9.64 | 8.74 | 3.94 | 6.90 | 0.91 | 0.45 |
| Fabp7 | Fabp7 fatty acid binding protein 7, brain | 12.66 | 8.40 | 3.78 | 3.39 | 0.66 | 0.45 |
| Mir7115 | Mir7115 microRNA 7115 | 0.86 | 0.97 | 0.43 | 0.63 | 1.12 | 0.45 |
| Sln | Sln sarcolipin | 1.14 | 0.97 | 0.43 | 0.72 | 0.85 | 0.45 |
| Zfp866 | Zfp866 zinc finger protein 866 | 19.57 | 17.61 | 7.92 | 11.71 | 0.90 | 0.45 |
| Cyp4f18 | Cyp4f18 cytochrome P450, family 4, subfamily f, polypeptide 18 | 0.19 | 1.59 | 0.72 | 0.72 | 8.20 | 0.45 |
| AU021092 | AU021092 expressed sequence AU021092 | 6.85 | 2.50 | 1.12 | 0.26 | 0.37 | 0.45 |
| Avpr1b | Avpr1b arginine vasopressin receptor 1B | 0.64 | 0.63 | 0.28 | 0.46 | 0.98 | 0.45 |
| Glra3 | Glra3 glycine receptor, alpha 3 subunit | 0.53 | 0.91 | 0.41 | 0.32 | 1.73 | 0.45 |
| Mus81 | Mus81 MUS81 structure-specific endonuclease subunit | 40.49 | 39.82 | 17.81 | 20.48 | 0.98 | 0.45 |
| Adgre1 | Adgre1 adhesion G protein-coupled receptor E1 | 4.27 | 9.26 | 4.14 | 3.45 | 2.17 | 0.45 |
| Trmt12 | Trmt12 tRNA methyltranferase 12 | 9.20 | 7.44 | 3.32 | 5.70 | 0.81 | 0.45 |
| Selenov | Selenov selenoprotein V | 0.97 | 1.77 | 0.79 | 0.89 | 1.83 | 0.45 |
| Gm6277 | Gm6277 predicted gene 6277 | 1.64 | 1.77 | 0.79 | 1.32 | 1.09 | 0.45 |
| Proser3 | Proser3 proline and serine rich 3 | 3.02 | 2.64 | 1.18 | 1.61 | 0.87 | 0.45 |
| Map4k1 | Map4k1 mitogen-activated protein kinase kinase kinase kinase 1 | 1.72 | 2.64 | 1.18 | 1.81 | 1.53 | 0.45 |
| 6330418K02Rik | 6330418K02Rik RIKEN cDNA 6330418K02 gene | 0.75 | 0.57 | 0.26 | 0.23 | 0.77 | 0.45 |
| Gpr1 | Gpr1 G protein-coupled receptor 1 | 0.78 | 0.57 | 0.26 | 0.37 | 0.74 | 0.45 |
| Jchain | Jchain immunoglobulin joining chain | 0.25 | 0.57 | 0.26 | 0.40 | 2.30 | 0.45 |
| Il5 | Il5 interleukin 5 | 0.17 | 0.29 | 0.13 | 0.09 | 1.73 | 0.45 |
| Mir377 | Mir377 microRNA 377 | 0.44 | 0.29 | 0.13 | 0.09 | 0.65 | 0.45 |
| Psma8 | Psma8 proteasome (prosome, macropain) subunit, alpha type, 8 | 0.25 | 0.29 | 0.13 | 0.14 | 1.15 | 0.45 |
| Ms4a7 | Ms4a7 membrane-spanning 4-domains, subfamily A, member 7 | 0.30 | 0.29 | 0.13 | 0.14 | 0.94 | 0.45 |
| Gm7030 | Gm7030 predicted gene 7030 | 0.11 | 0.29 | 0.13 | 0.29 | 2.59 | 0.45 |
| Bhlhe23 | Bhlhe23 basic helix-loop-helix family, member e23 | 0.33 | 0.29 | 0.13 | 0.32 | 0.86 | 0.45 |
| Gm10584 | Gm10584 predicted gene 10584 | 1.30 | 1.44 | 0.64 | 0.83 | 1.10 | 0.45 |
| Zfp964 | Zfp964 zinc finger protein 964 | 1.69 | 1.67 | 0.74 | 0.95 | 0.99 | 0.44 |
| Ifi203 | Ifi203 interferon activated gene 203 | 3.30 | 3.05 | 1.35 | 1.75 | 0.93 | 0.44 |
| Vsig8 | Vsig8 V-set and immunoglobulin domain containing 8 | 0.58 | 1.38 | 0.61 | 1.52 | 2.38 | 0.44 |
| Fv1 | Fv1 Friend virus susceptibility 1 | 3.46 | 2.48 | 1.10 | 1.50 | 0.72 | 0.44 |
| Abcc3 | Abcc3 ATP-binding cassette, sub-family C (CFTR/MRP), member 3 | 3.10 | 4.10 | 1.81 | 2.16 | 1.32 | 0.44 |
| Tsacc | Tsacc TSSK6 activating co-chaperone | 1.47 | 2.09 | 0.92 | 1.50 | 1.42 | 0.44 |
| Il21r | Il21r interleukin 21 receptor | 2.16 | 2.56 | 1.12 | 1.81 | 1.18 | 0.44 |
| D430020J02Rik | D430020J02Rik RIKEN cDNA D430020J02 gene | 1.22 | 0.76 | 0.33 | 0.60 | 0.62 | 0.44 |
| A230077H06Rik | A230077H06Rik RIKEN cDNA A230077H06 gene | 5.85 | 4.25 | 1.87 | 1.75 | 0.73 | 0.44 |
| Ngb | Ngb neuroglobin | 8.67 | 18.00 | 7.90 | 11.28 | 2.08 | 0.44 |
| Gm973 | Gm973 predicted gene 973 | 5.68 | 4.49 | 1.97 | 1.81 | 0.79 | 0.44 |
| Slitrk6 | Slitrk6 SLIT and NTRK-like family, member 6 | 0.47 | 1.23 | 0.54 | 0.40 | 2.60 | 0.44 |
| Oma1 | Oma1 OMA1 zinc metallopeptidase | 4.71 | 4.38 | 1.92 | 3.05 | 0.93 | 0.44 |
| Olfml3 | Olfml3 olfactomedin-like 3 | 24.03 | 14.85 | 6.49 | 5.49 | 0.62 | 0.44 |
| Zmym1 | Zmym1 zinc finger, MYM domain containing 1 | 19.95 | 15.86 | 6.93 | 8.08 | 0.80 | 0.44 |
| Crabp1 | Crabp1 cellular retinoic acid binding protein I | 2.00 | 2.64 | 1.15 | 1.15 | 1.32 | 0.44 |
| Nlrp1b | Nlrp1b NLR family, pyrin domain containing 1B | 0.53 | 0.47 | 0.20 | 0.29 | 0.89 | 0.44 |
| Mir124a-2 | Mir124a-2 microRNA 124a-2 | 0.33 | 0.47 | 0.20 | 0.32 | 1.41 | 0.44 |
| Nkapl | Nkapl NFKB activating protein-like | 0.25 | 0.47 | 0.20 | 0.40 | 1.88 | 0.44 |
| Lhcgr | Lhcgr luteinizing hormone/choriogonadotropin receptor | 0.19 | 0.23 | 0.10 | 0.06 | 1.21 | 0.44 |
| Plxna4os1 | Plxna4os1 plexin A4, opposite strand 1 | 0.44 | 0.23 | 0.10 | 0.09 | 0.53 | 0.44 |
| Ppp3r2 | Ppp3r2 protein phosphatase 3, regulatory subunit B, alpha isoform (calcineurin B, type II) | 0.28 | 0.23 | 0.10 | 0.12 | 0.85 | 0.44 |
| Spata4 | Spata4 spermatogenesis associated 4 | 0.28 | 0.23 | 0.10 | 0.12 | 0.85 | 0.44 |
| Sox15 | Sox15 SRY (sex determining region Y)-box 15 | 0.28 | 0.23 | 0.10 | 0.20 | 0.85 | 0.44 |
| Trim43c | Trim43c tripartite motif-containing 43C | 0.30 | 0.23 | 0.10 | 0.20 | 0.77 | 0.44 |
| Caps2 | Caps2 calcyphosphine 2 | 0.53 | 0.23 | 0.10 | 0.20 | 0.45 | 0.44 |
| AF357399 | AF357399 snoRNA AF357399 | 0.25 | 0.23 | 0.10 | 0.23 | 0.94 | 0.44 |
| Myom2 | Myom2 myomesin 2 | 0.19 | 0.23 | 0.10 | 0.32 | 1.21 | 0.44 |
| Cdca5 | Cdca5 cell division cycle associated 5 | 0.25 | 0.23 | 0.10 | 0.37 | 0.94 | 0.44 |
| Cartpt | Cartpt CART prepropeptide | 3.38 | 6.71 | 2.91 | 4.09 | 1.98 | 0.43 |
| Exoc3l4 | Exoc3l4 exocyst complex component 3-like 4 | 3.82 | 4.12 | 1.79 | 2.62 | 1.08 | 0.43 |
| Slc7a7 | Slc7a7 solute carrier family 7 (cationic amino acid transporter, y+ system), member 7 | 2.60 | 4.72 | 2.04 | 2.30 | 1.81 | 0.43 |
| Arhgef19 | Arhgef19 Rho guanine nucleotide exchange factor (GEF) 19 | 32.17 | 34.15 | 14.77 | 11.51 | 1.06 | 0.43 |
| Fam161b | Fam161b family with sequence similarity 161, member B | 12.30 | 10.18 | 4.40 | 7.62 | 0.83 | 0.43 |
| Cckbr | Cckbr cholecystokinin B receptor | 15.05 | 12.97 | 5.60 | 8.63 | 0.86 | 0.43 |
| Sigirr | Sigirr single immunoglobulin and toll-interleukin 1 receptor (TIR) domain | 3.24 | 4.80 | 2.07 | 0.83 | 1.48 | 0.43 |
| Icam2 | Icam2 intercellular adhesion molecule 2 | 5.18 | 9.60 | 4.14 | 4.55 | 1.85 | 0.43 |
| 6030443J06Rik | 6030443J06Rik RIKEN cDNA 6030443J06 gene | 0.55 | 0.65 | 0.28 | 0.26 | 1.18 | 0.43 |
| Gimap4 | Gimap4 GTPase, IMAP family member 4 | 0.17 | 0.65 | 0.28 | 0.35 | 3.92 | 0.43 |
| Rpl3l | Rpl3l ribosomal protein L3-like | 0.55 | 0.65 | 0.28 | 0.66 | 1.18 | 0.43 |
| Zbtb40 | Zbtb40 zinc finger and BTB domain containing 40 | 12.03 | 11.69 | 5.03 | 5.09 | 0.97 | 0.43 |
| Grm8 | Grm8 glutamate receptor, metabotropic 8 | 5.40 | 3.68 | 1.58 | 1.44 | 0.68 | 0.43 |
| Dubr | Dubr Dppa2 upstream binding RNA | 3.57 | 2.56 | 1.10 | 1.58 | 0.72 | 0.43 |
| Hdhd3 | Hdhd3 haloacid dehalogenase-like hydrolase domain containing 3 | 13.52 | 11.09 | 4.75 | 5.38 | 0.82 | 0.43 |
| Esr2 | Esr2 estrogen receptor 2 (beta) | 0.28 | 0.42 | 0.18 | 0.12 | 1.51 | 0.43 |
| Rpl32l | Rpl32l ribosomal protein L32-like | 0.17 | 0.42 | 0.18 | 0.17 | 2.51 | 0.43 |
| Capn3 | Capn3 calpain 3 | 13.69 | 8.30 | 3.55 | 3.14 | 0.61 | 0.43 |
| Fam198a | Fam198a family with sequence similarity 198, member A | 8.95 | 6.81 | 2.91 | 1.99 | 0.76 | 0.43 |
| A330023F24Rik | A330023F24Rik RIKEN cDNA A330023F24 gene | 47.11 | 48.69 | 20.83 | 23.50 | 1.03 | 0.43 |
| BC051226 | BC051226 cDNA sequence BC051226 | 8.51 | 9.58 | 4.09 | 6.82 | 1.13 | 0.43 |
| AI464131 | AI464131 expressed sequence AI464131 | 41.65 | 39.92 | 17.05 | 15.82 | 0.96 | 0.43 |
| Mir6945 | Mir6945 microRNA 6945 | 0.75 | 1.02 | 0.43 | 0.60 | 1.36 | 0.43 |
| Egr2 | Egr2 early growth response 2 | 2.16 | 1.62 | 0.69 | 0.81 | 0.75 | 0.43 |
| Spns3 | Spns3 spinster homolog 3 | 0.47 | 0.60 | 0.26 | 0.26 | 1.27 | 0.43 |
| Sstr5 | Sstr5 somatostatin receptor 5 | 0.39 | 0.60 | 0.26 | 0.29 | 1.55 | 0.43 |
| 1110046J04Rik | 1110046J04Rik RIKEN cDNA 1110046J04 gene | 1.50 | 1.98 | 0.84 | 0.92 | 1.33 | 0.43 |
| Lingo4 | Lingo4 leucine rich repeat and Ig domain containing 4 | 0.78 | 0.78 | 0.33 | 0.40 | 1.01 | 0.42 |
| E130006D01Rik | E130006D01Rik RIKEN cDNA E130006D01 gene | 0.64 | 0.97 | 0.41 | 0.58 | 1.51 | 0.42 |
| Zfp953 | Zfp953 zinc finger protein 953 | 1.39 | 0.97 | 0.41 | 0.60 | 0.70 | 0.42 |
| Tnfrsf25 | Tnfrsf25 tumor necrosis factor receptor superfamily, member 25 | 17.07 | 12.32 | 5.21 | 4.32 | 0.72 | 0.42 |
| Gal3st4 | Gal3st4 galactose-3-O-sulfotransferase 4 | 3.66 | 3.26 | 1.38 | 1.50 | 0.89 | 0.42 |
| Rhcg | Rhcg Rhesus blood group-associated C glycoprotein | 0.72 | 1.15 | 0.49 | 0.23 | 1.59 | 0.42 |
| Il27ra | Il27ra interleukin 27 receptor, alpha | 2.47 | 2.30 | 0.97 | 1.01 | 0.93 | 0.42 |
| Tmem107 | Tmem107 transmembrane protein 107 | 12.19 | 9.55 | 4.04 | 4.52 | 0.78 | 0.42 |
| Prss57 | Prss57 protease, serine 57 | 1.64 | 1.51 | 0.64 | 0.92 | 0.93 | 0.42 |
| Acvrl1 | Acvrl1 activin A receptor, type II-like 1 | 19.68 | 30.14 | 12.70 | 14.56 | 1.53 | 0.42 |
| Zbtb3 | Zbtb3 zinc finger and BTB domain containing 3 | 5.79 | 5.11 | 2.15 | 4.43 | 0.88 | 0.42 |
| Prickle4 | Prickle4 prickle planar cell polarity protein 4 | 0.28 | 0.37 | 0.15 | 0.20 | 1.32 | 0.42 |
| Il17b | Il17b interleukin 17B | 0.42 | 0.37 | 0.15 | 0.20 | 0.88 | 0.42 |
| B130024G19Rik | B130024G19Rik RIKEN cDNA B130024G19 gene | 0.19 | 0.37 | 0.15 | 0.35 | 1.88 | 0.42 |
| Mir7667 | Mir7667 microRNA 7667 | 0.17 | 0.18 | 0.08 | 0.03 | 1.10 | 0.42 |
| Gm609 | Gm609 predicted gene 609 | 0.25 | 0.18 | 0.08 | 0.03 | 0.73 | 0.42 |
| 1600029I14Rik | 1600029I14Rik RIKEN cDNA 1600029I14 gene | 0.17 | 0.18 | 0.08 | 0.06 | 1.10 | 0.42 |
| 5033404E19Rik | 5033404E19Rik RIKEN cDNA 5033404E19 gene | 0.19 | 0.18 | 0.08 | 0.09 | 0.94 | 0.42 |
| Myl2 | Myl2 myosin, light polypeptide 2, regulatory, cardiac, slow | 0.30 | 0.18 | 0.08 | 0.09 | 0.60 | 0.42 |
| Serpina9 | Serpina9 serine (or cysteine) peptidase inhibitor, clade A (alpha-1 antiproteinase, antitrypsin), member 9 | 0.03 | 0.18 | 0.08 | 0.12 | 6.59 | 0.42 |
| Slc13a1 | Slc13a1 solute carrier family 13 (sodium/sulfate symporters), member 1 | 0.06 | 0.18 | 0.08 | 0.12 | 3.30 | 0.42 |
| Gm6498 | Gm6498 predicted gene 6498 | 0.11 | 0.18 | 0.08 | 0.12 | 1.65 | 0.42 |
| 1810062G17Rik | 1810062G17Rik RIKEN cDNA 1810062G17 gene | 0.17 | 0.18 | 0.08 | 0.12 | 1.10 | 0.42 |
| Gm17751 | Gm17751 predicted gene, 17751 | 0.08 | 0.18 | 0.08 | 0.14 | 2.20 | 0.42 |
| Rsph14 | Rsph14 radial spoke head homolog 14 (Chlamydomonas) | 0.28 | 0.18 | 0.08 | 0.14 | 0.66 | 0.42 |
| AA543186 | AA543186 expressed sequence AA543186 | 0.36 | 0.18 | 0.08 | 0.14 | 0.51 | 0.42 |
| C1qtnf3 | C1qtnf3 C1q and tumor necrosis factor related protein 3 | 0.19 | 0.18 | 0.08 | 0.20 | 0.94 | 0.42 |
| Kif18b | Kif18b kinesin family member 18B | 0.25 | 0.18 | 0.08 | 0.23 | 0.73 | 0.42 |
| Slc16a5 | Slc16a5 solute carrier family 16 (monocarboxylic acid transporters), member 5 | 1.25 | 1.10 | 0.46 | 0.78 | 0.88 | 0.42 |
| Wdcp | Wdcp WD repeat and coiled coil containing | 7.70 | 6.16 | 2.58 | 4.78 | 0.80 | 0.42 |
| Cyb5rl | Cyb5rl cytochrome b5 reductase-like | 5.71 | 5.24 | 2.20 | 3.51 | 0.92 | 0.42 |
| AW146154 | AW146154 expressed sequence AW146154 | 3.41 | 2.69 | 1.12 | 1.75 | 0.79 | 0.42 |
| Pkn3 | Pkn3 protein kinase N3 | 2.58 | 5.32 | 2.22 | 1.90 | 2.07 | 0.42 |
| Trim14 | Trim14 tripartite motif-containing 14 | 1.41 | 1.59 | 0.66 | 0.86 | 1.13 | 0.42 |
| BC030499 | BC030499 cDNA sequence BC030499 | 4.71 | 4.04 | 1.69 | 1.47 | 0.86 | 0.42 |
| Trpc7 | Trpc7 transient receptor potential cation channel, subfamily C, member 7 | 2.38 | 3.13 | 1.30 | 1.58 | 1.31 | 0.42 |
| 4930579K19Rik | 4930579K19Rik RIKEN cDNA 4930579K19 gene | 0.72 | 1.04 | 0.43 | 0.86 | 1.45 | 0.42 |
| Fzd10 | Fzd10 frizzled class receptor 10 | 1.05 | 0.86 | 0.36 | 0.32 | 0.82 | 0.42 |
| Rtp1 | Rtp1 receptor transporter protein 1 | 0.36 | 2.22 | 0.92 | 1.18 | 6.16 | 0.41 |
| Npas4 | Npas4 neuronal PAS domain protein 4 | 17.46 | 17.22 | 7.13 | 7.42 | 0.99 | 0.41 |
| Snx20 | Snx20 sorting nexin 20 | 1.52 | 3.52 | 1.46 | 2.76 | 2.31 | 0.41 |
| Gja5 | Gja5 gap junction protein, alpha 5 | 0.72 | 1.17 | 0.49 | 0.63 | 1.63 | 0.41 |
| Fndc8 | Fndc8 fibronectin type III domain containing 8 | 3.33 | 2.35 | 0.97 | 1.35 | 0.71 | 0.41 |
| Ifit1bl2 | Ifit1bl2 interferon induced protein with tetratricopeptide repeats 1B like 2 | 0.36 | 0.50 | 0.20 | 0.29 | 1.38 | 0.41 |
| Spaar | Spaar small regulatory polypeptide of amino acid response | 0.75 | 0.99 | 0.41 | 0.46 | 1.33 | 0.41 |
| Ccdc190 | Ccdc190 coiled-coil domain containing 190 | 11.81 | 9.73 | 4.01 | 4.11 | 0.82 | 0.41 |
| Gm13547 | Gm13547 predicted gene 13547 | 0.30 | 0.31 | 0.13 | 0.37 | 1.03 | 0.41 |
| Nme6 | Nme6 NME/NM23 nucleoside diphosphate kinase 6 | 8.67 | 7.59 | 3.09 | 4.80 | 0.88 | 0.41 |
| Sox17 | Sox17 SRY (sex determining region Y)-box 17 | 6.76 | 6.34 | 2.58 | 2.50 | 0.94 | 0.41 |
| C730002L08Rik | C730002L08Rik RIKEN cDNA C730002L08 gene | 1.27 | 1.38 | 0.56 | 1.27 | 1.08 | 0.41 |
| Gsto2 | Gsto2 glutathione S-transferase omega 2 | 0.97 | 1.07 | 0.43 | 0.58 | 1.10 | 0.41 |
| Slamf8 | Slamf8 SLAM family member 8 | 0.22 | 1.07 | 0.43 | 0.63 | 4.83 | 0.41 |
| Slamf9 | Slamf9 SLAM family member 9 | 1.47 | 4.28 | 1.74 | 4.17 | 2.91 | 0.41 |
| Crb1 | Crb1 crumbs family member 1, photoreceptor morphogenesis associated | 0.50 | 0.76 | 0.31 | 0.58 | 1.52 | 0.41 |
| Gm10560 | Gm10560 predicted gene 10560 | 3.69 | 4.23 | 1.71 | 2.65 | 1.15 | 0.41 |
| Gm10941 | Gm10941 predicted gene 10941 | 1.03 | 1.20 | 0.49 | 0.49 | 1.17 | 0.40 |
| Tnfrsf18 | Tnfrsf18 tumor necrosis factor receptor superfamily, member 18 | 3.96 | 4.17 | 1.69 | 3.45 | 1.05 | 0.40 |
| Gm11423 | Gm11423 predicted gene 11423 | 0.80 | 0.89 | 0.36 | 0.40 | 1.10 | 0.40 |
| Zfp934 | Zfp934 zinc finger protein 934 | 1.00 | 0.89 | 0.36 | 0.40 | 0.89 | 0.40 |
| Tifab | Tifab TRAF-interacting protein with forkhead-associated domain, family member B | 2.94 | 3.81 | 1.53 | 2.88 | 1.30 | 0.40 |
| Nat8f1 | Nat8f1 N-acetyltransferase 8 (GCN5-related) family member 1 | 13.08 | 15.97 | 6.41 | 7.88 | 1.22 | 0.40 |
| Ushbp1 | Ushbp1 USH1 protein network component harmonin binding protein 1 | 7.76 | 4.67 | 1.87 | 1.61 | 0.60 | 0.40 |
| Noxred1 | Noxred1 NADP+ dependent oxidoreductase domain containing 1 | 1.11 | 0.70 | 0.28 | 0.49 | 0.64 | 0.40 |
| A630089N07Rik | A630089N07Rik RIKEN cDNA A630089N07 gene | 0.55 | 0.70 | 0.28 | 0.55 | 1.27 | 0.40 |
| Ccdc191 | Ccdc191 coiled-coil domain containing 191 | 4.54 | 3.42 | 1.35 | 2.22 | 0.75 | 0.40 |
| Cx3cr1 | Cx3cr1 chemokine (C-X3-C motif) receptor 1 | 44.73 | 48.45 | 19.19 | 25.46 | 1.08 | 0.40 |
| Zmat1 | Zmat1 zinc finger, matrin type 1 | 8.34 | 9.63 | 3.81 | 5.96 | 1.15 | 0.40 |
| C130083M11Rik | C130083M11Rik RIKEN cDNA C130083M11 gene | 2.08 | 1.49 | 0.59 | 1.09 | 0.72 | 0.40 |
| En1 | En1 engrailed 1 | 0.06 | 0.13 | 0.05 | 0.03 | 2.35 | 0.39 |
| Mirlet7i | Mirlet7i microRNA let7i | 0.06 | 0.13 | 0.05 | 0.03 | 2.35 | 0.39 |
| Abhd12b | Abhd12b abhydrolase domain containing 12B | 0.06 | 0.13 | 0.05 | 0.03 | 2.35 | 0.39 |
| C330022C24Rik | C330022C24Rik RIKEN cDNA C330022C24 gene | 0.08 | 0.13 | 0.05 | 0.03 | 1.57 | 0.39 |
| Fbxo15 | Fbxo15 F-box protein 15 | 0.14 | 0.13 | 0.05 | 0.03 | 0.94 | 0.39 |
| S100a14 | S100a14 S100 calcium binding protein A14 | 0.03 | 0.13 | 0.05 | 0.06 | 4.71 | 0.39 |
| Il1bos | Il1bos interleukin 1 beta, opposite strand | 0.06 | 0.13 | 0.05 | 0.06 | 2.35 | 0.39 |
| Gm17359 | Gm17359 predicted gene, 17359 | 0.06 | 0.13 | 0.05 | 0.06 | 2.35 | 0.39 |
| Gm9054 | Gm9054 predicted gene 9054 | 0.08 | 0.13 | 0.05 | 0.06 | 1.57 | 0.39 |
| Zfp750 | Zfp750 zinc finger protein 750 | 0.08 | 0.13 | 0.05 | 0.06 | 1.57 | 0.39 |
| Gm16294 | Gm16294 predicted gene 16294 | 0.14 | 0.13 | 0.05 | 0.06 | 0.94 | 0.39 |
| Pcdhb1 | Pcdhb1 protocadherin beta 1 | 0.19 | 0.13 | 0.05 | 0.06 | 0.67 | 0.39 |
| Mhrt | Mhrt myosin heavy chain associated RNA transcript | 0.03 | 0.13 | 0.05 | 0.09 | 4.71 | 0.39 |
| Otc | Otc ornithine transcarbamylase | 0.08 | 0.13 | 0.05 | 0.09 | 1.57 | 0.39 |
| Mir382 | Mir382 microRNA 382 | 0.14 | 0.13 | 0.05 | 0.09 | 0.94 | 0.39 |
| Mir466f-2 | Mir466f-2 microRNA 466f-2 | 0.06 | 0.13 | 0.05 | 0.12 | 2.35 | 0.39 |
| Fmr1os | Fmr1os fragile X mental retardation 1, opposite strand | 0.17 | 0.13 | 0.05 | 0.12 | 0.78 | 0.39 |
| Cd200r1 | Cd200r1 CD200 receptor 1 | 0.25 | 0.13 | 0.05 | 0.12 | 0.52 | 0.39 |
| Spem2 | Spem2 SPEM family member 2 | 0.03 | 0.13 | 0.05 | 0.14 | 4.71 | 0.39 |
| Cdkn2a | Cdkn2a cyclin dependent kinase inhibitor 2A | 0.06 | 0.13 | 0.05 | 0.14 | 2.35 | 0.39 |
| 3100003L05Rik | 3100003L05Rik RIKEN cDNA 3100003L05 gene | 0.08 | 0.13 | 0.05 | 0.17 | 1.57 | 0.39 |
| Mmp21 | Mmp21 matrix metallopeptidase 21 | 0.08 | 0.13 | 0.05 | 0.17 | 1.57 | 0.39 |
| Mettl7a3 | Mettl7a3 methyltransferase like 7A3 | 0.06 | 0.13 | 0.05 | 0.23 | 2.35 | 0.39 |
| Kiss1 | Kiss1 KiSS-1 metastasis-suppressor | 0.06 | 0.13 | 0.05 | 0.29 | 2.35 | 0.39 |
| Tacr2 | Tacr2 tachykinin receptor 2 | 0.25 | 0.13 | 0.05 | 0.29 | 0.52 | 0.39 |
| AA387883 | AA387883 expressed sequence AA387883 | 1.50 | 1.30 | 0.51 | 0.35 | 0.87 | 0.39 |
| **F12** | **F12 coagulation factor XII (Hageman factor)** | **0.75** | **1.17** | **0.46** | **0.89** | **1.57** | **0.39** |
| Magix | Magix MAGI family member, X-linked | 0.69 | 0.39 | 0.15 | 0.17 | 0.56 | 0.39 |
| Fbxo47 | Fbxo47 F-box protein 47 | 0.61 | 0.39 | 0.15 | 0.26 | 0.64 | 0.39 |
| Masp2 | Masp2 mannan-binding lectin serine peptidase 2 | 1.05 | 0.65 | 0.26 | 0.32 | 0.62 | 0.39 |
| A530050N04Rik | A530050N04Rik RIKEN cDNA A530050N04 gene | 0.17 | 0.26 | 0.10 | 0.06 | 1.57 | 0.39 |
| Trem3 | Trem3 triggering receptor expressed on myeloid cells 3 | 0.11 | 0.26 | 0.10 | 0.09 | 2.35 | 0.39 |
| Gm13710 | Gm13710 predicted gene 13710 | 0.11 | 0.26 | 0.10 | 0.12 | 2.35 | 0.39 |
| Fsd2 | Fsd2 fibronectin type III and SPRY domain containing 2 | 0.30 | 0.26 | 0.10 | 0.14 | 0.86 | 0.39 |
| Dupd1 | Dupd1 dual specificity phosphatase and pro isomerase domain containing 1 | 0.30 | 0.26 | 0.10 | 0.14 | 0.86 | 0.39 |
| Zbbx | Zbbx zinc finger, B-box domain containing | 0.55 | 0.26 | 0.10 | 0.14 | 0.47 | 0.39 |
| Cyp1a1 | Cyp1a1 cytochrome P450, family 1, subfamily a, polypeptide 1 | 0.22 | 0.26 | 0.10 | 0.17 | 1.18 | 0.39 |
| 4931429L15Rik | 4931429L15Rik RIKEN cDNA 4931429L15 gene | 0.25 | 0.26 | 0.10 | 0.17 | 1.05 | 0.39 |
| Hsd17b1 | Hsd17b1 hydroxysteroid (17-beta) dehydrogenase 1 | 0.28 | 0.26 | 0.10 | 0.17 | 0.94 | 0.39 |
| Gdf7 | Gdf7 growth differentiation factor 7 | 0.30 | 0.26 | 0.10 | 0.20 | 0.86 | 0.39 |
| Efcab9 | Efcab9 EF-hand calcium binding domain 9 | 0.17 | 0.26 | 0.10 | 0.23 | 1.57 | 0.39 |
| Slc12a3 | Slc12a3 solute carrier family 12, member 3 | 0.06 | 0.26 | 0.10 | 0.29 | 4.71 | 0.39 |
| Xpnpep2 | Xpnpep2 X-prolyl aminopeptidase (aminopeptidase P) 2, membrane-bound | 0.58 | 0.52 | 0.20 | 0.32 | 0.90 | 0.39 |
| Mboat4 | Mboat4 membrane bound O-acyltransferase domain containing 4 | 0.28 | 0.52 | 0.20 | 0.40 | 1.88 | 0.39 |
| Frem3 | Frem3 Fras1 related extracellular matrix protein 3 | 0.78 | 1.44 | 0.56 | 1.52 | 1.85 | 0.39 |
| Zfp759 | Zfp759 zinc finger protein 759 | 4.05 | 4.25 | 1.66 | 2.50 | 1.05 | 0.39 |
| Pilra | Pilra paired immunoglobin-like type 2 receptor alpha | 0.17 | 4.12 | 1.61 | 2.24 | 24.79 | 0.39 |
| Pctp | Pctp phosphatidylcholine transfer protein | 1.41 | 1.64 | 0.64 | 0.78 | 1.16 | 0.39 |
| Csf3r | Csf3r colony stimulating factor 3 receptor (granulocyte) | 7.34 | 7.96 | 3.09 | 3.77 | 1.08 | 0.39 |
| Irs4 | Irs4 insulin receptor substrate 4 | 0.36 | 1.51 | 0.59 | 0.43 | 4.20 | 0.39 |
| E530001F21Rik | E530001F21Rik RIKEN cDNA E530001F21 gene | 0.75 | 1.12 | 0.43 | 0.46 | 1.50 | 0.39 |
| Atg16l2 | Atg16l2 autophagy related 16-like 2 (S. cerevisiae) | 19.73 | 24.97 | 9.66 | 13.92 | 1.27 | 0.39 |
| 6330415G19Rik | 6330415G19Rik RIKEN cDNA 6330415G19 gene | 0.83 | 1.72 | 0.66 | 1.55 | 2.07 | 0.39 |
| Bhlha9 | Bhlha9 basic helix-loop-helix family, member a9 | 4.02 | 5.04 | 1.94 | 4.89 | 1.25 | 0.39 |
| Zfp456 | Zfp456 zinc finger protein 456 | 0.67 | 0.73 | 0.28 | 0.35 | 1.10 | 0.38 |
| Baiap3 | Baiap3 BAI1-associated protein 3 | 17.65 | 38.49 | 14.80 | 15.39 | 2.18 | 0.38 |
| Gjd3 | Gjd3 gap junction protein, delta 3 | 1.03 | 2.06 | 0.79 | 1.44 | 2.01 | 0.38 |
| Lekr1 | Lekr1 leucine, glutamate and lysine rich 1 | 2.94 | 2.53 | 0.97 | 0.92 | 0.86 | 0.38 |
| Atp6v0c | Atp6v0c ATPase, H+ transporting, lysosomal V0 subunit C | 0.30 | 0.60 | 0.23 | 0.23 | 1.97 | 0.38 |
| Mirt1 | Mirt1 myocardial infarction associated transcript 1 | 5.35 | 6.08 | 2.33 | 4.06 | 1.14 | 0.38 |
| Hrh1 | Hrh1 histamine receptor H1 | 8.37 | 6.63 | 2.53 | 3.83 | 0.79 | 0.38 |
| Nat1 | Nat1 N-acetyl transferase 1 | 1.36 | 0.94 | 0.36 | 0.23 | 0.69 | 0.38 |
| Bbs12 | Bbs12 Bardet-Biedl syndrome 12 (human) | 3.63 | 4.23 | 1.61 | 1.73 | 1.16 | 0.38 |
| Klhl35 | Klhl35 kelch-like 35 | 1.69 | 3.16 | 1.20 | 2.79 | 1.87 | 0.38 |
| Lck | Lck lymphocyte protein tyrosine kinase | 2.49 | 3.37 | 1.28 | 1.96 | 1.35 | 0.38 |
| Exd1 | Exd1 exonuclease 3'-5' domain containing 1 | 1.11 | 0.81 | 0.31 | 0.55 | 0.73 | 0.38 |
| Htr1b | Htr1b 5-hydroxytryptamine (serotonin) receptor 1B | 1.22 | 1.36 | 0.51 | 0.46 | 1.11 | 0.38 |
| Fezf1 | Fezf1 Fez family zinc finger 1 | 0.06 | 0.34 | 0.13 | 0.03 | 6.12 | 0.38 |
| Cyp2f2 | Cyp2f2 cytochrome P450, family 2, subfamily f, polypeptide 2 | 0.30 | 0.34 | 0.13 | 0.03 | 1.11 | 0.38 |
| Gm10863 | Gm10863 predicted gene 10863 | 0.61 | 0.34 | 0.13 | 0.03 | 0.56 | 0.38 |
| Nat8f3 | Nat8f3 N-acetyltransferase 8 (GCN5-related) family member 3 | 0.61 | 0.34 | 0.13 | 0.12 | 0.56 | 0.38 |
| Fam83g | Fam83g family with sequence similarity 83, member G | 0.36 | 0.34 | 0.13 | 0.14 | 0.94 | 0.38 |
| Gm9895 | Gm9895 predicted gene 9895 | 0.19 | 0.34 | 0.13 | 0.26 | 1.75 | 0.38 |
| Slc10a1 | Slc10a1 solute carrier family 10 (sodium/bile acid cotransporter family), member 1 | 0.14 | 0.34 | 0.13 | 0.29 | 2.45 | 0.38 |
| Guca1a | Guca1a guanylate cyclase activator 1a (retina) | 0.36 | 0.34 | 0.13 | 0.46 | 0.94 | 0.38 |
| AI467606 | AI467606 expressed sequence AI467606 | 2.97 | 4.96 | 1.87 | 1.90 | 1.67 | 0.38 |
| Lct | Lct lactase | 52.96 | 33.97 | 12.75 | 23.22 | 0.64 | 0.38 |
| Apoc3 | Apoc3 apolipoprotein C-III | 0.33 | 1.23 | 0.46 | 0.52 | 3.69 | 0.38 |
| C630031E19Rik | C630031E19Rik RIKEN cDNA C630031E19 gene | 2.00 | 1.77 | 0.66 | 0.72 | 0.89 | 0.37 |
| Cd52 | Cd52 CD52 antigen | 1.58 | 7.83 | 2.91 | 3.37 | 4.96 | 0.37 |
| Gpr179 | Gpr179 G protein-coupled receptor 179 | 0.50 | 2.27 | 0.84 | 1.18 | 4.55 | 0.37 |
| Rhoh | Rhoh ras homolog family member H | 0.64 | 0.76 | 0.28 | 0.37 | 1.19 | 0.37 |
| Gm9958 | Gm9958 predicted gene 9958 | 1.11 | 0.97 | 0.36 | 0.89 | 0.87 | 0.37 |
| Depdc7 | Depdc7 DEP domain containing 7 | 1.64 | 1.17 | 0.43 | 0.55 | 0.72 | 0.37 |
| Map2k3os | Map2k3os mitogen-activated protein kinase kinase 3, opposite strand | 0.97 | 1.17 | 0.43 | 0.66 | 1.21 | 0.37 |
| Nadsyn1 | Nadsyn1 NAD synthetase 1 | 4.68 | 5.32 | 1.97 | 2.19 | 1.14 | 0.37 |
| Zfp524 | Zfp524 zinc finger protein 524 | 11.39 | 10.93 | 4.04 | 6.56 | 0.96 | 0.37 |
| Sftpc | Sftpc surfactant associated protein C | 1.69 | 1.80 | 0.66 | 0.92 | 1.06 | 0.37 |
| Fam111a | Fam111a family with sequence similarity 111, member A | 1.30 | 2.64 | 0.97 | 1.58 | 2.02 | 0.37 |
| Vmn2r87 | Vmn2r87 vomeronasal 2, receptor 87 | 0.50 | 0.42 | 0.15 | 0.20 | 0.84 | 0.37 |
| Pde6h | Pde6h phosphodiesterase 6H, cGMP-specific, cone, gamma | 0.14 | 0.42 | 0.15 | 0.29 | 3.01 | 0.37 |
| 5430427O19Rik | 5430427O19Rik RIKEN cDNA 5430427O19 gene | 0.19 | 0.42 | 0.15 | 0.29 | 2.15 | 0.37 |
| 2310040G24Rik | 2310040G24Rik RIKEN cDNA 2310040G24 gene | 0.58 | 0.42 | 0.15 | 0.43 | 0.72 | 0.37 |
| 1700019A02Rik | 1700019A02Rik RIKEN cDNA 1700019A02 gene | 0.08 | 0.21 | 0.08 | 0.03 | 2.51 | 0.37 |
| Dlx4os | Dlx4os distal-less homeobox 4, opposite strand | 0.19 | 0.21 | 0.08 | 0.03 | 1.08 | 0.37 |
| Fmr1nb | Fmr1nb Fmr1 neighbor | 0.03 | 0.21 | 0.08 | 0.09 | 7.53 | 0.37 |
| Pebp4 | Pebp4 phosphatidylethanolamine binding protein 4 | 0.11 | 0.21 | 0.08 | 0.09 | 1.88 | 0.37 |
| Klhdc7b | Klhdc7b kelch domain containing 7B | 0.17 | 0.21 | 0.08 | 0.09 | 1.26 | 0.37 |
| Cct8l1 | Cct8l1 chaperonin containing TCP1, subunit 8 (theta)-like 1 | 0.22 | 0.21 | 0.08 | 0.12 | 0.94 | 0.37 |
| 4930452G13Rik | 4930452G13Rik RIKEN cDNA 4930452G13 gene | 0.33 | 0.21 | 0.08 | 0.14 | 0.63 | 0.37 |
| Nlrp1c-ps | Nlrp1c-ps NLR family, pyrin domain containing 1C, pseudogene | 0.44 | 0.21 | 0.08 | 0.14 | 0.47 | 0.37 |
| Vgll2 | Vgll2 vestigial like family member 2 | 0.06 | 0.21 | 0.08 | 0.17 | 3.77 | 0.37 |
| 1600014C23Rik | 1600014C23Rik RIKEN cDNA 1600014C23 gene | 0.11 | 0.21 | 0.08 | 0.17 | 1.88 | 0.37 |
| Gnat2 | Gnat2 guanine nucleotide binding protein, alpha transducing 2 | 0.17 | 0.21 | 0.08 | 0.20 | 1.26 | 0.37 |
| D930028M14Rik | D930028M14Rik RIKEN cDNA D930028M14 gene | 0.28 | 0.21 | 0.08 | 0.23 | 0.75 | 0.37 |
| Tulp2 | Tulp2 tubby-like protein 2 | 0.25 | 0.21 | 0.08 | 0.26 | 0.84 | 0.37 |
| Pla2g4d | Pla2g4d phospholipase A2, group IVD | 0.11 | 0.21 | 0.08 | 0.37 | 1.88 | 0.37 |
| Gm5141 | Gm5141 predicted gene 5141 | 1.86 | 2.04 | 0.74 | 1.35 | 1.10 | 0.36 |
| A330040F15Rik | A330040F15Rik RIKEN cDNA A330040F15 gene | 1.80 | 2.53 | 0.92 | 0.95 | 1.41 | 0.36 |
| Uts2 | Uts2 urotensin 2 | 0.11 | 0.29 | 0.10 | 0.06 | 2.59 | 0.36 |
| Nlrp1a | Nlrp1a NLR family, pyrin domain containing 1A | 0.11 | 0.29 | 0.10 | 0.12 | 2.59 | 0.36 |
| A830009L08Rik | A830009L08Rik RIKEN cDNA A830009L08 gene | 0.42 | 0.29 | 0.10 | 0.17 | 0.69 | 0.36 |
| Prss56 | Prss56 protease, serine 56 | 0.11 | 0.29 | 0.10 | 0.29 | 2.59 | 0.36 |
| Gpr84 | Gpr84 G protein-coupled receptor 84 | 1.61 | 32.95 | 11.65 | 22.21 | 20.50 | 0.35 |
| **Casr** | **Casr calcium-sensing receptor** | **0.14** | **0.65** | **0.23** | **0.14** | **4.71** | **0.35** |
| 3110045C21Rik | 3110045C21Rik RIKEN cDNA 3110045C21 gene | 0.72 | 0.65 | 0.23 | 0.29 | 0.91 | 0.35 |
| Tek | Tek TEK receptor tyrosine kinase | 13.30 | 9.84 | 3.45 | 3.31 | 0.74 | 0.35 |
| Mir409 | Mir409 microRNA 409 | 0.36 | 0.37 | 0.13 | 0.09 | 1.01 | 0.35 |
| 2210416O15Rik | 2210416O15Rik RIKEN cDNA 2210416O15 gene | 0.86 | 2.06 | 0.72 | 1.27 | 2.40 | 0.35 |
| Serpinb6b | Serpinb6b serine (or cysteine) peptidase inhibitor, clade B, member 6b | 1.25 | 0.89 | 0.31 | 0.14 | 0.71 | 0.35 |
| Btc | Btc betacellulin, epidermal growth factor family member | 0.50 | 0.44 | 0.15 | 0.26 | 0.89 | 0.35 |
| Gpr17 | Gpr17 G protein-coupled receptor 17 | 41.90 | 29.82 | 10.22 | 7.59 | 0.71 | 0.34 |
| Mei4 | Mei4 meiotic double-stranded break formation protein 4 | 0.80 | 0.60 | 0.20 | 0.26 | 0.75 | 0.34 |
| Hcrtr2 | Hcrtr2 hypocretin (orexin) receptor 2 | 1.97 | 2.27 | 0.77 | 0.86 | 1.15 | 0.34 |
| Gmnc | Gmnc geminin coiled-coil domain containing | 3.85 | 3.65 | 1.23 | 1.41 | 0.95 | 0.34 |
| Ecel1 | Ecel1 endothelin converting enzyme-like 1 | 7.48 | 15.50 | 5.19 | 11.80 | 2.07 | 0.33 |
| Abcb1a | Abcb1a ATP-binding cassette, sub-family B (MDR/TAP), member 1A | 25.50 | 21.63 | 7.23 | 6.36 | 0.85 | 0.33 |
| **Ccl12** | **Ccl12 chemokine (C-C motif) ligand 12** | **0.53** | **8.85** | **2.94** | **2.88** | **16.80** | **0.33** |
| Myo1f | Myo1f myosin IF | 3.46 | 4.57 | 1.51 | 1.70 | 1.32 | 0.33 |
| Krt85 | Krt85 keratin 85 | 0.22 | 0.31 | 0.10 | 0.09 | 1.41 | 0.33 |
| C030016D13Rik | C030016D13Rik RIKEN cDNA C030016D13 gene | 0.61 | 0.31 | 0.10 | 0.12 | 0.51 | 0.33 |
| 1600010M07Rik | 1600010M07Rik RIKEN cDNA 1600010M07 gene | 0.08 | 0.31 | 0.10 | 0.17 | 3.77 | 0.33 |
| Fsbp | Fsbp fibrinogen silencer binding protein | 0.08 | 0.31 | 0.10 | 0.20 | 3.77 | 0.33 |
| Gabre | Gabre gamma-aminobutyric acid (GABA) A receptor, subunit epsilon | 0.11 | 0.31 | 0.10 | 0.20 | 2.82 | 0.33 |
| BC051537 | BC051537 cDNA sequence BC051537 | 0.17 | 0.31 | 0.10 | 0.23 | 1.88 | 0.33 |
| Olfr288 | Olfr288 olfactory receptor 288 | 0.19 | 0.31 | 0.10 | 0.26 | 1.61 | 0.33 |
| Zfp663 | Zfp663 zinc finger protein 663 | 0.06 | 0.08 | 0.03 | 0.03 | 1.41 | 0.33 |
| 1700034I23Rik | 1700034I23Rik RIKEN cDNA 1700034I23 gene | 0.06 | 0.08 | 0.03 | 0.03 | 1.41 | 0.33 |
| Vsx1 | Vsx1 visual system homeobox 1 | 0.08 | 0.08 | 0.03 | 0.03 | 0.94 | 0.33 |
| Mir3057 | Mir3057 microRNA 3057 | 0.08 | 0.08 | 0.03 | 0.03 | 0.94 | 0.33 |
| Fam25c | Fam25c family with sequence similarity 25, member C | 0.08 | 0.08 | 0.03 | 0.03 | 0.94 | 0.33 |
| Mfsd4b3 | Mfsd4b3 major facilitator superfamily domain containing 4B3 | 0.11 | 0.08 | 0.03 | 0.03 | 0.71 | 0.33 |
| Gm1965 | Gm1965 predicted gene 1965 | 0.03 | 0.08 | 0.03 | 0.06 | 2.82 | 0.33 |
| Rp1l1 | Rp1l1 retinitis pigmentosa 1 homolog like 1 | 0.03 | 0.08 | 0.03 | 0.06 | 2.82 | 0.33 |
| Olfr46 | Olfr46 olfactory receptor 46 | 0.06 | 0.08 | 0.03 | 0.06 | 1.41 | 0.33 |
| Gm10440 | Gm10440 predicted gene 10440 | 0.08 | 0.08 | 0.03 | 0.06 | 0.94 | 0.33 |
| Gm5111 | Gm5111 predicted gene 5111 | 0.08 | 0.08 | 0.03 | 0.06 | 0.94 | 0.33 |
| Mir320 | Mir320 microRNA 320 | 0.08 | 0.08 | 0.03 | 0.06 | 0.94 | 0.33 |
| Mir142b | Mir142b microRNA 142b | 0.11 | 0.08 | 0.03 | 0.06 | 0.71 | 0.33 |
| Trpm1 | Trpm1 transient receptor potential cation channel, subfamily M, member 1 | 0.14 | 0.08 | 0.03 | 0.06 | 0.56 | 0.33 |
| 4930405J17Rik | 4930405J17Rik RIKEN cDNA 4930405J17 gene | 0.03 | 0.08 | 0.03 | 0.09 | 2.82 | 0.33 |
| Gja8 | Gja8 gap junction protein, alpha 8 | 0.06 | 0.08 | 0.03 | 0.09 | 1.41 | 0.33 |
| Hist1h4a | Hist1h4a histone cluster 1, H4a | 0.06 | 0.08 | 0.03 | 0.09 | 1.41 | 0.33 |
| Gm6194 | Gm6194 predicted gene 6194 | 0.08 | 0.08 | 0.03 | 0.09 | 0.94 | 0.33 |
| Ace3 | Ace3 angiotensin I converting enzyme (peptidyl-dipeptidase A) 3 | 0.08 | 0.08 | 0.03 | 0.09 | 0.94 | 0.33 |
| Mir7662 | Mir7662 microRNA 7662 | 0.14 | 0.08 | 0.03 | 0.09 | 0.56 | 0.33 |
| Abca13 | Abca13 ATP-binding cassette, sub-family A (ABC1), member 13 | 0.19 | 0.08 | 0.03 | 0.09 | 0.40 | 0.33 |
| Cdcp1 | Cdcp1 CUB domain containing protein 1 | 0.03 | 0.08 | 0.03 | 0.12 | 2.82 | 0.33 |
| Syngr4 | Syngr4 synaptogyrin 4 | 0.08 | 0.08 | 0.03 | 0.12 | 0.94 | 0.33 |
| Krt75 | Krt75 keratin 75 | 0.08 | 0.08 | 0.03 | 0.12 | 0.94 | 0.33 |
| 4930413F20Rik | 4930413F20Rik RIKEN cDNA 4930413F20 gene | 0.11 | 0.08 | 0.03 | 0.12 | 0.71 | 0.33 |
| Gm38666 | Gm38666 predicted gene, 38666 | 0.11 | 0.08 | 0.03 | 0.12 | 0.71 | 0.33 |
| Nlrc4 | Nlrc4 NLR family, CARD domain containing 4 | 0.14 | 0.08 | 0.03 | 0.12 | 0.56 | 0.33 |
| Mir5112 | Mir5112 microRNA 5112 | 0.14 | 0.08 | 0.03 | 0.12 | 0.56 | 0.33 |
| Trim75 | Trim75 tripartite motif-containing 75 | 0.25 | 0.08 | 0.03 | 0.12 | 0.31 | 0.33 |
| Mir8103 | Mir8103 microRNA 8103 | 0.06 | 0.08 | 0.03 | 0.14 | 1.41 | 0.33 |
| A730018C14Rik | A730018C14Rik RIKEN cDNA A730018C14 gene | 0.06 | 0.08 | 0.03 | 0.14 | 1.41 | 0.33 |
| Supt4b | Supt4b suppressor of Ty 4B | 0.44 | 0.78 | 0.26 | 0.63 | 1.77 | 0.33 |
| Gm5083 | Gm5083 predicted gene 5083 | 0.44 | 0.47 | 0.15 | 0.12 | 1.06 | 0.33 |
| C7 | C7 complement component 7 | 0.22 | 0.47 | 0.15 | 0.20 | 2.12 | 0.33 |
| Lrriq3 | Lrriq3 leucine-rich repeats and IQ motif containing 3 | 0.25 | 0.47 | 0.15 | 0.23 | 1.88 | 0.33 |
| Mb | Mb myoglobin | 0.53 | 0.47 | 0.15 | 0.26 | 0.89 | 0.33 |
| 1810009A15Rik | 1810009A15Rik RIKEN cDNA 1810009A15 gene | 0.39 | 0.63 | 0.20 | 0.52 | 1.61 | 0.33 |
| Slc25a43 | Slc25a43 solute carrier family 25, member 43 | 0.19 | 0.16 | 0.05 | 0.03 | 0.81 | 0.33 |
| Akr1c14 | Akr1c14 aldo-keto reductase family 1, member C14 | 1.25 | 0.16 | 0.05 | 0.03 | 0.13 | 0.33 |
| 1700018A04Rik | 1700018A04Rik RIKEN cDNA 1700018A04 gene | 0.08 | 0.16 | 0.05 | 0.06 | 1.88 | 0.33 |
| Mir7b | Mir7b microRNA 7b | 0.14 | 0.16 | 0.05 | 0.06 | 1.13 | 0.33 |
| Ffar4 | Ffar4 free fatty acid receptor 4 | 0.14 | 0.16 | 0.05 | 0.06 | 1.13 | 0.33 |
| Vmn2r85 | Vmn2r85 vomeronasal 2, receptor 85 | 0.25 | 0.16 | 0.05 | 0.09 | 0.63 | 0.33 |
| Serpina10 | Serpina10 serine (or cysteine) peptidase inhibitor, clade A (alpha-1 antiproteinase, antitrypsin), member 10 | 0.03 | 0.16 | 0.05 | 0.14 | 5.65 | 0.33 |
| Adam7 | Adam7 a disintegrin and metallopeptidase domain 7 | 0.06 | 0.16 | 0.05 | 0.14 | 2.82 | 0.33 |
| Cyb5r2 | Cyb5r2 cytochrome b5 reductase 2 | 0.30 | 0.16 | 0.05 | 0.14 | 0.51 | 0.33 |
| Sost | Sost sclerostin | 0.11 | 0.16 | 0.05 | 0.17 | 1.41 | 0.33 |
| Mfsd4b5 | Mfsd4b5 major facilitator superfamily domain containing 4B5 | 0.19 | 0.16 | 0.05 | 0.17 | 0.81 | 0.33 |
| Spo11 | Spo11 SPO11 meiotic protein covalently bound to DSB | 0.14 | 0.16 | 0.05 | 0.20 | 1.13 | 0.33 |
| Pklr | Pklr pyruvate kinase liver and red blood cell | 0.25 | 0.16 | 0.05 | 0.23 | 0.63 | 0.33 |
| Tnni2 | Tnni2 troponin I, skeletal, fast 2 | 0.36 | 0.16 | 0.05 | 0.23 | 0.43 | 0.33 |
| AV051173 | AV051173 expressed sequence AV051173 | 0.22 | 0.16 | 0.05 | 0.29 | 0.71 | 0.33 |
| Myct1 | Myct1 myc target 1 | 1.22 | 0.55 | 0.18 | 0.20 | 0.45 | 0.33 |
| Fendrr | Fendrr Foxf1 adjacent non-coding developmental regulatory RNA | 1.25 | 0.55 | 0.18 | 0.43 | 0.44 | 0.33 |
| Gm10466 | Gm10466 predicted gene 10466 | 0.22 | 0.23 | 0.08 | 0.09 | 1.06 | 0.33 |
| Speer4b | Speer4b spermatogenesis associated glutamate (E)-rich protein 4B | 0.17 | 0.23 | 0.08 | 0.14 | 1.41 | 0.33 |
| 2610037D02Rik | 2610037D02Rik RIKEN cDNA 2610037D02 gene | 0.19 | 0.23 | 0.08 | 0.17 | 1.21 | 0.33 |
| Ccdc89 | Ccdc89 coiled-coil domain containing 89 | 0.61 | 0.39 | 0.13 | 0.35 | 0.64 | 0.33 |
| 5033406O09Rik | 5033406O09Rik RIKEN cDNA 5033406O09 gene | 0.64 | 1.10 | 0.36 | 0.43 | 1.72 | 0.33 |
| Zfp366 | Zfp366 zinc finger protein 366 | 2.91 | 2.74 | 0.87 | 0.69 | 0.94 | 0.32 |
| Tex50 | Tex50 testis expressed 50 | 1.03 | 0.81 | 0.26 | 0.40 | 0.79 | 0.32 |
| Eps8l1 | Eps8l1 EPS8-like 1 | 5.57 | 8.24 | 2.58 | 4.34 | 1.48 | 0.31 |
| Prokr2 | Prokr2 prokineticin receptor 2 | 2.74 | 2.95 | 0.92 | 0.89 | 1.07 | 0.31 |
| Pkd1l2 | Pkd1l2 polycystic kidney disease 1 like 2 | 0.33 | 0.57 | 0.18 | 0.35 | 1.73 | 0.31 |
| AV039307 | AV039307 expressed sequence AV039307 | 0.44 | 0.57 | 0.18 | 0.35 | 1.29 | 0.31 |
| Bfsp1 | Bfsp1 beaded filament structural protein 1, in lens-CP94 | 0.53 | 0.50 | 0.15 | 0.55 | 0.94 | 0.31 |
| Aif1 | Aif1 allograft inflammatory factor 1 | 3.55 | 4.46 | 1.38 | 1.99 | 1.26 | 0.31 |
| 2310034G01Rik | 2310034G01Rik RIKEN cDNA 2310034G01 gene | 0.72 | 0.91 | 0.28 | 0.40 | 1.27 | 0.31 |
| Tubd1 | Tubd1 tubulin, delta 1 | 5.51 | 4.88 | 1.48 | 2.53 | 0.88 | 0.30 |
| Gm15972 | Gm15972 predicted gene 15972 | 0.39 | 0.68 | 0.20 | 0.35 | 1.75 | 0.30 |
| Snhg9 | Snhg9 small nucleolar RNA host gene 9 | 0.08 | 0.34 | 0.10 | 0.12 | 4.08 | 0.30 |
| Wnt3a | Wnt3a wingless-type MMTV integration site family, member 3A | 0.28 | 0.34 | 0.10 | 0.12 | 1.22 | 0.30 |
| A930009A15Rik | A930009A15Rik RIKEN cDNA A930009A15 gene | 0.33 | 0.34 | 0.10 | 0.26 | 1.02 | 0.30 |
| Dio3os | Dio3os deiodinase, iodothyronine type III, opposite strand | 1.19 | 2.37 | 0.72 | 1.09 | 1.99 | 0.30 |
| Zfp882 | Zfp882 zinc finger protein 882 | 6.93 | 4.88 | 1.46 | 2.91 | 0.70 | 0.30 |
| D930020B18Rik | D930020B18Rik RIKEN cDNA D930020B18 gene | 0.80 | 0.86 | 0.26 | 0.29 | 1.07 | 0.30 |
| Il1b | Il1b interleukin 1 beta | 0.17 | 1.90 | 0.56 | 0.92 | 11.46 | 0.30 |
| Zfp979 | Zfp979 zinc finger protein 979 | 0.47 | 0.52 | 0.15 | 0.14 | 1.11 | 0.29 |
| Rnase6 | Rnase6 ribonuclease, RNase A family, 6 | 0.11 | 0.26 | 0.08 | 0.14 | 2.35 | 0.29 |
| 9330117O12Rik | 9330117O12Rik RIKEN cDNA 9330117O12 gene | 1.16 | 0.26 | 0.08 | 0.14 | 0.22 | 0.29 |
| Pdia2 | Pdia2 protein disulfide isomerase associated 2 | 0.58 | 0.26 | 0.08 | 0.37 | 0.45 | 0.29 |
| Prss8 | Prss8 protease, serine 8 (prostasin) | 0.86 | 1.49 | 0.43 | 1.84 | 1.73 | 0.29 |
| Hes5 | Hes5 hes family bHLH transcription factor 5 | 27.93 | 25.65 | 7.49 | 10.64 | 0.92 | 0.29 |
| Srarp | Srarp steroid receptor associated and regulated protein | 2.66 | 5.27 | 1.53 | 1.18 | 1.98 | 0.29 |
| Cd209f | Cd209f CD209f antigen | 0.64 | 0.70 | 0.20 | 0.06 | 1.11 | 0.29 |
| Gm10012 | Gm10012 predicted pseudogene 10012 | 0.42 | 0.63 | 0.18 | 0.32 | 1.51 | 0.29 |
| Senp8 | Senp8 SUMO/sentrin specific peptidase 8 | 5.60 | 5.74 | 1.64 | 3.14 | 1.03 | 0.28 |
| 6430584L05Rik | 6430584L05Rik RIKEN cDNA 6430584L05 gene | 1.72 | 1.72 | 0.49 | 0.66 | 1.00 | 0.28 |
| Tpo | Tpo thyroid peroxidase | 0.30 | 0.18 | 0.05 | 0.03 | 0.60 | 0.28 |
| Dgat2l6 | Dgat2l6 diacylglycerol O-acyltransferase 2-like 6 | 0.33 | 0.37 | 0.10 | 0.03 | 1.10 | 0.28 |
| 1700063O14Rik | 1700063O14Rik RIKEN cDNA 1700063O14 gene | 0.08 | 0.37 | 0.10 | 0.06 | 4.39 | 0.28 |
| Glra1 | Glra1 glycine receptor, alpha 1 subunit | 0.14 | 0.18 | 0.05 | 0.09 | 1.32 | 0.28 |
| Tspan1 | Tspan1 tetraspanin 1 | 0.17 | 0.18 | 0.05 | 0.09 | 1.10 | 0.28 |
| NA | NA | 0.17 | 0.18 | 0.05 | 0.09 | 1.10 | 0.28 |
| Pik3c2g | Pik3c2g phosphatidylinositol-4-phosphate 3-kinase catalytic subunit type 2 gamma | 0.19 | 0.18 | 0.05 | 0.09 | 0.94 | 0.28 |
| Tfap2c | Tfap2c transcription factor AP-2, gamma | 0.17 | 0.18 | 0.05 | 0.14 | 1.10 | 0.28 |
| Mir208a | Mir208a microRNA 208a | 0.19 | 0.18 | 0.05 | 0.14 | 0.94 | 0.28 |
| Fam83f | Fam83f family with sequence similarity 83, member F | 0.06 | 0.18 | 0.05 | 0.17 | 3.30 | 0.28 |
| Gm20743 | Gm20743 predicted gene, 20743 | 0.94 | 0.91 | 0.26 | 0.17 | 0.97 | 0.28 |
| Tex11 | Tex11 testis expressed gene 11 | 0.33 | 0.18 | 0.05 | 0.23 | 0.55 | 0.28 |
| Gm14005 | Gm14005 predicted gene 14005 | 0.39 | 0.37 | 0.10 | 0.23 | 0.94 | 0.28 |
| Barhl2 | Barhl2 BarH like homeobox 2 | 0.08 | 0.18 | 0.05 | 0.40 | 2.20 | 0.28 |
| Trh | Trh thyrotropin releasing hormone | 0.75 | 5.95 | 1.66 | 4.23 | 7.95 | 0.28 |
| Kctd21 | Kctd21 potassium channel tetramerisation domain containing 21 | 17.24 | 11.27 | 3.14 | 5.18 | 0.65 | 0.28 |
| Bin2 | Bin2 bridging integrator 2 | 3.69 | 4.62 | 1.28 | 1.44 | 1.25 | 0.28 |
| Crip3 | Crip3 cysteine-rich protein 3 | 0.91 | 1.30 | 0.36 | 0.52 | 1.43 | 0.27 |
| Tm6sf2 | Tm6sf2 transmembrane 6 superfamily member 2 | 2.44 | 1.41 | 0.38 | 0.26 | 0.58 | 0.27 |
| Ccdc42 | Ccdc42 coiled-coil domain containing 42 | 0.39 | 0.94 | 0.26 | 0.55 | 2.42 | 0.27 |
| Cpsf4l | Cpsf4l cleavage and polyadenylation specific factor 4-like | 1.50 | 1.51 | 0.41 | 0.58 | 1.01 | 0.27 |
| Cstad | Cstad CSA-conditional, T cell activation-dependent protein | 1.69 | 1.62 | 0.43 | 1.04 | 0.96 | 0.27 |
| Fam228a | Fam228a family with sequence similarity 228, member A | 1.91 | 2.19 | 0.59 | 1.04 | 1.15 | 0.27 |
| Cyp26c1 | Cyp26c1 cytochrome P450, family 26, subfamily c, polypeptide 1 | 0.03 | 0.29 | 0.08 | 0.06 | 10.36 | 0.27 |
| A930003O13Rik | A930003O13Rik RIKEN cDNA A930003O13 gene | 0.22 | 0.29 | 0.08 | 0.12 | 1.29 | 0.27 |
| Aoc1 | Aoc1 amine oxidase, copper-containing 1 | 0.14 | 0.29 | 0.08 | 0.20 | 2.07 | 0.27 |
| Vmn2r84 | Vmn2r84 vomeronasal 2, receptor 84 | 0.55 | 0.29 | 0.08 | 0.23 | 0.52 | 0.27 |
| Gabrr1 | Gabrr1 gamma-aminobutyric acid (GABA) C receptor, subunit rho 1 | 0.08 | 0.29 | 0.08 | 0.43 | 3.45 | 0.27 |
| Ccr5 | Ccr5 chemokine (C-C motif) receptor 5 | 2.99 | 2.11 | 0.56 | 0.81 | 0.71 | 0.27 |
| Tbxas1 | Tbxas1 thromboxane A synthase 1, platelet | 2.52 | 2.53 | 0.66 | 1.04 | 1.00 | 0.26 |
| Gm5086 | Gm5086 predicted gene 5086 | 0.53 | 0.39 | 0.10 | 0.09 | 0.74 | 0.26 |
| 4930447N08Rik | 4930447N08Rik RIKEN cDNA 4930447N08 gene | 0.39 | 0.39 | 0.10 | 0.14 | 1.01 | 0.26 |
| Zfp808 | Zfp808 zinc finger protein 808 | 0.33 | 0.39 | 0.10 | 0.20 | 1.18 | 0.26 |
| Ackr4 | Ackr4 atypical chemokine receptor 4 | 0.72 | 0.39 | 0.10 | 0.35 | 0.54 | 0.26 |
| Rgs13 | Rgs13 regulator of G-protein signaling 13 | 0.42 | 0.89 | 0.23 | 0.40 | 2.13 | 0.26 |
| Mir7052 | Mir7052 microRNA 7052 | 1.22 | 0.99 | 0.26 | 0.43 | 0.81 | 0.26 |
| Tmco5 | Tmco5 transmembrane and coiled-coil domains 5 | 0.97 | 0.50 | 0.13 | 0.17 | 0.51 | 0.26 |
| Slco1a4 | Slco1a4 solute carrier organic anion transporter family, member 1a4 | 43.31 | 45.63 | 11.76 | 12.49 | 1.05 | 0.26 |
| Gm14827 | Gm14827 predicted gene 14827 | 2.47 | 2.19 | 0.56 | 0.63 | 0.89 | 0.26 |
| Gm16386 | Gm16386 predicted gene 16386 | 2.30 | 2.19 | 0.56 | 1.15 | 0.95 | 0.26 |
| Nlrp5-ps | Nlrp5-ps NLR family, pyrin domain containing 5, pseudogene | 0.53 | 0.70 | 0.18 | 0.09 | 1.34 | 0.25 |
| C330024D21Rik | C330024D21Rik RIKEN cDNA C330024D21 gene | 0.03 | 0.10 | 0.03 | 0.03 | 3.77 | 0.24 |
| Rnf148 | Rnf148 ring finger protein 148 | 0.03 | 0.10 | 0.03 | 0.03 | 3.77 | 0.24 |
| Mir6385 | Mir6385 microRNA 6385 | 0.03 | 0.10 | 0.03 | 0.03 | 3.77 | 0.24 |
| C230024C17Rik | C230024C17Rik RIKEN cDNA C230024C17 gene | 0.06 | 0.10 | 0.03 | 0.03 | 1.88 | 0.24 |
| Syt8 | Syt8 synaptotagmin VIII | 0.06 | 0.10 | 0.03 | 0.03 | 1.88 | 0.24 |
| Pbld1 | Pbld1 phenazine biosynthesis-like protein domain containing 1 | 0.06 | 0.10 | 0.03 | 0.03 | 1.88 | 0.24 |
| Scarna17 | Scarna17 small Cajal body-specific RNA 17 | 0.06 | 0.10 | 0.03 | 0.03 | 1.88 | 0.24 |
| 4930443O20Rik | 4930443O20Rik RIKEN cDNA 4930443O20 gene | 0.11 | 0.10 | 0.03 | 0.03 | 0.94 | 0.24 |
| Card11 | Card11 caspase recruitment domain family, member 11 | 0.22 | 0.21 | 0.05 | 0.03 | 0.94 | 0.24 |
| Gm6249 | Gm6249 predicted gene 6249 | 0.03 | 0.10 | 0.03 | 0.06 | 3.77 | 0.24 |
| Leap2 | Leap2 liver-expressed antimicrobial peptide 2 | 0.03 | 0.10 | 0.03 | 0.06 | 3.77 | 0.24 |
| Pdcd1 | Pdcd1 programmed cell death 1 | 0.08 | 0.10 | 0.03 | 0.06 | 1.26 | 0.24 |
| Lrat | Lrat lecithin-retinol acyltransferase (phosphatidylcholine-retinol-O-acyltransferase) | 0.14 | 0.10 | 0.03 | 0.06 | 0.75 | 0.24 |
| Aqp2 | Aqp2 aquaporin 2 | 0.03 | 0.10 | 0.03 | 0.09 | 3.77 | 0.24 |
| Tspear | Tspear thrombospondin type laminin G domain and EAR repeats | 0.06 | 0.10 | 0.03 | 0.09 | 1.88 | 0.24 |
| Pfn3 | Pfn3 profilin 3 | 0.06 | 0.21 | 0.05 | 0.09 | 3.77 | 0.24 |
| Krt86 | Krt86 keratin 86 | 0.08 | 0.10 | 0.03 | 0.09 | 1.26 | 0.24 |
| 2610028H24Rik | 2610028H24Rik RIKEN cDNA 2610028H24 gene | 0.17 | 0.10 | 0.03 | 0.09 | 0.63 | 0.24 |
| Ces1d | Ces1d carboxylesterase 1D | 0.19 | 0.10 | 0.03 | 0.09 | 0.54 | 0.24 |
| Rpe65 | Rpe65 retinal pigment epithelium 65 | 0.30 | 0.10 | 0.03 | 0.09 | 0.34 | 0.24 |
| Grifin | Grifin galectin-related inter-fiber protein | 0.19 | 0.42 | 0.10 | 0.12 | 2.15 | 0.24 |
| Otog | Otog otogelin | 0.03 | 0.10 | 0.03 | 0.14 | 3.77 | 0.24 |
| Hgd | Hgd homogentisate 1, 2-dioxygenase | 0.03 | 0.10 | 0.03 | 0.14 | 3.77 | 0.24 |
| Zswim2 | Zswim2 zinc finger SWIM-type containing 2 | 0.08 | 0.10 | 0.03 | 0.14 | 1.26 | 0.24 |
| Krt17 | Krt17 keratin 17 | 0.08 | 0.21 | 0.05 | 0.14 | 2.51 | 0.24 |
| Tmem26 | Tmem26 transmembrane protein 26 | 0.03 | 0.21 | 0.05 | 0.17 | 7.53 | 0.24 |
| Dppa1 | Dppa1 developmental pluripotency associated 1 | 0.08 | 0.10 | 0.03 | 0.23 | 1.26 | 0.24 |
| A730043L09Rik | A730043L09Rik RIKEN cDNA A730043L09 gene | 0.22 | 0.21 | 0.05 | 0.23 | 0.94 | 0.24 |
| Lhx1os | Lhx1os LIM homeobox 1, opposite strand | 0.17 | 0.21 | 0.05 | 0.26 | 1.26 | 0.24 |
| Gpr139 | Gpr139 G protein-coupled receptor 139 | 0.22 | 0.21 | 0.05 | 0.26 | 0.94 | 0.24 |
| Gm11201 | Gm11201 predicted gene 11201 | 3.13 | 3.03 | 0.74 | 1.70 | 0.97 | 0.24 |
| Tmc5 | Tmc5 transmembrane channel-like gene family 5 | 0.19 | 0.31 | 0.08 | 0.03 | 1.61 | 0.24 |
| BC049352 | BC049352 cDNA sequence BC049352 | 0.03 | 0.31 | 0.08 | 0.12 | 11.30 | 0.24 |
| 9130008F23Rik | 9130008F23Rik RIKEN cDNA 9130008F23 gene | 0.19 | 0.31 | 0.08 | 0.29 | 1.61 | 0.24 |
| Ankrd66 | Ankrd66 ankyrin repeat domain 66 | 0.42 | 0.63 | 0.15 | 0.20 | 1.51 | 0.24 |
| Zfp976 | Zfp976 zinc finger protein 976 | 1.00 | 1.38 | 0.33 | 0.69 | 1.39 | 0.24 |
| 4930578C19Rik | 4930578C19Rik RIKEN cDNA 4930578C19 gene | 0.83 | 0.65 | 0.15 | 0.17 | 0.78 | 0.24 |
| 0610005C13Rik | 0610005C13Rik RIKEN cDNA 0610005C13 gene | 0.39 | 0.55 | 0.13 | 0.40 | 1.41 | 0.23 |
| Snord32a | Snord32a small nucleolar RNA, C/D box 32A | 0.08 | 0.34 | 0.08 | 0.43 | 4.08 | 0.23 |
| Thegl | Thegl theg spermatid protein like | 0.58 | 0.47 | 0.10 | 0.29 | 0.81 | 0.22 |
| Mir496a | Mir496a microRNA 496a | 0.28 | 0.23 | 0.05 | 0.12 | 0.85 | 0.22 |
| 4933432I09Rik | 4933432I09Rik RIKEN cDNA 4933432I09 gene | 1.36 | 0.83 | 0.18 | 0.49 | 0.61 | 0.21 |
| Ccdc121 | Ccdc121 coiled-coil domain containing 121 | 0.89 | 0.60 | 0.13 | 0.37 | 0.68 | 0.21 |
| Nhlrc4 | Nhlrc4 NHL repeat containing 4 | 0.75 | 0.37 | 0.08 | 0.06 | 0.49 | 0.21 |
| Nabp1 | Nabp1 nucleic acid binding protein 1 | 11.17 | 8.98 | 1.87 | 3.65 | 0.80 | 0.21 |
| 5830418P13Rik | 5830418P13Rik RIKEN cDNA 5830418P13 gene | 0.28 | 0.50 | 0.10 | 0.17 | 1.79 | 0.21 |
| A630077J23Rik | A630077J23Rik RIKEN cDNA A630077J23 gene | 0.06 | 0.13 | 0.03 | 0.03 | 2.35 | 0.20 |
| Chrna6 | Chrna6 cholinergic receptor, nicotinic, alpha polypeptide 6 | 0.08 | 0.13 | 0.03 | 0.06 | 1.57 | 0.20 |
| Car5a | Car5a carbonic anhydrase 5a, mitochondrial | 0.33 | 0.13 | 0.03 | 0.06 | 0.39 | 0.20 |
| Snord88a | Snord88a small nucleolar RNA, C/D box 88A | 0.06 | 0.13 | 0.03 | 0.09 | 2.35 | 0.20 |
| Gm5134 | Gm5134 predicted gene 5134 | 0.11 | 0.13 | 0.03 | 0.09 | 1.18 | 0.20 |
| Gpr82 | Gpr82 G protein-coupled receptor 82 | 0.14 | 0.13 | 0.03 | 0.09 | 0.94 | 0.20 |
| **Orm2** | **Orm2 orosomucoid 2** | **0.03** | **0.13** | **0.03** | **0.12** | **4.71** | **0.20** |
| Mir1949 | Mir1949 microRNA 1949 | 0.11 | 0.13 | 0.03 | 0.12 | 1.18 | 0.20 |
| Ccr2 | Ccr2 chemokine (C-C motif) receptor 2 | 0.19 | 0.13 | 0.03 | 0.12 | 0.67 | 0.20 |
| Nxf3 | Nxf3 nuclear RNA export factor 3 | 0.03 | 0.13 | 0.03 | 0.14 | 4.71 | 0.20 |
| D730005E14Rik | D730005E14Rik RIKEN cDNA D730005E14 gene | 0.14 | 0.78 | 0.15 | 0.43 | 5.65 | 0.20 |
| 4931440J10Rik | 4931440J10Rik RIKEN cDNA 4931440J10 gene | 0.22 | 0.26 | 0.05 | 0.06 | 1.18 | 0.20 |
| Nmur1 | Nmur1 neuromedin U receptor 1 | 0.08 | 0.26 | 0.05 | 0.09 | 3.14 | 0.20 |
| NA | NA | 0.19 | 0.26 | 0.05 | 0.09 | 1.35 | 0.20 |
| Gm5084 | Gm5084 predicted gene 5084 | 0.14 | 0.26 | 0.05 | 0.12 | 1.88 | 0.20 |
| Slc38a8 | Slc38a8 solute carrier family 38, member 8 | 0.19 | 0.39 | 0.08 | 0.12 | 2.02 | 0.20 |
| Mir1258 | Mir1258 microRNA 1258 | 0.17 | 0.26 | 0.05 | 0.17 | 1.57 | 0.20 |
| Ppp1r42 | Ppp1r42 protein phosphatase 1, regulatory subunit 42 | 0.58 | 0.26 | 0.05 | 0.17 | 0.45 | 0.20 |
| Gm2721 | Gm2721 predicted gene 2721 | 0.28 | 0.26 | 0.05 | 0.20 | 0.94 | 0.20 |
| Dkk2 | Dkk2 dickkopf WNT signaling pathway inhibitor 2 | 0.44 | 0.39 | 0.08 | 0.23 | 0.88 | 0.20 |
| Ly6i | Ly6i lymphocyte antigen 6 complex, locus I | 0.06 | 0.52 | 0.10 | 0.46 | 9.42 | 0.20 |
| Oxt | Oxt oxytocin | 0.11 | 0.52 | 0.10 | 0.52 | 4.71 | 0.20 |
| Ly9 | Ly9 lymphocyte antigen 9 | 0.25 | 0.55 | 0.10 | 0.26 | 2.20 | 0.19 |
| Gm15997 | Gm15997 predicted gene 15997 | 0.36 | 0.42 | 0.08 | 0.32 | 1.16 | 0.18 |
| Prl | Prl prolactin | 2.91 | 2.95 | 0.54 | 0.40 | 1.01 | 0.18 |
| 4732416N19Rik | 4732416N19Rik RIKEN cDNA 4732416N19 gene | 0.14 | 0.29 | 0.05 | 0.03 | 2.07 | 0.18 |
| 6430503K07Rik | 6430503K07Rik RIKEN cDNA 6430503K07 gene | 0.50 | 0.29 | 0.05 | 0.03 | 0.58 | 0.18 |
| Rtp2 | Rtp2 receptor transporter protein 2 | 0.19 | 0.29 | 0.05 | 0.12 | 1.48 | 0.18 |
| Slamf6 | Slamf6 SLAM family member 6 | 0.25 | 0.29 | 0.05 | 0.12 | 1.15 | 0.18 |
| Lrrd1 | Lrrd1 leucine rich repeats and death domain containing 1 | 0.30 | 0.29 | 0.05 | 0.12 | 0.94 | 0.18 |
| Trim6 | Trim6 tripartite motif-containing 6 | 0.08 | 0.29 | 0.05 | 0.17 | 3.45 | 0.18 |
| Il25 | Il25 interleukin 25 | 0.67 | 0.29 | 0.05 | 0.20 | 0.43 | 0.18 |
| Zfp493 | Zfp493 zinc finger protein 493 | 3.10 | 2.61 | 0.46 | 0.95 | 0.84 | 0.18 |
| 6530411M01Rik | 6530411M01Rik RIKEN cDNA 6530411M01 gene | 0.83 | 1.02 | 0.18 | 0.52 | 1.22 | 0.18 |
| Slc19a3 | Slc19a3 solute carrier family 19, member 3 | 2.77 | 1.75 | 0.31 | 0.29 | 0.63 | 0.18 |
| Slc23a3 | Slc23a3 solute carrier family 23 (nucleobase transporters), member 3 | 1.11 | 1.23 | 0.20 | 0.72 | 1.11 | 0.17 |
| Pla2g4b | Pla2g4b phospholipase A2, group IVB (cytosolic) | 0.28 | 0.31 | 0.05 | 0.03 | 1.13 | 0.16 |
| A730046J19Rik | A730046J19Rik RIKEN cDNA A730046J19 gene | 0.17 | 0.31 | 0.05 | 0.06 | 1.88 | 0.16 |
| Klk12 | Klk12 kallikrein related-peptidase 12 | 0.03 | 0.16 | 0.03 | 0.03 | 5.65 | 0.16 |
| A430078G23Rik | A430078G23Rik RIKEN cDNA A430078G23 gene | 0.14 | 0.16 | 0.03 | 0.06 | 1.13 | 0.16 |
| 1700016L04Rik | 1700016L04Rik RIKEN cDNA 1700016L04 gene | 0.14 | 0.16 | 0.03 | 0.06 | 1.13 | 0.16 |
| Mir5119 | Mir5119 microRNA 5119 | 0.22 | 0.16 | 0.03 | 0.06 | 0.71 | 0.16 |
| 4930467D21Rik | 4930467D21Rik RIKEN cDNA 4930467D21 gene | 0.06 | 0.16 | 0.03 | 0.09 | 2.82 | 0.16 |
| Tsix | Tsix X (inactive)-specific transcript, opposite strand | 0.33 | 0.16 | 0.03 | 0.09 | 0.47 | 0.16 |
| BC147527 | BC147527 cDNA sequence BC147527 | 0.14 | 0.16 | 0.03 | 0.12 | 1.13 | 0.16 |
| Mir1943 | Mir1943 microRNA 1943 | 0.25 | 0.16 | 0.03 | 0.23 | 0.63 | 0.16 |
| Clec7a | Clec7a C-type lectin domain family 7, member a | 0.22 | 0.50 | 0.08 | 0.03 | 2.24 | 0.15 |
| Ttc29 | Ttc29 tetratricopeptide repeat domain 29 | 0.53 | 0.34 | 0.05 | 0.17 | 0.64 | 0.15 |
| Hist1h2ae | Hist1h2ae histone cluster 1, H2ae | 0.22 | 0.18 | 0.03 | 0.03 | 0.82 | 0.14 |
| Chodl | Chodl chondrolectin | 0.22 | 0.37 | 0.05 | 0.03 | 1.65 | 0.14 |
| Ckm | Ckm creatine kinase, muscle | 0.25 | 0.18 | 0.03 | 0.03 | 0.73 | 0.14 |
| Apol7d | Apol7d apolipoprotein L 7d | 0.08 | 0.18 | 0.03 | 0.06 | 2.20 | 0.14 |
| Hist1h4c | Hist1h4c histone cluster 1, H4c | 0.14 | 0.18 | 0.03 | 0.06 | 1.32 | 0.14 |
| Mir7050 | Mir7050 microRNA 7050 | 0.17 | 0.18 | 0.03 | 0.06 | 1.10 | 0.14 |
| H2-DMb2 | H2-DMb2 histocompatibility 2, class II, locus Mb2 | 0.39 | 0.37 | 0.05 | 0.26 | 0.94 | 0.14 |
| Lilra5 | Lilra5 leukocyte immunoglobulin-like receptor, subfamily A (with TM domain), member 5 | 0.14 | 0.42 | 0.05 | 0.26 | 3.01 | 0.12 |
| Gm20939 | Gm20939 predicted gene, 20939 | 0.44 | 0.44 | 0.05 | 0.20 | 1.00 | 0.12 |
| Aadat | Aadat aminoadipate aminotransferase | 0.11 | 0.44 | 0.05 | 0.26 | 4.00 | 0.12 |
| C130060C02Rik | C130060C02Rik RIKEN cDNA C130060C02 gene | 0.33 | 0.47 | 0.05 | 0.14 | 1.41 | 0.11 |
| R3hdml | R3hdml R3H domain containing-like | 0.11 | 0.26 | 0.03 | 0.03 | 2.35 | 0.10 |
| Mir453 | Mir453 microRNA 453 | 0.19 | 0.26 | 0.03 | 0.09 | 1.35 | 0.10 |
| Mir1188 | Mir1188 microRNA 1188 | 1.03 | 0.78 | 0.08 | 0.17 | 0.76 | 0.10 |
| Ccdc18 | Ccdc18 coiled-coil domain containing 18 | 0.44 | 0.83 | 0.08 | 0.60 | 1.88 | 0.09 |
| 1110015O18Rik | 1110015O18Rik RIKEN cDNA 1110015O18 gene | 0.19 | 0.29 | 0.03 | 0.09 | 1.48 | 0.09 |
| Xist | Xist inactive X specific transcripts | 442.32 | 487.11 | 39.92 | 326.96 | 1.10 | 0.08 |
| Bach2os | Bach2os BTB and CNC homology 2, opposite strand | 0.08 | 0.31 | 0.03 | 0.06 | 3.77 | 0.08 |
| Acss2os | Acss2os acyl-CoA synthetase short-chain family member 2, opposite strand | 0.33 | 0.31 | 0.03 | 0.06 | 0.94 | 0.08 |
| Mir341 | Mir341 microRNA 341 | 0.44 | 0.63 | 0.05 | 0.06 | 1.41 | 0.08 |
| Cyp19a1 | Cyp19a1 cytochrome P450, family 19, subfamily a, polypeptide 1 | 0.06 | 0.39 | 0.03 | 0.09 | 7.06 | 0.07 |
| Calcr | Calcr calcitonin receptor | 0.08 | 0.63 | 0.03 | 0.06 | 7.53 | 0.04 |
|  |  |  |  |  |  |  |  |
|  |  |  |  |  |  |  |  |
